# Matching Dietary Amino Acid Balance to the In Silico-Translated Exome Optimizes Growth and Reproduction without Cost to Lifespan

**DOI:** 10.1016/j.cmet.2017.02.005

**Published:** 2017-03-07

**Authors:** Matthew D.W. Piper, George A. Soultoukis, Eric Blanc, Andrea Mesaros, Samantha L. Herbert, Paula Juricic, Xiaoli He, Ilian Atanassov, Hanna Salmonowicz, Mingyao Yang, Stephen J. Simpson, Carlos Ribeiro, Linda Partridge

**Affiliations:** 1 Institute of Healthy Ageing and Department of Genetics, Evolution, and Environment, University College London, London WC1E 6BT, UK; 2 Max Planck Institute for Biology of Ageing, Köln 50931, Germany; 3 Berlin Institute of Health, Berlin 10117, Germany; 4 Behavior and Metabolism Laboratory, Champalimaud Centre for the Unknown, Lisbon 1400-038, Portugal; 5 UCL Ear Institute, University College London, London WC1X 8EE, UK; 6 Institute of Animal Genetics and Breeding, Sichuan Agricultural University, Chengdu 611130, China; 7 Charles Perkins Centre, School of Life and Environmental Sciences, University of Sydney, Sydney 2050, Australia

**Keywords:** amino acids, diet balance, dietary restriction, fitness, trade-off, reproduction, lifespan, growth, mouse, *Drosophila*

## Abstract

Balancing the quantity and quality of dietary protein relative to other nutrients is a key determinant of evolutionary fitness. A theoretical framework for defining a balanced diet would both reduce the enormous workload to optimize diets empirically and represent a breakthrough toward tailoring diets to the needs of consumers. Here, we report a simple and powerful in silico technique that uses the genome information of an organism to define its dietary amino acid requirements. We show for the fruit fly *Drosophila melanogaster* that such “exome-matched” diets are more satiating, enhance growth, and increase reproduction relative to non-matched diets. Thus, early life fitness traits can be enhanced at low levels of dietary amino acids that do not impose a cost to lifespan. Exome matching also enhanced mouse growth, indicating that it can be applied to other organisms whose genome sequence is known.

## Highlights

• In silico genome translation defines amino acid ratios for exome-matched diets
• Exome-matched diets reduce ad libitum food intake in flies and mice
• Exome-matched diets enhance early life fitness in flies without lifespan cost
• Improved mouse growth on an exome-matched diet demonstrates its utility for mammals

## Graphical Abstract

**Figure:**
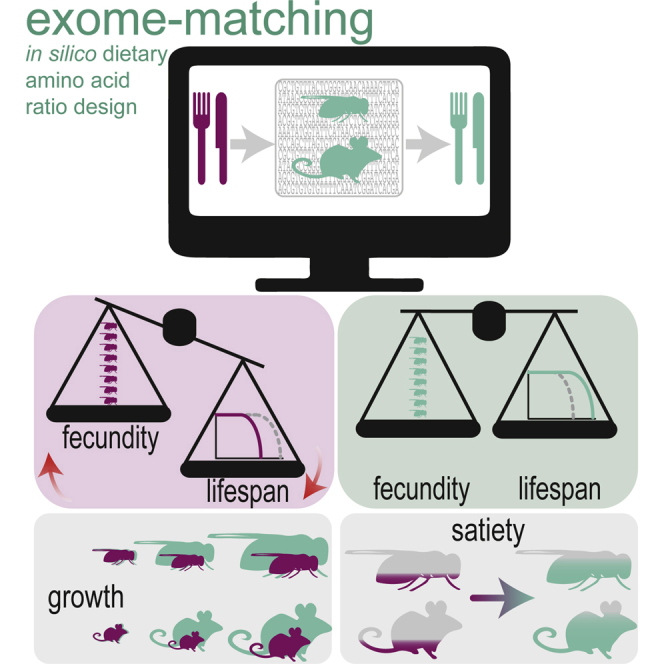

## Teaser

Dietary protein is a critical determinant of health, but the empirical determination of optimal amino acid ratios is challenging. Piper et al. show that a consumer’s genome provides a template for optimal dietary amino acid proportions. Low amounts of optimized protein are simultaneously beneficial for appetite, growth, reproduction, and lifespan.

## Introduction

Diets should ideally match the nutritional needs of their consumers for important life history traits such as growth, reproduction, and lifespan. However, quantifying a balanced diet is challenging given the large numbers of nutrients involved. Among the major macronutrients, the proportion of protein is especially important, since relatively high levels that are important to sustain early life fitness can also incur a heavy cost to lifespan (Le Couteur et al., 2016, Soultoukis and Partridge, 2016). Thus, establishing protein balance is critical for understanding how diets can be used to enhance lifelong health.

Many organisms possess mechanisms to prioritize and maintain protein intake to a narrow range of values that are higher than those optimal for longer-term health (Simpson and Raubenheimer, 2012). For example, when protein is relatively low in the diet, total food intake is elevated to maintain protein intake, causing overconsumption of other nutrients (Simpson and Raubenheimer, 2005), a situation thought to contribute to obesity. By contrast, when dietary protein content is high, total food consumption is curbed such that energy may be underconsumed—a formulation effectively exploited for weight loss, but also associated with shortened lifespan in insects, mice (Le Couteur et al., 2016, Lee et al., 2008, Solon-Biet et al., 2014), and humans (Levine et al., 2014). Thus, our evolutionary histories drive consumption of imbalanced foods in a manner that is associated with poor long-term health outcomes.

Investigations into why these trade-offs exist, and how they might be reduced, form an intense area of research into how dietary restriction (DR) extends healthy lifespan (Piper and Bartke, 2008). A long-held idea, derived from life history theory, is that DR improves lifespan by redirecting limiting resources away from reproduction toward somatic maintenance (Kirkwood, 1977, Williams, 1966). However, supplementing the DR diet with methionine (M) in flies can improve reproduction without any cost to lifespan (Dick et al., 2011, Grandison et al., 2009). Thus, enhancing dietary protein quality can increase early life fitness without compromising lifespan. However, understanding how to optimize dietary amino acid (AA) content is not trivial as it represents a 20-dimensional balancing problem. Whereas a theoretical framework is now established (Simpson and Raubenheimer, 2012), a quantitative, evidence-based approach to optimal dietary balance design that does not rely on empirical data has so far proved elusive. The discovery of such a definition for major dietary components would be transformative: diets could be designed to match the requirements of the consumer without the need for lengthy trials. Here we report such a theory for dietary AA balance.

## Results and Discussion

### In Silico Translation of the *Drosophila* Exome to Define Dietary AA Proportions

**Figure 1:**
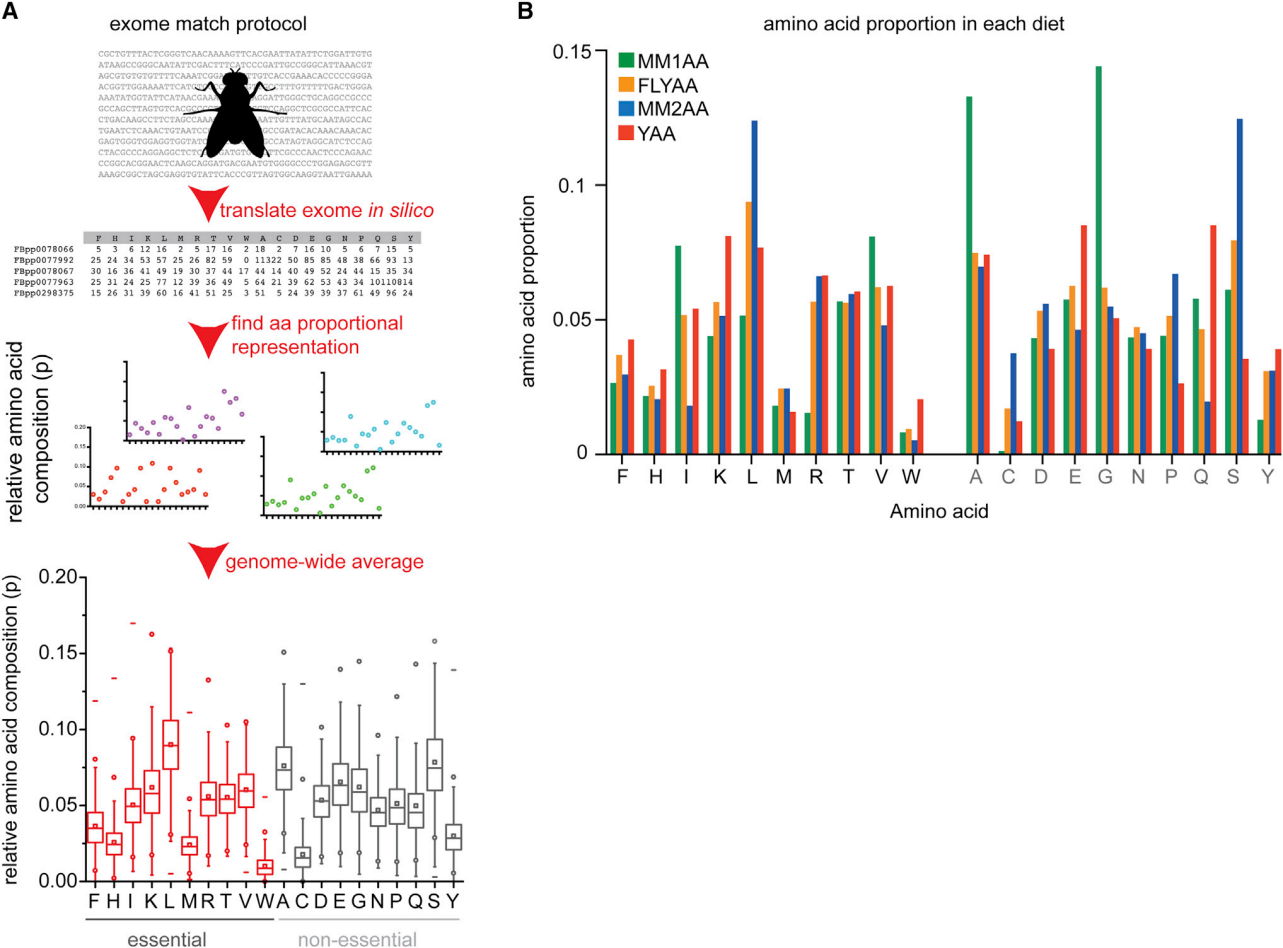
“Exome Matching” to Design Dietary AA Ratios (A) Computationally, we assembled the proportional representation of each amino acid (AA) in each of *D. melanogaster*’s 19,736 genes. From these, the mean proportional representation of each AA for all genes was determined. This “exome-matched” proportion is that in FLYAA. (B) Comparison of the relative abundance of each AA profile in this study: MM1AA (mismatch1 AA), FLYAA, MM2AA (mismatch2 AA), and YAA (yeast AA). The ten EAAs are listed first, followed by the non-essentials (gray text).

We hypothesized that the requirement of an animal for each AA is encoded by its genome. Using the *Drosophila melanogaster* genome, we translated in silico its 19,736 predicted protein-coding genes and derived the proportional representation of the 20 AAs (Figure 1A). This fly “exome-matched” AA ratio (FLYAA) was substantially different from an AA proportion previously referred to as HUNTAA during the development of a holidic diet (Hunt, 1970, Piper et al., 2014), referred to here as MM1AA (mismatched1 AA) (Figure 1B).

### Exome-Matched Diets Alter Feeding Behavior

**Figure 2:**
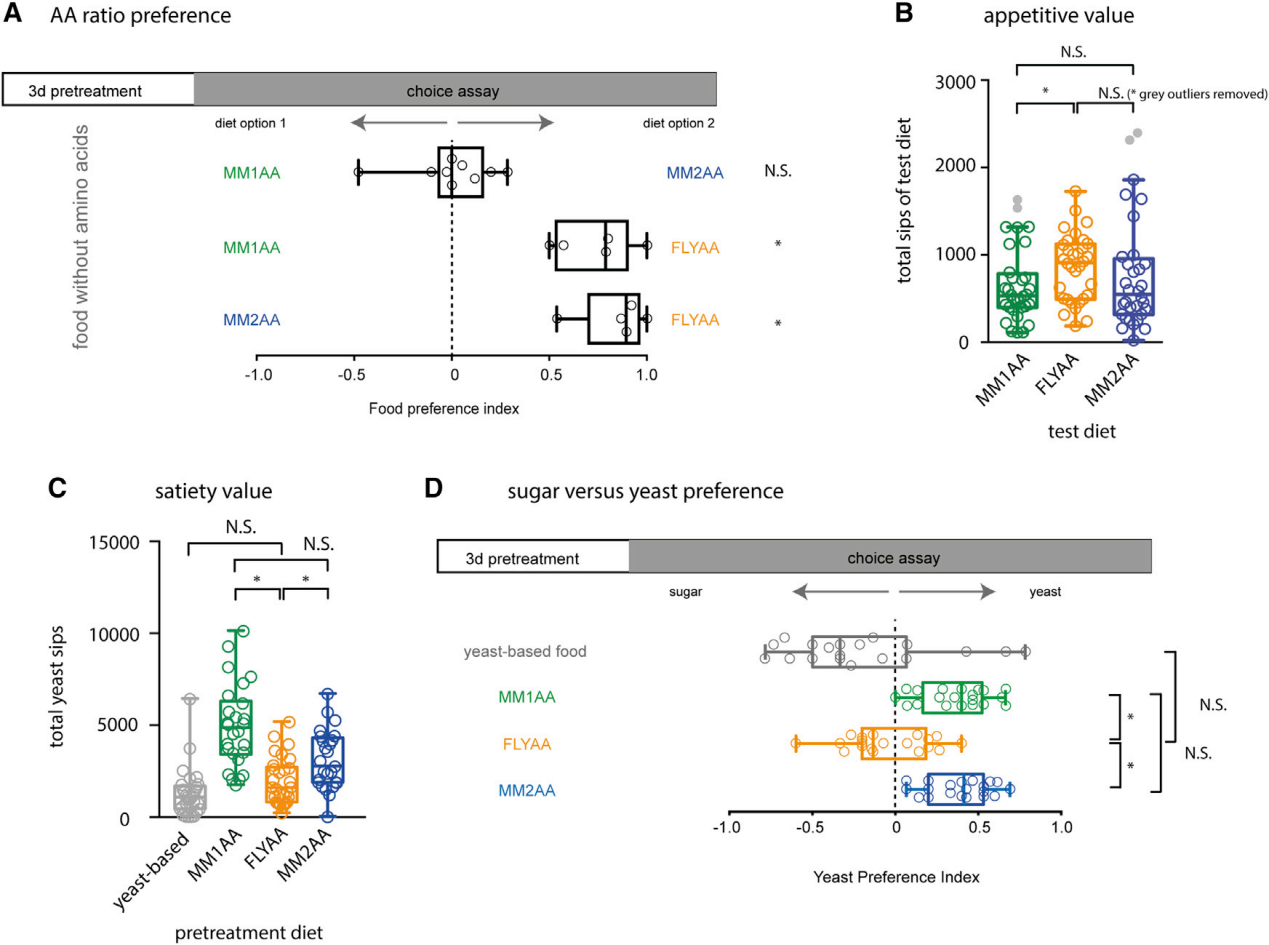
Effect of AA Ratio on *Drosophila* Feeding (A) Flies pre-starved for AAs were offered two diets differing only in the AA ratio (total mass fixed at 21.4 g/L). Flies preferred the matched ratio (FLYAA) over either MM1AA or MM2AA (p < 0.03 in all trials). No preference was observed when the choice was MM1AA versus MM2AA (p > 0.29 in 8 trials, p = 0.02 in 1 trial) (nine independent trials for MM1AA versus MM2AA, five independent trials each for FLYAA versus MM1AA and FLYAA versus MM2AA; chi-square test; 40 flies per assay). (B) After pre-feeding on a yeast-based diet, food intake of holidic medium was assessed using flyPAD. Flies on FLYAA ate significantly more (p = 0.03) than those on MM1AA, but not MM2AA (p = 0.06). After outlier (gray data points) removal, comparison of FLYAA versus MM2AA became significant (p = 0.02), while confidence in the difference between FLYAA and MM2AA increased (p = 0.01), indicating FLYAA has greater appetitive value than the MM diets (32 individually housed flies monitored per treatment; Wilcoxon rank-sum test and Tukey’s test for outlier detection). (C) Yeast intake was assessed using flyPAD after flies were pretreated on the indicated diets. Flies pretreated with either MM diet ate more than those pretreated with FLYAA or a yeast-based diet (p < 0.001 for all comparisons), indicating that FLYAA is more satiating than either MM diet (two independent trials with between 22 and 30 individually monitored flies per food type; linear model with trial and dietary pretreatment as fixed effects). (D) The preference of flies for yeast (higher yeast preference index, YPI) or sugar (lower YPI) was scored after pretreatment on each of the four diets indicated. FLYAA reduced YPI as effectively as yeast, and more so than either MM diet (20 independent trials for all conditions except 19 for yeast pretreatment; Dunn’s test for pairwise comparisons; ^∗^p < 0.05; N.S., not significant). See also Figure S1.

We tested if the matched diet (FLYAA) was perceived by flies as preferable to MM1AA. In a two-diet choice assay, in which the foods on offer were identical except that the ratio, but not the total mass, of AAs differed, female flies pretreated on AA-deficient food spent more time on FLYAA than MM1AA (Figure 2A). To assess if this preference was specific to the MM1AA versus FLYAA choice, we designed another ratio (MM2AA) that was equally mismatched to the fly exome translation as MM1AA, but with a different proportion of all 20 AAs (Figure 1B). Again, flies preferred FLYAA over MM2AA (Figure 2A). Interestingly, the flies exhibited no preference for MM1AA or MM2AA. These data indicate that in recovering from AA deficiency, flies that selected a food were not exhibiting indiscriminate food neophilia (Rozin, 1967), but instead specifically detected and chose the matched AA ratio over MM.

To assess the appetitive values of each ratio, we pre-fed all flies on a yeast-based diet and then measured their food intake when restricted to holidic media containing each of the three AA ratios. By pre-feeding a nutritious diet different from any of the test diets, we ensured that subsequent feeding decisions were neither biased by previous experience of the holidic medium, nor redressing a gross nutritional deficit. Using the automated fly feeding monitor flyPAD (Itskov et al., 2014) to track consumption for 1 hr, flies on FLYAA ate significantly more than those on MM1AA, and also weakly exceeded consumption on MM2AA (p = 0.06 down to 0.02 after outlier removal; Figure 2B). MM1AA consumption did not differ from MM2AA. FLYAA thus had a higher appetitive value than either MM ratio. The rapidity with which this behavior appeared shows that decisions about food consumption precede any possible changes in egg laying (see below). Thus, consistent with Corrales-Carvajal et al. (2016) and Walker et al. (2015), the internal nutritional state of the fly sets egg laying and feeding decisions in parallel, rather than in series.

Flies are thought to derive their protein from microbes, in particular yeasts, on decaying fruit (Markow, 2015). We assessed the potency of each of our AA ratios to affect yeast consumption using two different assays that have been used to infer satiety (Itskov et al., 2014, Ribeiro and Dickson, 2010). For both, flies were pre-fed one of the three AA ratios, and their yeast feeding was then assessed in either a no choice (using flyPAD; Figure 2C) or sugar/yeast (SY) choice situation (Ribeiro and Dickson, 2010; Figure 2D). In both assays, flies pre-fed the yeast-based diet or FLYAA ate less yeast than those pre-fed MM1AA or MM2AA (Figures 2C and 2D). Because we measured consumption of a common protein source (yeast) in the assays, these results indicate that yeast consumption specifically responds to AA status. Furthermore, the exome-matched diet was apparently an effective substitute for the natural proportion of AAs found in their ecologically relevant context (i.e., yeast) because pretreatment with FLYAA or a yeast-based diet suppressed yeast intake to the same extent (Figures 2C and 2D). Finally, the two MM diets were similarly ineffective at suppressing yeast appetite (Figures 2C and 2D), indicating that their degree of mismatch, and not the relative abundance of any one AA, was critical for informing the flies’ perception of protein quality.

Finally, we assayed food consumption when flies were maintained on the same diet as their pretreatment. During a 1 hr period in flyPAD, flies maintained on FLYAA ate significantly less (∼20%) than those maintained on MM1AA (Figure S1). Thus, the net effect of the enhanced phagostimulatory properties and enhanced satiety value of FLYAA was a small reduction in steady-state feeding relative to flies maintained on MM1AA.

### Egg Production Is Quantitatively Predictable Using Exome Matching

Next, we assessed the physiological value of each of the AA ratios for egg production. In previous work, we found that the egg laying of flies feeding on a yeast-based diet is limited by the essential AA (EAA) M (Grandison et al., 2009). If this reflects a stoichiometric limitation, then it should be predictable by the most underrepresented AA in the food when compared with the in silico-translated exome. We focused on the EAAs and conditionally EAAs (see Experimental Procedures) because all others can be acquired by de novo synthesis. More formally, we propose *Drosophila* egg production should be limited by the EAA with value *r*, where

(Equation 1) $$r=\min_{i}\;d_{i}\text{/}p_{i};$$

for EAA *i*, min_i_ is the minimum, and *d*_*i*_ and *p*_*i*_ are the relative concentration in the diet and translated exome, respectively.

**Figure 3:**
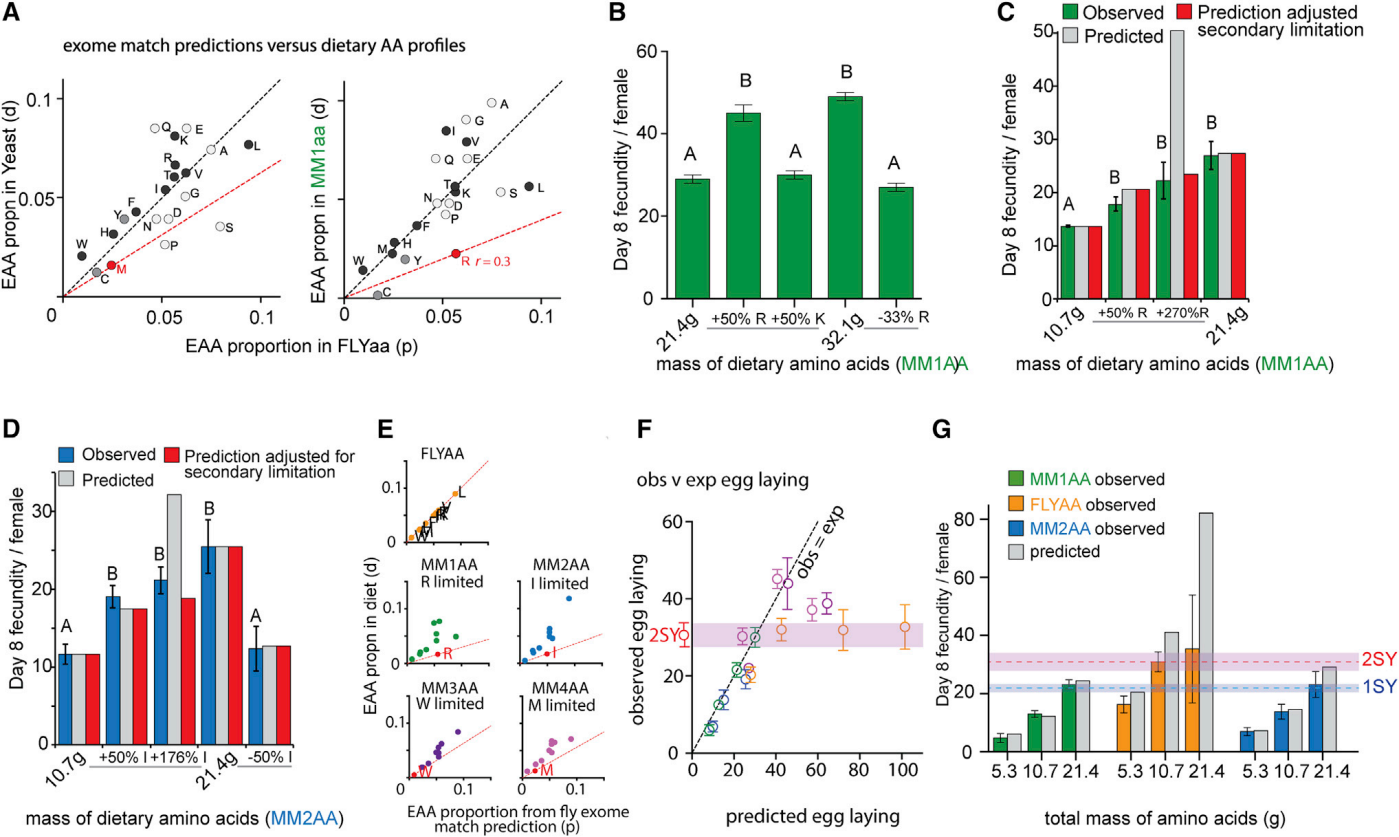
Exome Matching Provides a Quantitative Assessment of Dietary AA Limitations (A) Comparing the relative proportion of dietary essential AAs (EAAs; y axes) to that from in silico exome translation (x axis) reveals the most underrepresented, and thus restricting (*r*), EAA in the diet (red point; M in graph left of panel and R to the right). If diet and translated exome were perfectly matched, all points would lie along the black line with slope = 1. Calculations are based on EAAs (dark gray points) and conditionally EAAs (mid-gray points) because undersupply of C or Y reduces M or F, respectively. For MM1AA, reducing M to supplement C did not surpass the limitation by R. Non-essentials (light gray points) can be generated de novo. (B) Increasing or decreasing R concentration in MM1AA produced a proportionally matched change in egg laying. This was not the case for another EAA, lysine (K) (three trials; different letters represent significant differences, p < 0.05, Wilcoxon rank-sum test; five replicate vials per treatment per trial). (C) Egg laying (green bars) increased in proportion to R addition up to ∼1.7×, but not higher. Gray bars show egg-laying prediction if only constrained by R. Red bars show the prediction from exome matching that M becomes a limiting AA at 1.71× R addition (representative of two trials; ten replicate vials per treatment per trial). (D) Adding isoleucine (I) to MM2AA increased egg laying (blue bars) in agreement with exome-matching prediction. Exome matching (red bars) predicts that W becomes limiting at 1.61× I addition (representative of two trials; ten replicate vials per treatment). (E) Five different dietary AA ratios and their relative AA proportions plotted against the proportions from exome matching. Predicted restricting EAA (*r*) highlighted in red. The slope of the red line through *r* can be used to calculate the egg-laying differences between AA ratios (only EAA shown for clarity). (F) Observed versus exome-matched predictions for egg laying on several concentrations of AA ratios shown in (E) (symbol colors matched between panels). Black diagonal line shows observations = prediction. Egg laying plateaued at intermediate and high levels corresponding to the maximum egg laying attained on concentrated yeast-based food (2SY; average shown by red data point adjacent to y axis ± SE; red shaded area). (G) Observed and expected egg laying for three concentrations of MM1AA, MM2AA, and FLYAA. Egg laying increased similarly with each total AA mass increment, but output was higher on FLYAA than either MM ratio for any given mass of AA (effect of AA ratio on egg laying, p < 0.0001; effect of AA mass, p < 0.0001; p = 0.63 for interaction; generalized linear model). Egg laying plateaued at the level of rich, yeast-based food (2SY; 10.7 g, FLYAA versus 2SY, p = 0.5; 21.4 g, FLYAA versus 2SY, p = 1; Wilcoxon rank-sum test) (2–16 trials per condition; 10 replicate vials per condition per trial). See also Figure S2. All observed egg laying data reported as mean ± SE.

In line with the experimental data, taking the AA content of yeast (Grandison et al., 2009), we thus identified M as the limiting EAA (Figure 3A). Using the same technique for MM1AA, we identified arginine (R), which is essential for flies (Hinton, 1956), as limiting (Figure 3A).

We confirmed this prediction of R limitation for egg laying experimentally. Increasing or reducing R concentration alone in the defined medium caused egg production to increase or decrease to the same extent as when all AAs were altered by the same amount (Figures 3B and S2A) and no other EAA produced these responses. Interestingly, increasing R by half yielded a quantitatively matched proportional increase in egg laying, but with higher levels of supplementation, egg laying only increased by ∼1.7× (Figure 3C). This attenuated response could be explained by exome matching, which quantitatively predicted this limitation by the next most limiting EAA in the diet (M). Furthermore, the same calculations and tests for MM2AA correctly identified isoleucine (I) as limiting and predicted the proportional change in egg laying from I addition until the limit imposed by the next most restrictive AA (tryptophan, W) at 1.61× I addition (Figure 3D). Together, these data indicate that *Drosophila* egg laying is quantitatively governed by a limiting EAA in the diet, which can be identified by exome matching.

Since egg production is constrained by the most limiting EAA in the diet, all others are consumed in excess and must be excreted, in flies largely as uric acid. Thus, the degree of AA mismatch in a diet should be reflected in uric acid production. We measured uric acid produced by flies after exposure to the three AA ratios and found that those consuming MM diets had equal amounts of uric acid, both significantly higher than flies that consumed FLYAA (Figure S2B).

In Equation 1 above, because *r* will predict the limiting EAA for egg laying on the current diet (diet 1), the proportional change in egg laying when flies feed on a diet with a different AA ratio (diet 2) should also be predictable according to *r*_1_/*r*_2_. We found that exome matching predicted egg-laying outcomes for flies on a range of concentrations of five different AA ratios (Figures 3E and 3F). In each case, both the identity of the most limiting AA and the concentration of all predicted non-limiting AAs were varied (Figure 3E), indicating that the most limiting EAA, and no other AA in the mixtures, determined egg-laying output. At intermediate and higher egg-laying rates, the data plateaued at a level that was less than predicted. This level of egg laying at the plateau corresponds to the maximum obtainable on our optimal SY diet (2SY), indicating that at this rate of egg laying, some other factor unrelated to nutritional ratios limits egg output (Figures 3F and 3G). Up to this threshold, exome matching predicts the identity of the most limiting EAA and the extent to which it modifies egg laying.

We found it remarkable that exome information that was unweighted by transcriptome or proteome information could accurately predict egg laying. We therefore assessed the effect of a diet with AA proportions according to a published proteomics-derived measurement of *Drosophila* body composition (PROTEOMEAA; Figure S2C; Sury et al., 2010). Over a range of AA concentrations, the two diets had statistically indistinguishable effects, indicating that within the sensitivity of our assay, accounting for gene expression differences did not improve the accuracy of exome matching for egg laying (Figure S2D).

**Figure 4:**
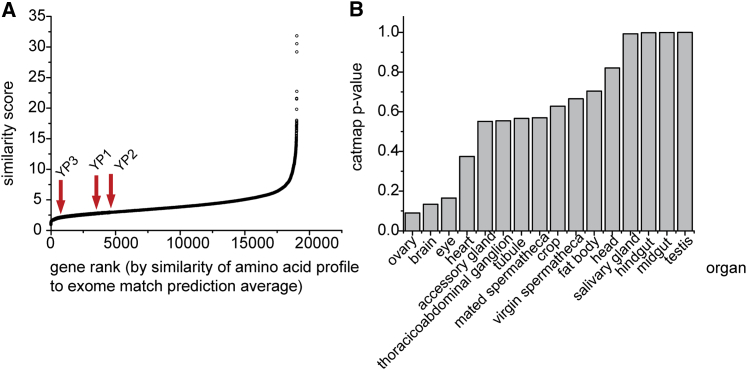
Ovarian-Expressed Genes Use AA in a Pattern Similar to that of the Whole Exome Average (A) In silico-translated genes were ranked (x axis) from least to most similar based on how their AA usage represented that of the whole exome average (y axis). (B) The average rank of ovarian-expressed genes was lower than any other tissue, and was somewhat smaller than expected by chance (p = 0.09, Catmap), indicating the ovary uniquely uses AAs in a manner representative of the exome average.

The failure of proteomics-based prediction to improve on that from exome matching could have several explanations. On the technical side, experimental determination of the proteome is semi-quantitative and can be biased, while on the biological side, measurements based on the whole fly proteome may not detect the specific requirements for egg laying. For instance, exome matching could be accurate because the genes transcribed in the ovary use AAs in a ratio that is peculiarly representative of the whole genome. To assess this, we ranked all in silico-translated genes on the basis of how similar their predicted AA usage is to the average for the whole translated exome (Figure 4A). This revealed that the average ranking of the ovarian-expressed transcripts (Chintapalli et al., 2007) was smaller than expected by chance when compared with randomly selected gene lists of the same size (p = 0.09, Catmap; Breslin et al., 2004; Figure 4B). All other tissue gene sets were less similar to the whole genome. Thus, the ovarian transcriptome may have been constrained toward encoding AAs in the proportions required to maximize future biomass production. This could be particularly important within the confines of an egg, where nutrient and waste exchange with the environment is not possible and thus a balanced AA ratio facilitates development while minimizing the danger of toxicity from accumulated nitrogen catabolites.

### An Exome-Matched Diet Enhances Fly Growth

**Figure 5:**
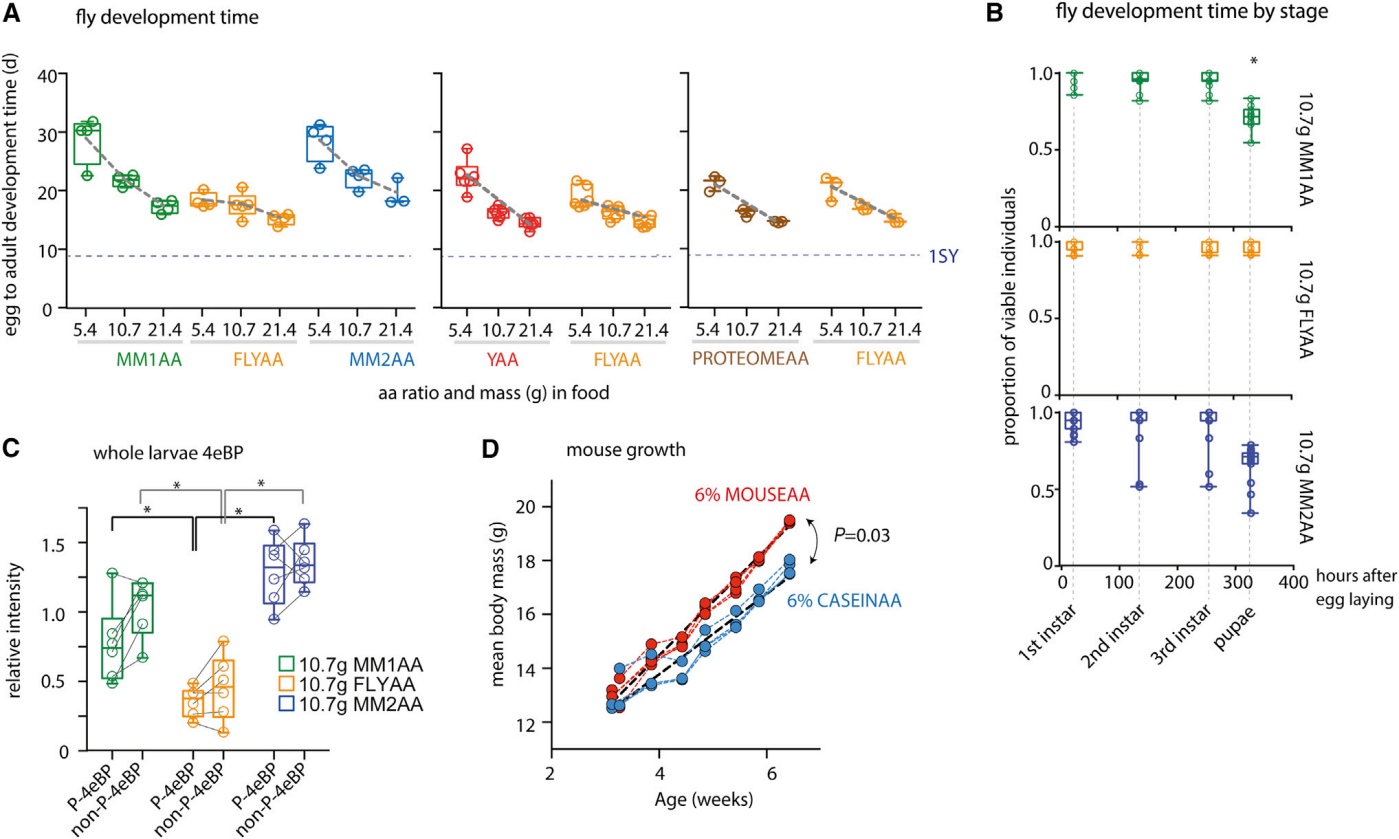
Effect of AA Ratio and Concentration on Development (A) Dilutions of the total mass of AAs for each ratio lengthened fly development time. FLYAA supported quicker development than MM1AA, MM2AA, and YAA in a manner that was less affected by AA dilution (for each comparison in both assays, effect of AA mass, p < 0.001; effect of AA ratio, p < 0.003; mass^∗^AA ratio, p < 0.03; linear model). Using the measured proteome for dietary AA ratio design showed no differences from exome matching (p > 0.4 for effect of AA ratio and interaction with AA mass; linear model; dashed gray lines represent model estimates). Each panel represents a different trial group in which conditions were run concurrently. Each panel shows data from three or more independent trials. (B) Numbers for each developmental stage were scored at the indicated time points and expressed as a proportion of viable individuals in the assay. The proportion at each stage changed over time and with AA ratio (p < 0.001 for effect of time, AA ratio, and their interaction), with an apparent extension of the third instar stage for MM diets (^∗^p < 0.05; data are from three trials with five replicate vials per treatment per trial; each vial contained between 24 and 29 viable individuals; for linear model with mixed effects, vial, nested within trial, was assigned as a random effect). See also Figure S3. (C) Third instar larvae were assessed for phosphorylated and total 4e-BP using western blots. Both forms had significantly lower levels for larvae from FLYAA than from the MM diets (^∗^p < 0.05; for linear model with mixed effects, AA ratio as a fixed effect and replicate blot as random effect; two rounds of gels and blotting of each diet in triplicate were run; 4e-BP bands were normalized to total protein). Corresponding blotted image shown in Figure S3A. (D) Mouse growth rate was significantly enhanced (p < 0.001) by changing a constant mass of AAs from the ratio found in casein (CASEINAA) to that of the translated mouse exome (MOUSEAA). Data collected using five mice in each of four cages per food treatment. Points connected by colored dotted lines represent mass averages per cage. One of two independent trials is shown. Linear mixed effects model: AA ratio, time, and their interaction were treated as fixed effects, while the data from individual mice (nested within cages) and the slope of their mass accumulation were random effects. Heavy black dashed lines show the data fit from the statistical model.

To test if exome matching is also relevant for growth, we reared *Drosophila* larvae on a variety of AA ratios and measured egg to adult development time. Development on the holidic medium is delayed when compared with SY food due to limitation for some unknown factor that is not related to the AA ratio (Piper et al., 2014). It is possible, however, to establish conditions in which growth is AA limited. Within this range, we again found that the exome-matched FLYAA ratio was superior to MM1AA, MM2AA, and a ratio based on the flies’ natural food source, yeast (YAA; Figure 1B) and was again no different from PROTEOMEAA (Figure 5A).

We found that the major delay to development between ratios at 10.7 g total AA mass was caused by lengthening of the third instar phase (Figure 5B). Nutrient-mediated delay in pupariation can be attributed to a combination of reduced systemic insulin/IGF-like signaling and lowered TOR signaling in the fat body and prothoracic gland (Layalle et al., 2008). These combine to reduce growth rate and delay the ecdysone peak that triggers pupariation, thus extending the duration of the third instar stage to allow for some degree of compensatory growth. Indeed, we found that the 4e-BP protein, whose transcription is elevated with decreased IIS (Jünger et al., 2003) and TOR (Bülow et al., 2010), was lower in FLYAA-reared larvae than those on MMAA diets (Figures 5C and S3A), but the proportion of phosphorylated protein was not. We also found no differences in the amount or proportion phosphorylated for S6k (data not shown). The effect on total 4e-BP levels rather than phosphorylation status of either protein indicates a longer-term response to the higher nutritional value of FLYAA. We also found that at low AA concentrations, FLYAA significantly increased the proportion of larvae surviving to adulthood over that in MM1AA, MM2AA, and YAA, as well as the body mass and adult wing size of both adult males and females (Figures S3B–S3E). Thus, FLYAA improved several aspects of development by providing a more nutritious substrate, even improving on a ratio modeled on the flies’ natural food source.

### Exome Matching Improves Dietary AA Ratios for Mouse Growth

In the food industry, there are economic and environmental benefits from improving the efficiency of biomass production (Millward et al., 2008). To assess the potential utility of exome matching for mammals, we searched the literature for examples of rodent diets that had been designed to be AA limiting and for which the identity of the limiting EAA had been verified experimentally. In each instance, exome matching successfully identified the growth-limiting AA: R for rats fed an R-limiting diet (Rogers and Harper, 1965), threonine (T) for rats fed the T-basal diet in Koehnle et al. (2003), L for rats fed the L-basal diet in Hao et al. (2005), and M for mice fed the 0.15% M diet in Miller et al. (2005) (Figure S4).

To test the ability of exome matching to enhance mouse growth under protein-limiting conditions, we designed iso-energetic diets containing a constant mass of purified AAs whose ratio was varied (Figure S5A). To ensure all treatment groups had equal AA intake, meal sizes were standardized across treatments to a mass that was entirely consumed before the next meal. Under these conditions, purified AAs supplied in the proportion found in casein (CASEINAA), the normal source of protein in mouse chow, supported growth just as well as whole protein (Figure S5B). During the initial linear growth phase (weeks 3–6.5), increasing CASEINAA concentration from 6% to 8% (by 33%) caused an ∼40% increase in growth rate (Figure S5C).

**Figure 6:**
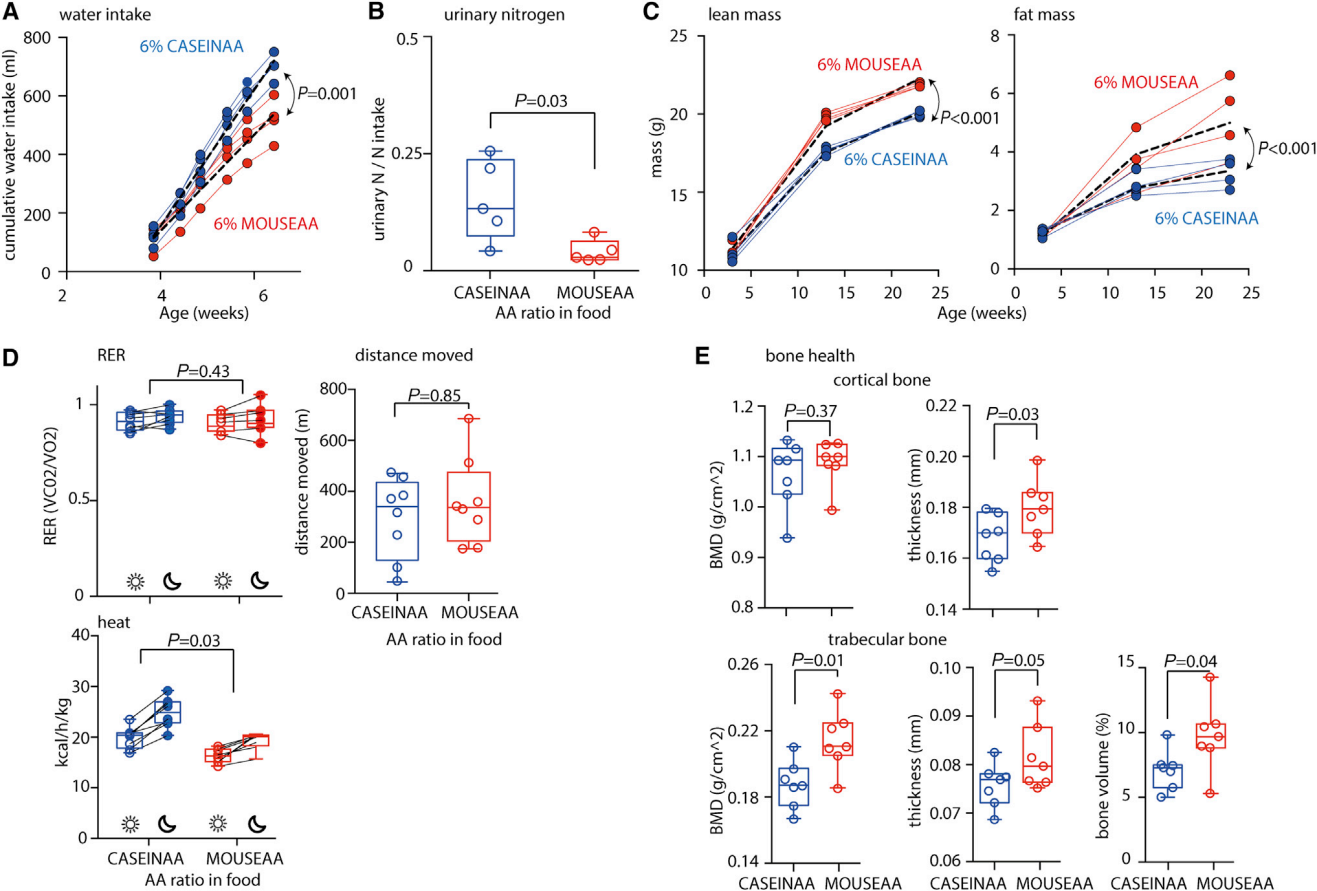
Exome Matching Broadly Alters Mouse Physiology (A) Mice on CASEINAA voluntarily consumed 35% more water than those on MOUSEAA (p = 0.001). Cumulative water consumption per cage shown with heavy black dashed line showing statistical model fit. One of two independent trials is shown. Liner mixed effects model: AA ratio, time, and their interaction as main effects, and cage and its interaction with time were random effects. (B) After 20 weeks of development on the media, MOUSEAA mice excreted a smaller proportion of their ingested nitrogen in urine than those fed CASEINAA (p = 0.03, Wilcoxon rank-sum test). Data show urinary nitrogen excretion from five individual mice. Collected in a single trial. (C) Feeding on MOUSEAA caused a significant increase in the rate of accumulation of both fat (p < 0.001) and lean (p < 0.001) mass during 20 weeks of exposure. Measurements are from each of five mice in four cages (cage averages, colored lines) for both diets. Data gathered from a single trial. Linear model with mixed effects: AA ratio, ln(time), and their interaction were fixed effects, while the data from individual mice (nested within cages) and the slope of their mass accumulation were random effects. (D) At night, RER of all mice was significantly increased (p = 0.01), but there was no effect of diet (p = 0.43, multivariate ANOVA). Total distance moved by mice was not different between diets (p = 0.85, t test). However, there was an increase in thermogenesis of mice on CASEINAA over those on MOUISEAA (p < 0.001), and the increase was more pronounced at night (p = 0.025, diet^∗^time of day interaction, multivariate ANOVA). Data shown are normalized to lean mass; analysis using non-normalized data yielded the same outcomes qualitatively. Data are from eight individuals at 23–24 weeks of age from a single cohort. (E) Mice that developed on MOUSEAA had significantly enhanced femur cortical thickness (p = 0.03), trabecular bone mineral density (BMD) (p = 0.01), and trabecular volume (p = 0.44) when compared with those reared on CASEINAA. t test. Data collected from one trial. Seven animals per condition. See also Figure S6.

When we modified the AA proportion from CASEINAA to that of the translated mouse exome (MOUSEAA), but maintained total protein equivalents at 6%, initial growth rate improved by ∼31% in one trial and ∼33% in a second (Figure 5D; Table S1) and resulted in body mass differences that persisted into adulthood (Figure S6A). At 24 weeks of age, we also found that differences in the free AA profile of the hepatic portal vein for MOUSEAA- versus CASEINAA-fed mice were positively correlated with differences in dietary content (Figure S6B). Although the initial growth rate improvement was somewhat lower than the prediction of 48%, these data indicate that, similar to flies, exome matching enhanced nitrogen source quality for growth. For these same mice, those fed CASEINAA voluntarily consumed ∼35% more water than those fed MOUSEAA (Figure 6A), consistent with a greater proportion of the AAs in CASEINAA being inaccessible for growth and so increasing the water demand for urinary excretion. Indeed, urinary nitrogen excretion of mice that had developed on the diets for 20 weeks (23 weeks of age) was greater for those fed CASEINAA than MOUSEAA (Figure 6B).

The greater body mass of mice developing on MOUSEAA than on CASEINAA was in part attributable to greater lean mass, with both the rate of accumulation and absolute level of lean mass attained being higher (Figure 6C). Fat mass accumulation was similarly increased (Figure 6C). Organs removed from 24-week-old mice revealed a significant increase in mass for white adipose tissue, kidney, liver, and skeletal length, but not for tissues whose size has previously been observed to be refractory to dietary change (Shingleton, 2010), i.e., heart, thymus, quadricep muscle, or brain (Figure S6C). Rearing on CASEINAA and MOUSEAA yielded no differences at 24 weeks in fecal energy content, patterns of movement, or the respiratory exchange ratio (RER), but mice fed CASEINAA showed greater thermogenesis (determined via indirect calorimetry) than did mice fed MOUSEAA (Figures S6D and S6E). Thus, the enhanced energy storage in MOUSEAA animals appeared to be a consequence of reduced energy use for heat production.

Finally, because of their importance for health, we measured several parameters of bone structure in femurs of 23-week-old mice and found that cortical structure thickness, trabecular bone mineral density, and trabecular volume of MOUSEAA-fed animals were significantly greater than those of mice fed CASEINAA (Figure 6E). Thus, MOUSEAA is a higher-quality source of AAs than CASEINAA for both growth and bone structure.

Interestingly, the enhanced quality of the MOUSEAA diet was apparently perceived by the mice since when with ad libitum access to food, young mice consumed ∼15% less food per gram body mass of the 6% MOUSEAA diet than of 6% CASEINAA, and this effect persisted through to adulthood (Figure S7A). Thus, similar to flies, the exome-matched diet was both more efficiently used and resulted in lower steady-state feeding.

**Figure 7:**
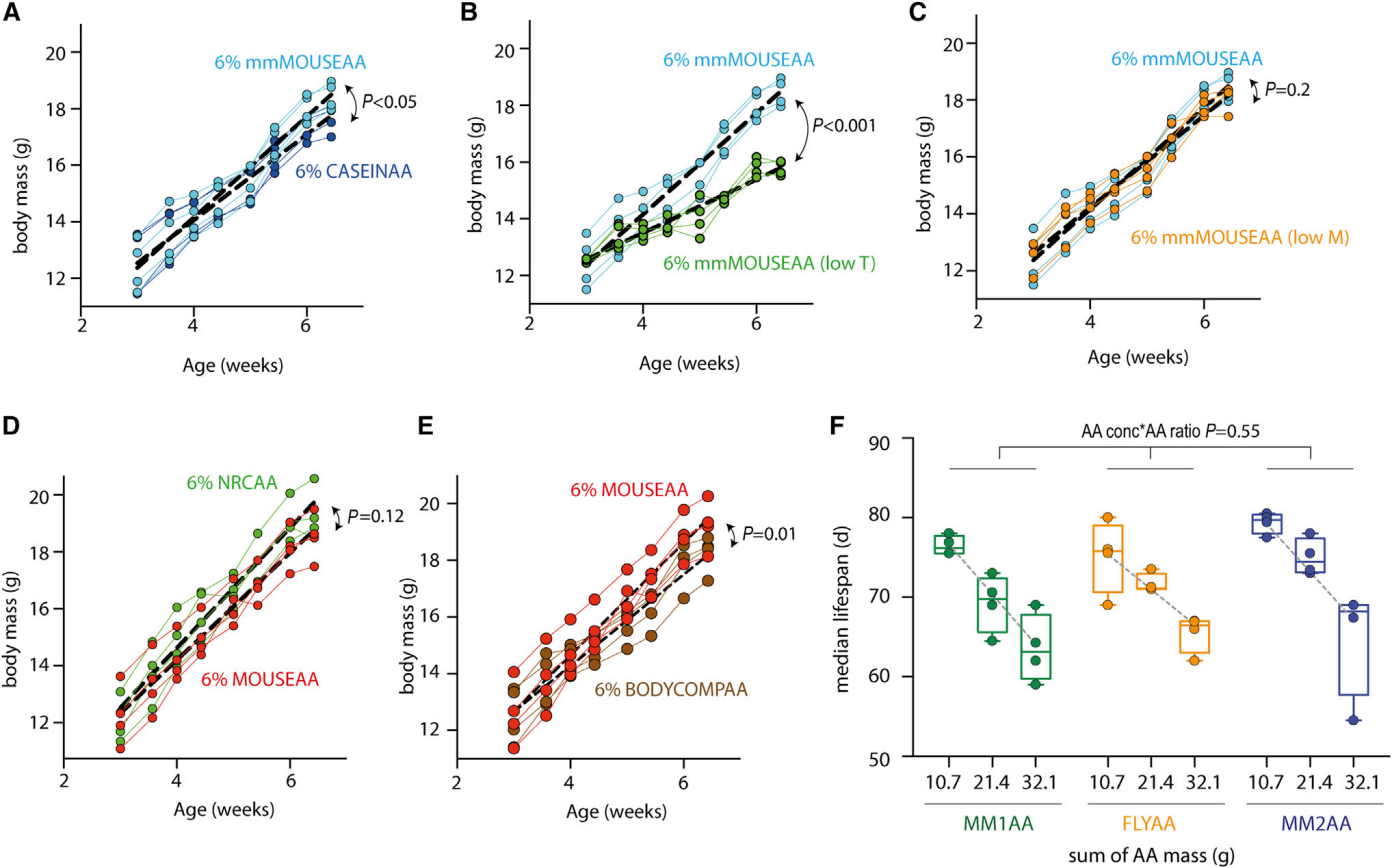
Exome Matching Alters Mouse Development, but Not Fly Lifespan (A) CASEINAA and another MMAA profile (mmMOUSEAA) had similar, but significantly different (p < 0.05), growth rates. (B and C) Reducing the AA (B) predicted to be limiting in mmMOUSEAA (T) reduced growth rate (p < 0.001), but reducing M (C), which was predicted by exome matching to be in excess, did not (p = 0.22). (D) No growth rate difference was detectable for mice feeding on MOUSEAA versus those maintained on NRCAA (p = 0.12). See also Figure S7. (E) Mice fed a diet with AA proportions according to whole-body AA analysis (BODYCOMPAA) had significantly slower growth rate than those on MOUSEAA (p = 0.01). Five mice in each of four cages per nutritional condition. Linear model with mixed effects: AA ratio, time, and their interaction were treated as fixed effects; individual mice nested within cages and the slope of their mass accumulation were random effects. (F) For flies, the relative concentrations of dietary AAs altered median lifespan (p < 0.001), but with no effect of AA ratio, either alone (p = 0.2) or to modify the response to AA concentration (p = 0.55). Linear model with mixed effects: AA concentration and ratio as fixed effects and trial as a random effect. Medians from four trials. A total of 100 flies per condition were used for all trials, except one in which 200 were used.

To further test whether exome matching predicted AA quality for mouse growth, we designed another MMAA ratio (mmMOUSEAA; mismatch MOUSEAA) that differed from both CASEINAA and MOUSEAA in the proportion of AAs, but was predicted to impose a similar growth limitation to CASEINAA. There was little difference in growth rate between mice maintained on CASEINAA or mmMOUSEAA (Figure 7A). Furthermore, exome matching predicted T to be growth limiting in mmMOUSEAA (Figure S7B), and when T was reduced by 30%, growth was reduced (Figure 7B). In contrast, reducing the concentration of M by 30% did not alter growth (Figure 7C), consistent with the exome-matching prediction that it was in 1.4-fold excess (Figure S7B).

Finally, we compared growth of mice fed the exome-matched diet to a cohort fed the recommendations for dietary AAs from the National Research Council (NRC) (National Research Council, 1995) or one matched to a profile based on AA analysis of whole mice (BODYCOMPAA) (Kremen et al., 2013). Both are based on empirical data and are used to inform optimal dietary AA composition. We found no difference in initial growth rate of mice fed MOUSEAA versus those fed NRCAA (Figure 7D), while those assigned the diet based on body composition showed significantly slower growth (Figure 7E). Although not different in its effect during early linear growth, the NRCAA diet did, however, support an ∼8% greater adult body mass (Figure S7C) that whole-body MRI revealed was due to the NRCAA-fed mice gaining fat, but not lean mass, at a faster rate than MOUSEAA-fed mice (Figure S7D). In further tests, we found no differences in movement, fecal energy content, RER, heat production, glucose tolerance, insulin tolerance, or any of the measured bone quality metrics at 23–24 weeks of age (data not shown). Thus, the enriched fat mass of the NRCAA mice may be due to differences in fat biosynthesis.

In summary, exome matching provides an easily implemented framework for establishing a high-quality nitrogen source for mouse growth. We note that its quantitative accuracy in mouse and fly growth was not as precise as for fly egg laying, perhaps due to nutritional buffering by resident microbiota similar to that observed in Schwarzer et al. (2016) and Wong et al. (2014). Nonetheless, our data provide evidence that our completely in silico method can be used for dietary AA design, and that it performs equally as well as, if not better than, natural diets or others that have been developed empirically over decades.

### Low Concentrations of Exome-Matched AAs Avoid the Lifespan/Reproduction Trade-Off

The relative concentration of protein in the diet has been shown to modulate both early life fitness and lifespan, and can account for the benefits of DR in flies and mice (Lee et al., 2008, Mair et al., 2005, Skorupa et al., 2008, Solon-Biet et al., 2014). We therefore assessed the response of *Drosophila* lifespan to varying concentrations of MM1AA, MM2AA, and FLYAA. Consistent with our previous observation (Piper et al., 2014), we found a marked increase in lifespan with decreasing relative concentration of total dietary AAs, an effect that was not modified by AA ratio (Figure 7F), unlike early life where the AA ratio strongly affected growth (Figure 5A) and egg laying (Figures 3E and 3F).

Interestingly, considering the egg laying and lifespan phenotypes together, their response differences caused flies on FLYAA to exhibit a single dietary optimum for growth, reproduction, and lifespan at a relatively low AA concentration (10.7 g/L). In contrast, flies on the MM diets displayed separate optima because egg laying and development required higher levels of AAs (at least 21.4 g/L) than the optimum for lifespan (10.7 g/L). Thus, as we have observed previously (Grandison et al., 2009), changing the dietary AA balance can establish a diet in which the apparent trade-off between reproduction and lifespan is avoided. Here we show that this balance can be established by matching the dietary AA ratio to the in silico-translated exome. In looking for correlated effects on nutrient signaling, we found that total 4e-BP levels, but not the proportion phosphorylated, were reduced in the ovaries of adult flies exposed to 10.7 g/L FLYAA when compared to those on the same concentration of AA in the MM diets (data not shown). In contrast, 4e-BP levels and phosphorylation were unaffected across the same treatments when measured in adult flies from which the ovaries had been removed. Thus, across all conditions we measured, we found that 4e-BP levels changed with growth and reproduction when measured in dividing tissue, and held steady with unchanging lifespan when measured in non-dividing tissue. These data are compatible with an explanation that tissues vary in their sensitivity to AA ratios for modulating IIS and TOR, and that these inter-tissue differences may be key to separating the regulation of lifespan and reproduction.

### Conclusion

Consuming a diet with a relatively low proportion of protein is critical for lifelong health (Le Couteur et al., 2016). However, the costs to early life vigor, reproduction, and low satiety value (Gosby et al., 2011, Solon-Biet et al., 2015, Sørensen et al., 2008) are major detractions. We show that an exome-matched AA composition can reduce voluntary food consumption, and that its enhanced value for growth and reproduction means it can be supplied at low enough levels so as to avoid any cost to lifespan. Given that these are all beneficial outcomes, it will be interesting to determine if there is a physiological cost that we have not yet measured.

It should be noted that the proportions of protein in our experimental diets are particularly low when viewed in the light of an average American diet containing ∼15% protein (Simpson and Raubenheimer, 2005). While exome matching may be useful for screening supplements used to treat protein-energy malnutrition for AA imbalance, it is unlikely to enhance human growth or reproduction in developed countries where protein is generally non-limiting. However, because we have found that exome-matched diets represent high-quality protein that suppresses steady-state feeding at low concentrations, it is possible to envisage that diets to reduce total food intake to enhance long-term health, or even specifically to reduce the proportion of protein in the diet, e.g., for the management of kidney disease (Santesso et al., 2012), could be better achieved using exome matching.

Thus, the principle of exome matching provides a theoretical template for dietary AA design that can enhance the biological efficiency of food for the lifelong health of the consumer.

## Experimental Procedures

Additional details in the Supplemental Experimental Procedures.

### General Fly Handling and Media

Except where indicated, all experiments were conducted with our female *Drosophila melanogaster* (Dahomey). Stocks are maintained outbred and experiments were conducted in controlled conditions: 25°C, 65% humidity, and 12 hr:12 hr light:dark. Except where indicated, flies were reared on 1× SY food at standard density (Bass et al., 2007). Holidic media were made according to Piper et al. (2014) with appropriate substitutions for each of the different AA ratios (Table S2; Data S1).

### Mouse Strain, Housing, and Diets

For each mouse experiment, 20 C3B6F1/J females per treatment were used, housed in four groups of five under specific-pathogen-free (SPF) conditions. Our parental mice were two inbred strains: C57BL/6J and C3H from the Jackson Laboratory.

Except for ad libitum-fed animals, mice were pair fed. Water intake was measured for each cage separately twice per week. Diets were manufactured by Ssniff.

Mouse experiments were performed in accordance with the recommendations and guidelines of the Federation of the European Laboratory Animal Science Association (FELASA), with all protocols approved by the Landesamt für Natur, Umwelt und Verbraucherschutz Nordrhein-Westfalen, Germany.

### Exome Matching

A total of 21,070 proteins were retrieved from FlyBase (FB2008_05, released May 30, 2008). From this, 821 proteins with length less than 100 AAs and 513 with length greater than 2,000 AAs were removed to generate a set of 19,736 non-extreme proteins. This trimming procedure was followed for all animal genomes used. For mouse, Ensemble v 54 (May 2009, downloaded July 2, 2009) was used and for rat, Ensemble v 56 (September 2009, downloaded January 19, 2009). The sum of each AA used in each protein was used to generate its proportional AA usage, and this was combined for all proteins to find the average AA usage for the predicted proteome.

To predict the most limiting EAA in a diet, the proportion of AAs in the food was divided by the proportional representation of AAs in the translated exome of the consumer. The EAA with the lowest value after this transformation was considered limiting. If the requirement for either of the conditionally EAAs Y or C exceeded the available supply, their requirement was met by subtracting a mole of F or M, respectively, for each mole of AA required.

To design MM2AA, we determined the Euclidian distance from MM1AA to FLYAA in 20-dimensional space (1 dimension per AA). We then found another point, MM2AA, that was equidistant from FLYAA as MM1AA, but as far away as possible from MM1AA. This procedure was also performed to generate mmMOUSEAA, using CASEINAA and MOUSEAA as starting points.

### Fly Diet Preference and Feeding Assays

For each trial of each feeding assay, a fresh generation of flies was reared to avoid the possible confounding effects of memory-based decision making.

#### Holidic Diet Choice Assay

A four-arm choice apparatus modified from Cooper (1960) was used. Mated females were AA deprived for 72 hr and acclimatized in the chamber, and their subsequent location was counted hourly for ∼8 hr. The food preference index (FPI) was calculated as (n flies on surface of food A − n flies on surface of food B)/(n flies on surface of food A + n flies on surface of food B). Total AA mass in food was fixed at 21.4 g/L.

#### SY Choice Assay

Groups of 3- to 5-day-old flies (15 females and 5 males) were maintained on yeast-based food or holidic medium. After 72 hr, the flies were tested for nutrient choice as described in Ribeiro and Dickson (2010).

#### flyPAD Monitoring of Feeding Behavior

*w* Dahomey flies were reared in the same medium as for the SY choice assay. Mated adult flies were then maintained on either holidic or yeast-based medium for the pretreatment period (3 days) and assayed using flyPAD, as described in Itskov et al. (2014).

### Measuring Fly Development

First instar larvae were picked onto test media and each developmental stage scored at 25, 138, 258, and 330 hr after egg laying. Adult eclosion was scored daily at 24 hr intervals. Body mass was measured for pairs of newly emerged flies. Wings were measured from the edge of the distal tip to the edge of the alula.

### Fly Uric Acid and TAG Measurements

After 16 hr on holidic medium, flies and medium were removed from the vial, which was washed with 2 mL of 0.1 M sodium glycinate buffer (pH 9.2). Uric acid was quantified spectrophotometrically using the Amplex Red Uric Acid kit (Life Technologies). For TAG determinations, we used the Triglyceride Infinity reagent (Thermo Scientific) and normalized levels to total protein.

### Fly Westerns

The following antibodies were used: 4EBP1 (CST #4923), phospho-4EBP1 (CST #4923), S6K (custom-made, courtesy IHA, UCL), and phospho-S6K (CST #9209).

### Fly Lifespans

Lifespan assays were performed as described in Piper and Partridge (2016). Replicates 1–3 were performed preparing the holidic medium as described in Piper et al. (2014), but for the fourth replicate, the three AAs (I, L, and Y) were added after autoclaving.

### Mouse Physiological Measurements

Body fat content was determined by in vivo magnetic resonance tomography imaging (time domain [TD] NMR).

Indirect calorimetry and movement were monitored over 48 hr for singly housed mice in purpose-built cages (Phenomaster, TSE systems) maintained at 22°C –23°C.

Urea in urine from a 24 hr period was measured using the Urea Assay kit (Sigma) from mice housed in metabolic cages (Tecniplast).

### Portal Vein Plasma Metabolite Analysis

Portal vein blood samples were harvested from pair-fed female C3B6F1/J mice at 23 weeks of age following a 1 g meal and 2 hr food deprivation. Mice were euthanized and dissected, and portal vein blood was collected in EDTA tubes. Analysis was performed by the Finnish Institute for Molecular Medicine (FIMM).

### Mouse Tissue and Bone Measurements

At 23 weeks of age, mice were euthanized using CO_2_ and organs were immediately harvested and weighed. For bone density measurements, right femur bones from 23-week-old mice were collected and scanned with a high-resolution μCT scanner (SkyScan 1176, Bruker). Trabecular and cortical bone regions of distal femurs were selected with reference to the growth plate. Bone mineral density was determined based on calibration with two phantoms of known density (Bruker), which were scanned under the same conditions as the bone samples.

### Statistical Analyses

R (v3.2.0) and JMP (V11) were used for all statistical analyses. Generally, error bars represent SEM.

## Author Contributions

Conceptualization, M.D.W.P. and L.P.; Methodology, M.D.W.P., G.A.S., E.B., I.A., S.J.S., C.R., and L.P.; Formal Analysis, M.D.W.P., G.A.S., E.B., S.L.H., I.A., C.R., and L.P.; Investigation, M.D.W.P., G.A.S., A.M., S.L.H., P.J., X.H., H.S., and M.Y.; Writing – Original Draft, M.D.W.P. and L.P.; Writing – Review & Editing, M.D.W.P., G.A.S., S.J.S., C.R., and L.P.; Funding Acquisition, M.D.W.P., C.R., and L.P.

## Supplemental Information

Document S1. Supplemental Experimental Procedures, Figures S1–S7, and Tables S1 and S2

Data S1. Templates for Fly Holidic Diets and Calculator for Identifying Limiting AA See Experimental Procedures. Tab 1: Using the consumer’s average AA proportion and the AA proportions in the diet, this calculator identifies the EAA predicted to be most limiting in the diet. Text in blue is to be modified by the user; all other cells are calculated automatically. The AA in 1-fold excess in column T is the theoretically most limiting dietary AA. Tab 2: AA proportions used to make the fly diets described in this study. Tab 3: Description of the stock solutions used for each AA ratio described in this study. Tab 4: Recipes used to make the fly holidic media used in this study. Separate diets are itemized for each of the AA ratios and deliver 21.4 g total mass of AA per liter. For details of remaining stock solutions and a more extensive description of the method to make the diet, see Piper et al. (2014).

Document S2. Article plus Supplemental Information
