# Matching Dietary Amino Acid Balance to the In Silico-Translated Exome Optimizes Growth and Reproduction without Cost to Lifespan

**DOI:** 10.1016/j.cmet.2017.04.020

**Published:** 2017-05-02

**Authors:** Matthew D.W. Piper, George A. Soultoukis, Eric Blanc, Andrea Mesaros, Samantha L. Herbert, Paula Juricic, Xiaoli He, Ilian Atanassov, Hanna Salmonowicz, Mingyao Yang, Stephen J. Simpson, Carlos Ribeiro, Linda Partridge

(Cell Metabolism *25*, 610–621; March 7, 2017)

In the originally published version of this article, there were errors in the amino acid ratio values reported in the Supplemental Information accompanying the manuscript. They were as follows: in Table S2, (1) the values for columns under FLYAA were a replica of MM3AA, and (2) the “ratio” columns for MM3AA and MM4AA were based on the mass values rather than the molar values as intended. These same errors were found in Data S1 (tab “Fly_diet aa ratios”). Finally, the molar ratio values for the in silico-translated exome of the “animal” in Data S1 (tab “aa limitations calculator,” column F) did not contain the values for *Drosophila melanogaster* as intended. These errors have now been corrected in the article online. The authors apologize for the errors and any inconvenience that may have resulted.

