# Supplementary material for: Matching Dietary Amino Acid Balance to the In Silico-Translated Exome Optimizes Growth and Reproduction without Cost to Lifespan
Source: Cell Metab. 2017 Mar 7;25(3):610–21. doi: 10.1016/j.cmet.2017.02.005 (PMC5355364; doi:10.1016/j.cmet.2017.02.005)
Supplement: Document S1. Supplemental Experimental Procedures, Figures S1–S7, and Tables S1 and S2 [file mmc1.pdf]

Cell Metabolism, Volume *25*

Supplemental Information

Matching Dietary Amino Acid Balance

to the In Silico-Translated Exome Optimizes

Growth and Reproduction without Cost to Lifespan

Matthew D.W. Piper, George A. Soultoukis, Eric Blanc, Andrea Mesaros, Samantha L. Herbert, Paula Juricic, Xiaoli He, Ilian Atanassov, Hanna Salmonowicz, Mingyao Yang, Stephen J. Simpson, Carlos Ribeiro, and Linda Partridge

Supplemental Figures and Legends

Figure S1

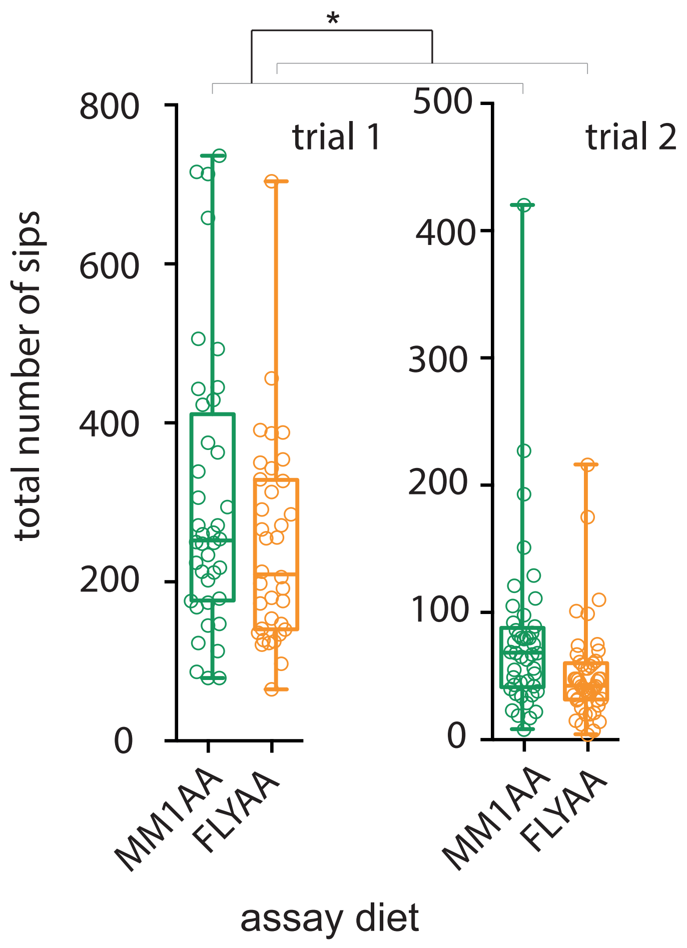

Figure S1. Flies feed slightly less on FLYAA than MM1AA. (See also Figure 2)
Female flies maintained on the indicated food type for 3 days were measured for feeding behaviour using flyPAD (Itskov et al., 2014). Across two experimental blocks, flies were found to take fewer sips when maintained on FLYAA than those maintained on MM1AA (P=0.002). (Linear mixed effects model, AA ratio as fixed effect and experimental block as random effect. In block one, 40 biological replicates for FLYAA, 36 for MM1AA and block 2 there were 45 biological replicates and 48 biological replicates per treatment respectively).

Figure S2

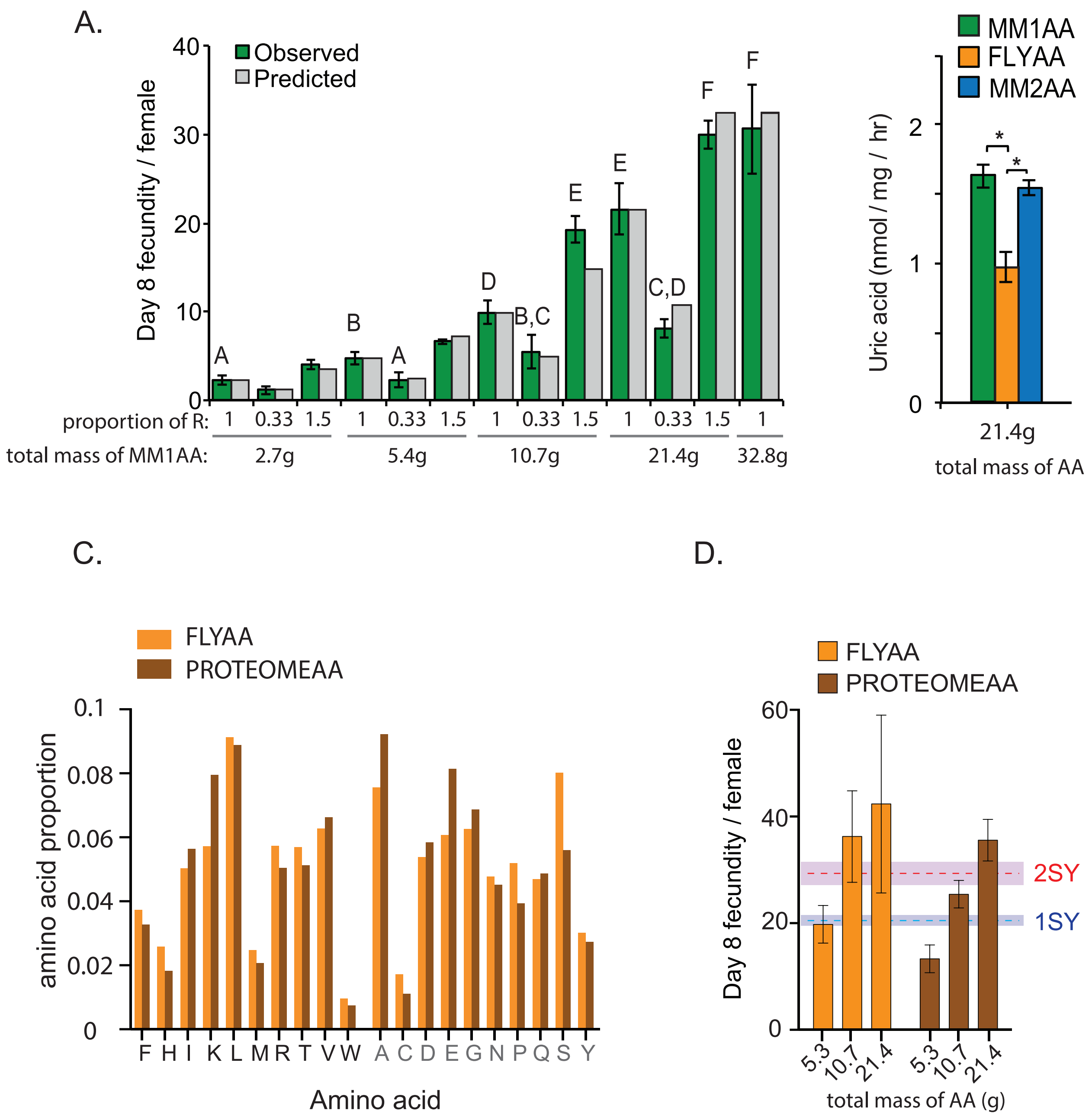

Figure S2. Exome matching predicts both the identity and the degree to which essential amino acids in a complex mixture limit egg laying. (See also Figure 3).

(a) Over a wide range of total amino acid mass additions, altering the concentration of arginine (R) in MM1AA produced a proportionally matched change in egg laying.

(b) Uric acid excretion was measured for flies feeding on media containing each of the three amino acid ratios. During 16 hours, flies on FLYAA produced ~60% of the amount of uric acid as flies feeding on either MM1AA or MM2AA ($P<0.05$, T-test). Data from 4 biological repeats with 10 flies per treatment in each trial.

(d) Egg laying for females maintained on varying concentrations of FLYAA or an amino acid ratio derived from published measured body composition data using whole fly proteomics data (Sury et al., 2010). Comparing egg laying on FLYAA v PROTEOMEAA revealed that only the effect of amino acid mass ($P<0.001$), and not the ratio, or the interaction between the two ($P>0.58$ for both comparisons), had a significant effect on egg laying. (Linear model with mixed effects: Ln (aa mass) and amino acid ratio as fixed effects, modelling the response of egg laying (sqrt transformed). Trial date was set as random effect). N=3. Each replicate employed 6 vials containing 10 flies each. In panels a, b & d, data are presented as mean ± s.e.

Figure S3

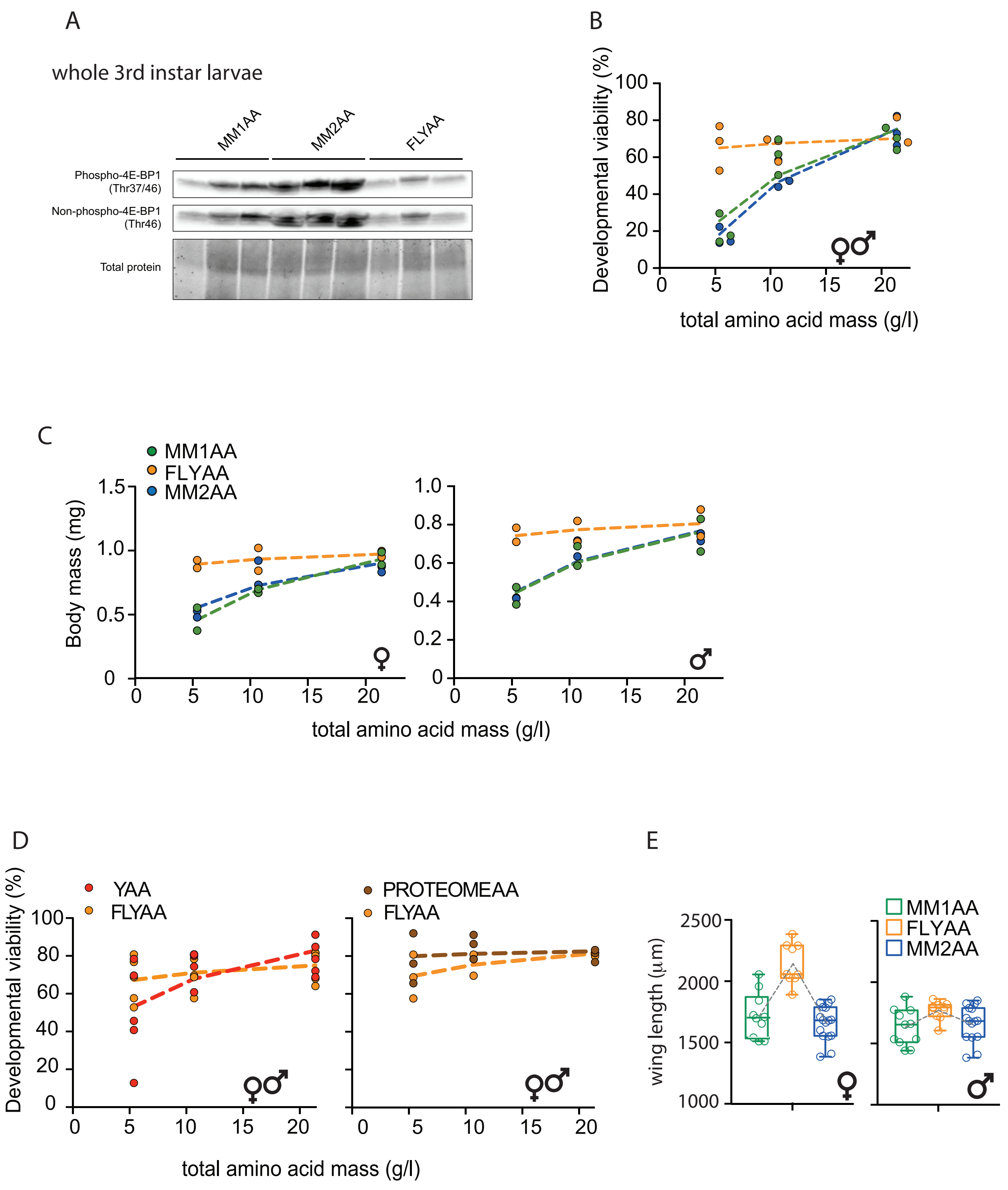

Figure S3. Effect of altering amino acid ratio and mass on developmental timing, viability and adult body mass. (See also Figure 5).

(a) image of Western blot corresponding to quantification in Figure 5c.

(b) Amino acid mass, amino acid ratio and their interaction showed significant effects on viability due to the enhanced viability at the lower concentrations of FLYAA v MM1AA, MM2AA. (*P*<0.0001 for fixed effect; linear

model using log transformed amino acid masses; dashed lines show model estimates). 3 trials were run with 5 vials per food type and 25 eggs per vial.

(c) Body masses of pairs of newly emerged flies were determined for duplicate trials, containing between 2-44 measurements per food, depending on availability of emerged flies. There was a significant effect of trial (P=0.019 for females, P=0.003 for males), amino acid ratio (P<0.001 for females and males) and amino acid mass (P<0.0001 for females and males), however trial date did not modify the other main effects. Amino acid ratio altered the manner in which amino acid mass altered body mass (P=0.006 for females, P=0.005 for males) in such a way that lower amounts of FLYAA supported higher body masses of females and males when compared with the two mismatched ratios. (Linear model using log transformed amino acid masses; dashed lines show model estimates).

(d) There were significant effects of amino acid ratio (P=0.04), amount (P=0.002) and their interaction (P=0.048) for larval viability on YAA v FLYAA. N=5. For PROTEOMEAA v FLYAA, no significant effects were observed. N = 3. In both panels, amino acid ratio and amount (log transformed) and their interaction were fixed effects with trial date assigned as a random effect (Linear mixed effects model).

(e) Wings from 10 day old females and males that had developed on each of the indicated AA ratios (total mass 10.7g/l) were removed and mounted on microscope slides. Length measurements were taken from the distal tip of the wing to the edge of the alula (closest point to the fly).

Figure S4

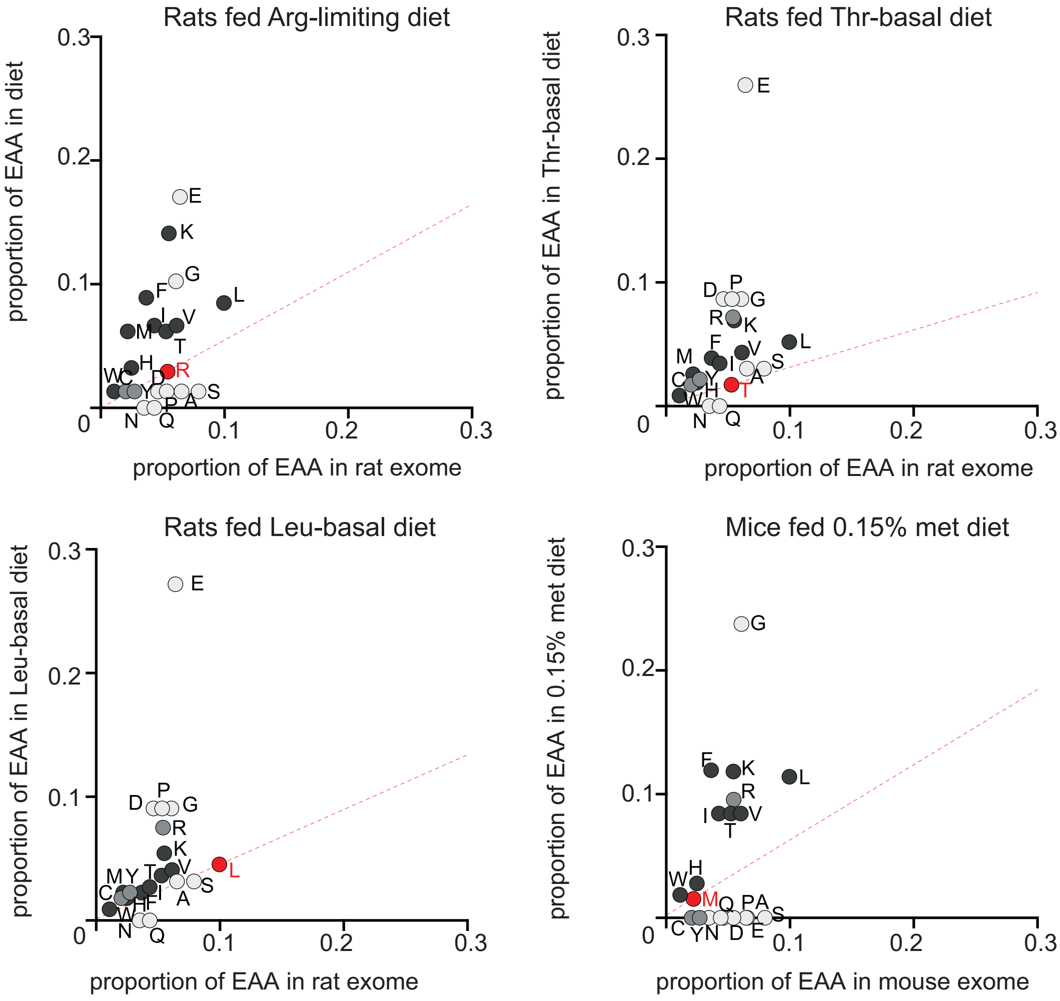

Figure S4. Exome matching correctly predicts the identity of the limiting amino acid (see also Figure 5). In four different studies in which the diet of rodents were fed amino acid imbalanced diets and the most limiting AA confirmed experimentally, exome matching identified the most limiting amino acid (red point). EAAs in dark grey, conditionally essential AAs in mid grey and non-essentials in light grey. Note that for each study, some non-essential AAs were not supplied at all in the diet (points that fall along the x-axis).

Figure S5

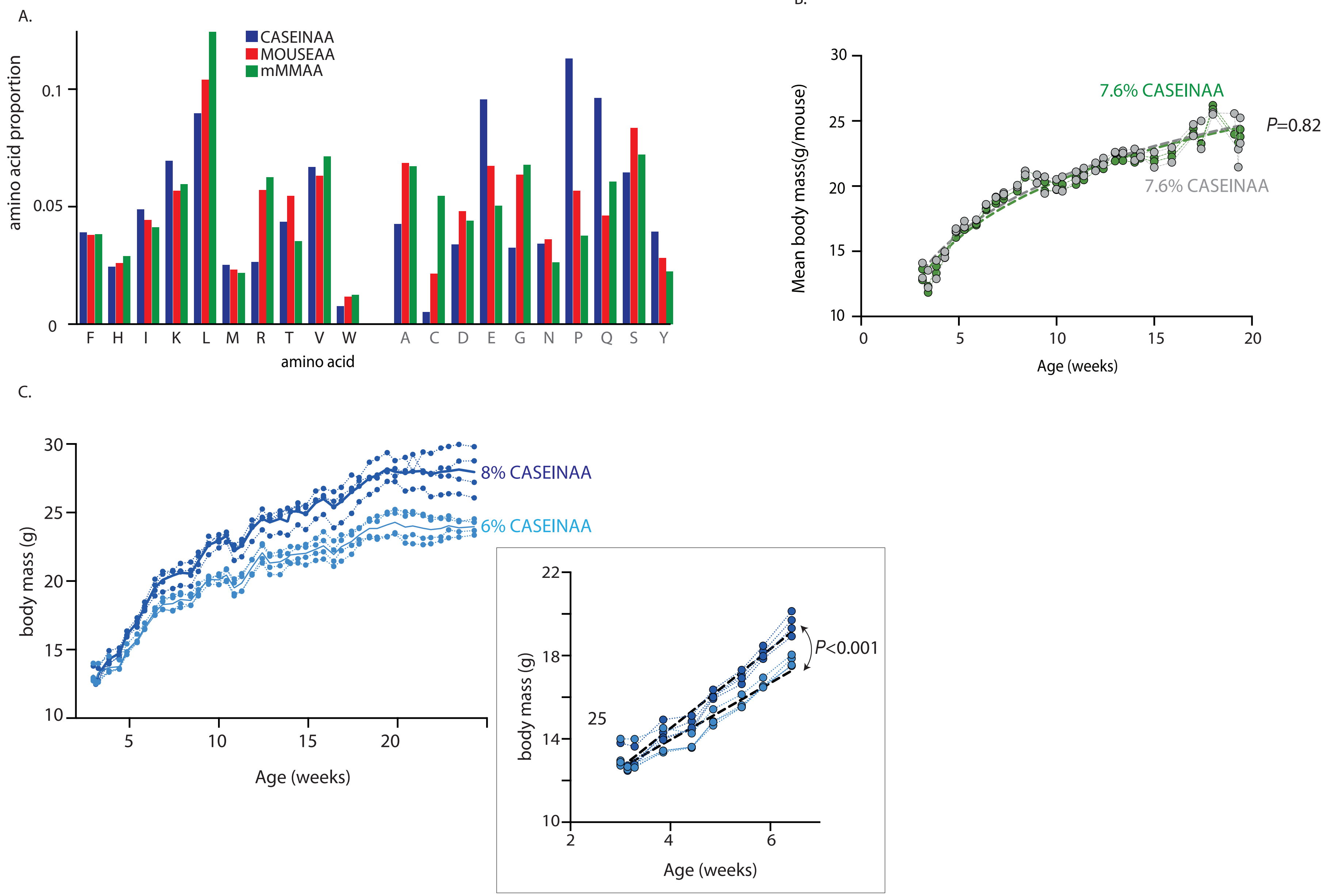
A.
CASEINAA
MOUSEAA
mMMAA
amino acid proportion
0.1
0.05
0
F H I K L M R T V W A C D E G N P Q S Y
amino acid
B.
Mean body mass(g/mouse)
30
25
20
15
10
0 5 10 15 20
Age (weeks)
7.6% CASEINAA
7.6% CASEINAA
P=0.82
C.
body mass (g)
30
25
20
15
10
5 10 15 20
Age (weeks)
8% CASEINAA
6% CASEINAA
body mass (g)
22
18
14
10
2 4 6
Age (weeks)
25
P<0.001

Figure S5. Exome matching and protein restricted mouse growth (see also Figure 5, 6 & 7)
(a) Proportion of amino acids in CASEINAA, MOUSEAA and mouseMMAA
(b) Under protein-limiting conditions (7.5% CP equivalents) diets consisting of purified amino acids or whole protein, as casein, supported growth equally well (P=0.82 for comparing the effect of foods over time; linear model with mixed effects, specifying log time and food type as fixed effects and mice, nested within cages, and their change over time, as random effects). Each of 2 cages (connected circles) per treatment contained 5 mice. Estimates from statistical model plotted as heavy dashed line. Data collected from a single trial.
(c) Casein is the standard protein source for mice in chow. We provided amino acids in growth limiting amounts, such that a 33% increase in total mass (from 6% to 8%) was reflected by a similar, but not proportionally matched (~40%), increase in growth rate. Average mass of mice in each cage plotted as individual points, line average for all mice in each treatment. Inset plot shows only initial linear growth phase (week 3- week 6.5). Individual points connected by dotted lines show cage averages, heavy dashed lines show line fit using parameters from statistical model. (P<0.0001 for slope change, Linear model mixed effects). 5 mice were housed in each of 4 cages per treatment. Data collected from a single trial.

Figure S6

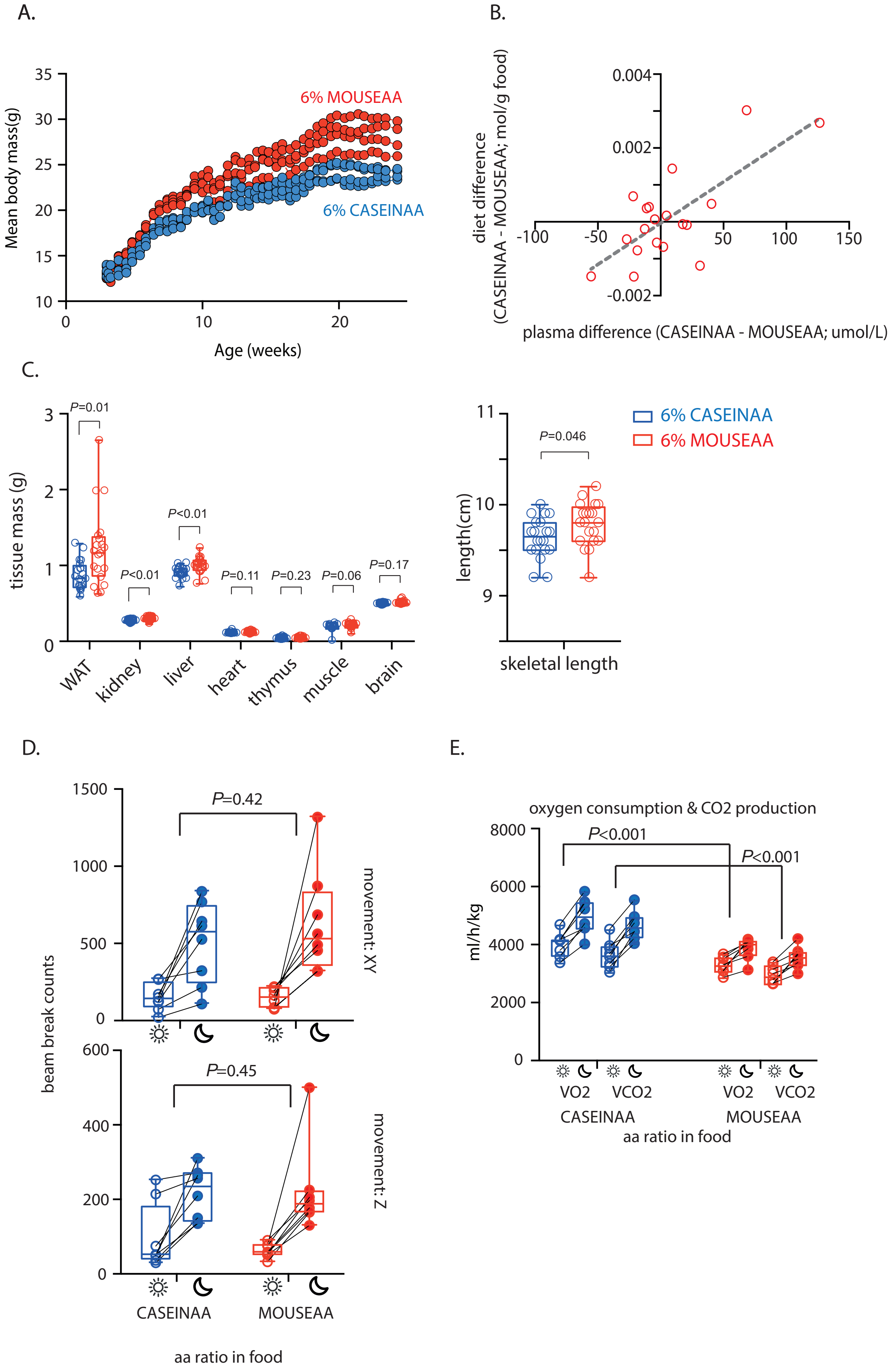

A.
Mean body mass(g)
Age (weeks)
6% MOUSEAA
6% CASEINAA
B.
diet difference (CASEINAA - MOUSEAA; mol/g food)
plasma difference (CASEINAA - MOUSEAA; umol/L)
C.
tissue mass (g)
P=0.01
P<0.01
P<0.01
P=0.11
P=0.23
P=0.06
P=0.17
WAT
kidney
liver
heart
thymus
muscle
brain
length(cm)
P=0.046
skeletal length
6% CASEINAA
6% MOUSEAA
D.
beam break counts
P=0.42
movement: XY
P=0.45
movement: Z
CASEINAA
MOUSEAA
aa ratio in food
E.
oxygen consumption & CO2 production
ml/h/kg
P<0.001
P<0.001
VO2
VCO2
VO2
VCO2
CASEINAA
MOUSEAA
aa ratio in food

Figure S6. Growth and metabolic characterisation of mice developing on CASEINAA versus MOUSEAA (see also Figure 6).

(a) Growth data for mice kept on MOUSEAA and CASEINAA from week 3-24 (continuation of Figure 5d).
(b) Measured differences in the concentration of free amino acids in blood from the hepatic portal vein positively correlated with differences in food (R^2=0.54; P<0.001 non-zero slope). Data collected from 23w old mice that had been maintained on CASEINAA (4 mice) or MOUSEAA (6 mice). Each point in the correlation represents a different amino acid.
(c) Mice were euthanized at 24 weeks and organs removed for weighing. White adipose tissue (WAT), kidney and liver were all significantly heavier in mice from MOUSEAA diet, while no change was detected for the heart, thymus, muscle (right quadriceps) and brain. (Tissues from 20 different individuals for each diet were removed and weighed. T-tests were used to compare tissues between diets where the data was normally distributed, while Wilcoxon rank sum test was used for the remainder).
(d) Movement, measured by counts of light beam breaks in the X, Y and Z planes, revealed no differences in patterns of activity at day or night between CASEINAA and MOUSEAA-fed mice (P>0.42 for effect of food type on activity or food type*time of day interaction, MANOVA).
(e) Rates of O_2 consumption and CO_2 production were significantly higher in CASEINAA mice than MOUSEAA mice (P<0.00). What's more, CASEINAA mice had increased gas exchange elevation at night compared with MOUSEAA mice (P<0.03 when comparing either VO2 or VCO2 diet*time of day interaction, MANOVA). Data in (d & e) are from 8 individuals at 23-24 weeks of age (20-21 weeks of treatment) from a single cohort of mice, each maintained in the Phenomaster for 48h.

Figure S7

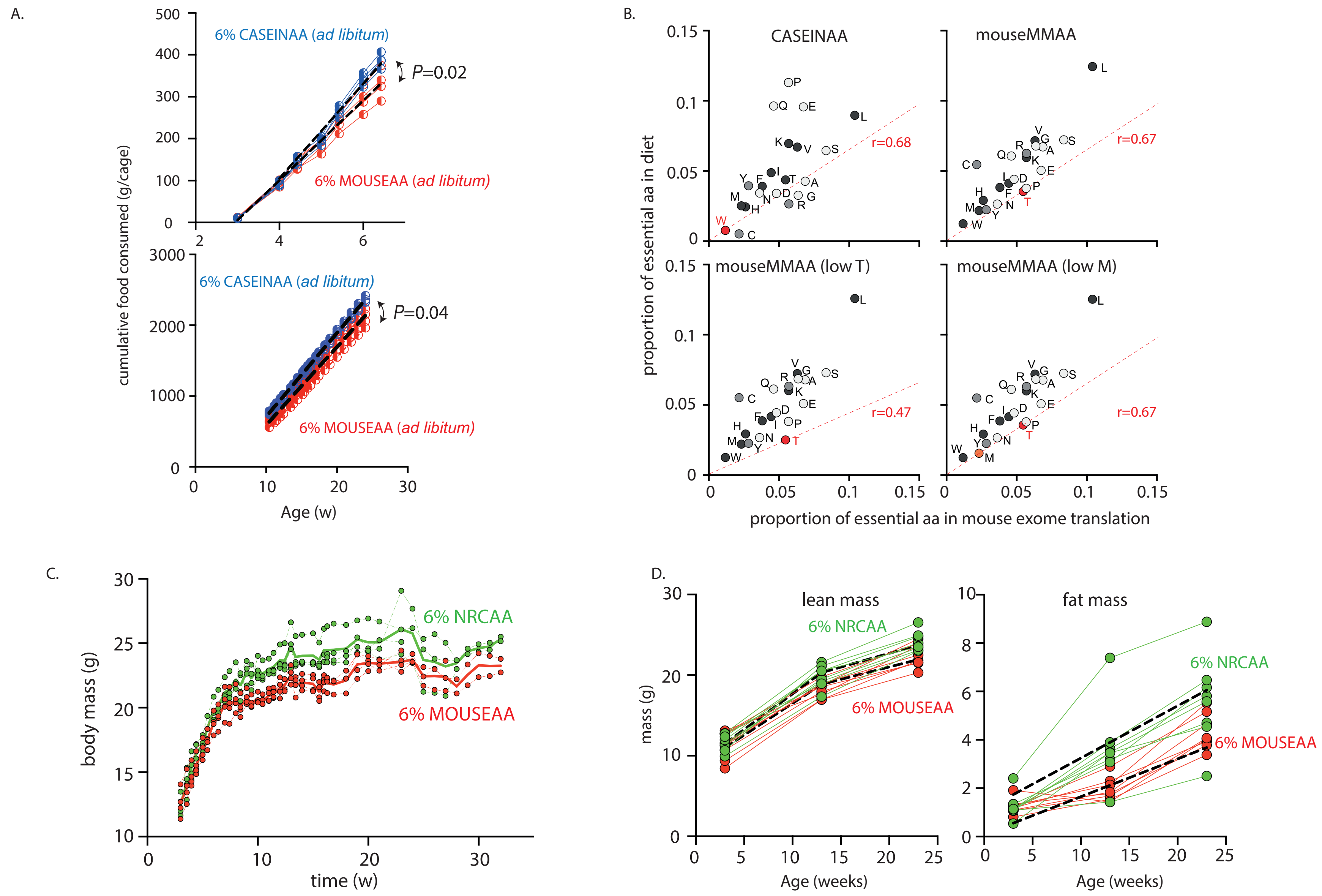

A.
6% CASEINAA (ad libitum)
P=0.02
6% MOUSEAA (ad libitum)
cumulative food consumed (g/cage)
6% CASEINAA (ad libitum)
P=0.04
6% MOUSEAA (ad libitum)
Age (w)
B.
CASEINAA
r=0.68
mouseMMAA
r=0.67
mouseMMAA (low T)
r=0.47
mouseMMAA (low M)
r=0.67
proportion of essential aa in diet
proportion of essential aa in mouse exome translation
C.
6% NRCAA
6% MOUSEAA
body mass (g)
time (w)
D.
lean mass
6% NRCAA
6% MOUSEAA
fat mass
6% NRCAA
6% MOUSEAA
mass (g)
Age (weeks)
Age (weeks)

Figure S7 Mouse growth (see also Figure 7)

(a) Mice with *ad libitum* access to MOUSEAA consumed ~15% less food than those given free access to CASEINAA (P=0.02, Linear model mixed effects), an effect that persisted during adulthood (P=0.04, Linear model mixed effects). Coloured points represent cumulative food consumption per cage, heavy dashed black lines show regression from statistical model. Data from a single trial. AA ratio, time and their interaction were main effects, while the effect of cage and its interaction with time assigned as random effects.
(b)Matching the mouse translated exome to CASEINAA or mmMOUSEAA ratio predicts that tryptophan or threonine (red point) is the limiting amino acid, respectively. Reducing T by 30% in mmMOUSEAA diet exaggerates the T limitation and is thus predicted to further reduce growth rate. However, reducing M by 30% is predicted to produce no growth difference from that of mMMAA since its excess should not be completely depleted (M reduction highlighted by orange point). Thus T should remain the limiting amino acid and growth should be unaffected. In each case the predicted limiting amino acid is highlighted in red and *r* indictaes the relative severity of the under-representation. Differences in *r* between diets are predicted to reveal relative growth rate differences for mice on different diets. Calculations are based on EAAs (dark grey points). Conditionally essential AAs are shown as mid-grey points and non-essentials as light grey points.
(c) Although initial linear growth of mice on NRCAA was indistinguishable from those fed MOUSEAA, the mice maintained on NRCAA accreted and maintained a greater body mass as growth rate slowed.
(d) Whole body MRI revealed the mass differences in panel (b) were largely due to an increased gain of fat mass gain (P=0.03), but not lean mass (P=0.06 for the interaction of how diet affected mass gain over time; linear mixed effects model). Measurements made at week 3, 13 and 23, corresponding to 0, 10 and 20 weeks of treatment. Coloured points represent cumulative tissue mass, heavy dashed black lines show regression from statistical model. Data from a single trial. AA ratio, time (log transformed for lean mass) and their interaction were main effects, while the effect of mouse and its interaction with time assigned as random effects.

Supplemental Tables

Table S1 Reference table for mouse experiments and reported phenotypes. See also Figures 5, 6 & 7.

	Dietary conditions	Growth rates	Water intake	Urinary urea production	Body composition	Energy expenditure and body temperature	Glucose tolerance and insulin tolerance	Bone parameters	Hepatic portal vein blood amino acid profile	Food intake
Expt. A	7.6% CASEINAA (protein; pair-fed)<br><br>7.6% CASEINAA (purified AAs; pair-fed)	Figure S5	Data not shown	Not done	Not done	Not done	Not done	Not done	Not done	Data not shown
Expt. B	6% CASEINAA (*ad libitum*)<br>6% MOUSEAA (*ad libitum*)<br>6% CASEINAA (pair-fed)<br>6% MOUSEAA (pair-fed)<br>8% CASEINAA (pair-fed)	Figure 5b Defined as the linear portion of growth immediately after weaning from week 3 to week 6.4. MOUSEAA improved growth rate by 31% over CASEINAA (pair fed)<br><br>Figure S5d, growth curve continued to end of	Figure 6a Mice fed CASEINAA consumed ~35% more water during the initial growth phase than those on MOUSEAA. Assessing water intake from both experiments together or from this trial alone support this	Not done	Figure 6c The profile of lean and fat mass accumulation was increased for mice fed MOUSEAA over those fed CASEINAA. *In vivo* magnetic resonance tomography imaging was performed on each mouse at weeks 3, 13 and 23	Not done	Not done	Not done	Figure S6b	Data not shown

		measurements at wk 24.	conclusion.		(corresponding to 0, 10 and 20 weeks exposure to diets)					
Expt. C	6% CASEINAA (ad libitum)<br>6% CASEINAA (pair-fed)<br>6% MOUSEAA (pair-fed)<br>6% mmMOUSEAA (pair-fed)<br>6% mmMOUSEAA (low T; pair-fed)<br>6% mmMOUSEAA (low M; pair-fed)<br>6% NRCAA (pair-fed)<br>6% PROTEOMEAA (pair fed)	Data not shown. MOUSEAA improved growth rate by 33% over CASEINAA (pair fed).<br><br>Figure 7 Growth rate effects of mice on mmMOUSEaa.	Data not shown.	Figure 6b Mice fed CASEINAA excreted a greater proportion of their dietary nitrogen intake as urinary urea than those on MOUSEAA. These data collected from mice singly housed in special metabolic cages at the end of the growth experiment (week 23, after mice were exposed to diets for 20 weeks)		Figure 6d Indirect calorimetry and movement were monitored. The only parameter to differ between food types was that CASEINAA mice were observed to generate more heat.<br><br>Data not shown There was no difference in any measured metabolic parameter between NRCAA and MOUSEAA fed animals.	Data not shown<br><br>No differences were detected between MOUSEAA or NRCAA-fed mice in glucose clearance for a glucose or insulin challenge	Figure 6e Bone parameters from right femurs of 23 week old mice.<br><br>Data not shown No differences in any of the bone parameters was detected when comparing mice fed NRCAA versus those on MOUSEAA.	Not done	Figure S6e With free access to food, mice on MOUSEAA voluntarily ate less than those on CASEINAA

Table S2. Amino acid ratios for each of the fly holidic diets. (See Experimental Procedures)

		MM1AA[1]			FLYAA			MM2AA			MM3AA			MM4AA		
		per litre (g)[2]	mM	ratio[3]	per litre (g)	mM	ratio	per litre (g)	mM	ratio	per litre (g)	mM	ratio	per litre (g)	mM	ratio
Essential amino acids																
phenylalanine	F	0.79	4.76	0.027	0.79	4.79	0.028	0.82	4.97	0.030	0.79	4.79	0.037	0.92	5.55	0.043
histidine	H	0.61	3.90	0.022	0.55	3.52	0.020	0.54	3.45	0.021	0.55	3.52	0.026	0.68	4.37	0.032
isoleucine	I	1.82	13.83	0.077	1.11	8.45	0.049	0.40	3.04	0.018	1.11	8.45	0.052	1.16	8.81	0.054
lysine	K	1.15	7.86	0.044	1.21	8.28	0.048	1.26	8.61	0.051	1.21	8.28	0.057	1.74	11.88	0.081
leucine	L	1.21	9.22	0.052	2.01	15.31	0.088	2.72	20.76	0.124	2.01	15.31	0.094	1.64	12.53	0.077
methionine	M	0.48	3.24	0.018	0.52	3.51	0.020	0.61	4.12	0.025	0.52	3.51	0.025	0.34	2.28	0.016
arginine	R	0.48	2.78	0.016	1.21	6.97	0.040	1.93	11.08	0.066	1.21	6.97	0.057	1.42	8.17	0.067
threonine	T	1.21	10.16	0.057	1.21	10.12	0.058	1.19	9.97	0.060	1.21	10.12	0.056	1.29	10.87	0.061
valine	V	1.69	14.47	0.081	1.33	11.35	0.065	0.94	8.03	0.048	1.33	11.35	0.062	1.34	11.43	0.063
tryptophan	W	0.30	1.48	0.008	0.20	1.00	0.006	0.18	0.90	0.005	0.20	1.00	0.010	0.44	2.16	0.021
Non-essential amino acids																
alanine	A	2.12	23.77	0.133	1.60	17.99	0.104	1.04	11.66	0.070	1.60	17.99	0.075	1.59	17.83	0.074
cysteine	C	0.03	0.25	0.001	0.37	3.01	0.017	0.76	6.30	0.038	0.37	3.01	0.017	0.26	2.18	0.012
aspartate	D	1.03	7.73	0.043	1.14	8.58	0.049	1.25	9.38	0.056	1.14	8.58	0.053	0.84	6.31	0.039
glutamate	E	1.51	10.29	0.058	1.34	9.10	0.052	1.14	7.75	0.046	1.34	9.10	0.063	1.82	12.38	0.085
glycine	G	1.94	25.78	0.144	1.32	17.64	0.102	0.69	9.19	0.055	1.32	17.64	0.062	1.08	14.41	0.051
asparagine	N	1.03	7.79	0.044	1.01	7.65	0.044	1.00	7.55	0.045	1.01	7.65	0.047	0.84	6.36	0.039
proline	P	0.91	7.89	0.044	1.10	9.57	0.055	1.29	11.23	0.067	1.10	9.57	0.052	0.56	4.90	0.026
glutamine	Q	1.51	10.35	0.058	0.99	6.80	0.039	0.48	3.30	0.020	0.99	6.80	0.046	1.82	12.45	0.085
serine	S	1.15	10.94	0.061	1.70	16.17	0.093	2.19	20.86	0.125	1.70	16.17	0.079	0.76	7.23	0.036
tyrosine	Y	0.42	2.32	0.013	0.66	3.67	0.021	0.95	5.22	0.031	0.66	3.67	0.031	0.84	4.63	0.039
Sum		21.39		1	21.39		1	21.39		1	21.39		1	21.34		1

[1] Amino acid (AA) ratios: MM(1)AA = mismatch (1); FLY = matched to *in silico* generated exome.
[2] AA masses shown total to 21.4g/l. Other masses used in this study were generated by scaling the same ratio of amino acid masses to achieve the target total mass.
[3] Ratio represents the molar proportions in the media

Supplemental Experimental Procedures

Holidic media for Drosophila
Holidic media were made according to (Piper et al., 2014) with appropriate substitutions for each of the different amino acid ratios (Table S2 and File S1). We found that during storage of the stock solutions of the non-essential amino acids in the ratios for MM2AA and FLYAA, cysteine dropped out of solution. For these media, we therefore omitted C from the combined stock and made a different stock solution that was added separately (see File S1). Three of the amino acids (I,L,Y) are added to the medium before autoclaving for reasons of poor solubility. To address the concern that there may be partial degradation of these amino acids, we compared the effect on development timing and lifespan of adding these before and after autoclaving. We found no evidence for an effect and so where required, data were combined.

Mouse strain, housing and diets
We used two inbred strains from the Jackson laboratory (C57BL/6J and C3H) to generate our C3B6F1/J females used in experiments.
Due to regulatory restrictions on the use of mice, our measurements of different physiological parameters were divided between different cohorts of mice (Experiments B and C outlined in Table S1). For the comparison of MOUSEAA v CASEINAA growth curves, there was no discernible effect of trial (P=0.37) on the influence of diet (P=0.84), time (P=0.72) and their interaction (P=0.88) (Generalised Linear Model, mixed effects). Thus, we have strong evidence that these two cohorts are physiologically comparable.
To enforce pair-feeding, the mass of pellets consumed daily by the group consuming the least amount of food was supplied to the rest of the groups at subsequent time point(s), until cumulative food consumption was equalized between all pair-fed groups. For the *ad-libitum*-fed groups, food intake was measured for each cage separately (mass of pellets on the cage's feeders before and after feeding period).
To ensure mouse growth was protein limited, we empirically determined the crude protein (CP) level for use at 6-8% (Figure S5b). In all cases, diets were isocaloric, with constant micro- and macro-nutrient compositions (except for AA ratios) and with a constant total mass of AAs.

Amino acid usage similarity for protein rankings
We computed the distance between each protein and the average AA content for the in silico translated exome (sum of squared differences of AA ratios according to: sqrt(sum over all AAs of ((ratio in protein - mean ratio in translated exome)/sd ratio in translated exome)^2)). We then ranked all proteins by their distance such that lower rankings are most similar to the exome. The probability of a tissue-specific gene set having a rank-sum significantly higher or lower than that yielded by chance when randomly sampling a similar sized gene set from the ranked list was assessed using Catmap (Breslin et al., 2004).

Amino acid composition determination for flies and mice from published data
To generate the amino acid ratios used in the fly PROTEOMEAA profile, the raw data from a published proteomic study (Sury et al., 2010) were reanalyzed. Protein identification was carried out using MaxQuant (Cox and Mann, 2008) version 1.4.1.2 using the integrated Andromeda search engine (Cox et al., 2011). Raw data were searched against the FlyBase gene translations fasta database (release FB2008_05) containing 21,070 entries. For the protein identification from the raw proteomics data, the fasta database was automatically complemented with sequences of contaminating proteins by MaxQuant. For peptide identification, cysteine carbamidomethylation was set to "fixed" and methionine oxidation and protein N-terminal acetylation as "variable" modifications. For *in silico* digestion the enzyme was set to "LysC/P" allowing for cleavage after lysine, also when followed by proline with a maximum of two missed cleavages. The minimum number of peptides and razor peptides for protein identification was 1; the minimum number of unique peptides was 0. Protein and peptide identification was performed using a false discovery rate (FDR) of 0.01. The "second peptide" option was on allowing for the identification of co-fragmented peptides. In order to transfer identifications to non-sequenced or non-identified peptides in the separate raw files, the option "Match between runs" was turned on using a "Match time window" of 1 min and "Alignment time window" of 20 min. Protein copy numbers per 1g of total protein were derived using the label free quantification based total protein approach (TPA) (Wiśniewski and Rakus, 2014; Wiśniewski et al., 2012). Protein copy numbers were calculated from the protein intensity data from MaxQuant's ProteinGroups.txt output file. As MaxQuant reports

identified proteins in groups when the presence of individual protein sequences cannot be unambiguously inferred, only the first protein ID in a protein group was used. Only proteins that had recorded mass spectrometric intensity more than zero were used for analysis. The combined (heavy and the light SILAC) signal from the proteins was used. Protein copy numbers (per 1g of total protein) were calculated by dividing the protein intensity values by the total intensity of all identified proteins. This value, which corresponds to protein concentration measured in mol per gram total protein, was divided by the protein MW arriving at protein copy numbers. Next, the protein copy number was multiplied by the number of each of the 20 amino acids arriving at the total amino acid numbers for each protein. These latter values were used to calculate the total amino acid quantities in the whole fly and the respective weighted amino acid proportions.

Fly diet preference and feeding assays

Holidic diet choice assay: The apparatus used for the dietary preference assays is a modified version of that described in (Cooper, 1960), which enables the experimental flies to choose between 4 vials containing a medium. We used a prototype scaled-down version of the chamber for 40 once-mated female flies, and tested diet preference, population effects and time-monitoring to determine the experimentally optimal conditions. The chamber lighting was diffuse and the apparatus rotated hourly by 90° to avoid spatial or other environmental cues. Each assay allowed flies the choice of two holidic media differing in their amino acid ratio only, with pairs of foods positioned oppositely in the apparatus. The total mass of amino acids was fixed at 21.4g/l.
Prior to the assay, mated females were maintained for three days on holidic medium without amino acids. Flies were acclimatized in the chamber and allowed time to settle on their diet of choice overnight (approximately 14 hours). The location of flies was counted hourly over ~8 hours and the data for 11am is presented. The effect shown was apparent at each time point measured. The Food Preference Index (FPI) was calculated as: (n flies on surface of food A – n flies on surface of food B) / (n flies on surface of food A + n flies on surface of food B). The DPI was calculated as the average of all data collated for the 8h assay.

Sugar / yeast choice assay: Dahomey flies were reared in yeast-based food containing (per liter of water: 80g sugar cane molasses, 22g sugarbeet syrup, 8g agar, 80g corn flour, 10g soya flour, 18g yeast extract, 8ml propionic acid, 12 ml nipagin (15% in EtOH)). Upon emerging as adults, groups of 3-5 days old flies (15 females and 5 males) were transferred to fresh yeast based food or holidic medium. After 72h, the flies were tested for nutrient choice. Two-choice color feeding preference assays were performed as described in (Ribeiro and Dickson, 2010). Flies were given the choice between sucrose mixed with red colourant (20mM sucrose; 7.5mg/ml agarose; 5mg/ml Erytrosin B (Sigma-Aldrich 198269); 10% PBS) or yeast mixed with blue colourant (10% yeast (SAF instant yeast); 7.5mg/ml agarose; 0.25mg/ml Indigo carmine (Sigma-Aldrich 131164); 10% PBS) medium. After visual inspection of the abdomen, each female fly was scored as having eaten sucrose (red abdomen), yeast (blue abdomen), or both (red and blue or purple abdomen) media. The yeast preference index (YPI) for the whole female population in the assay was calculated as follows: (nblue yeast – nred sucrose)/(nred sucrose + nblue yeast + nboth).

Measuring fly development
Eggs were collected overnight from young, age-matched flies and incubated for 24 h at 25°C, at which point first instar larvae were picked onto test media and returned to 25 °C. The number of larvae of each developmental stage were scored at 17, 21, 25, 90, 114, 138, 258 and 330h after egg laying. Adult eclosion was scored daily at 24-hour intervals. Body mass was measured for pairs of newly emerged flies, and the mean for each pair used for analysis. For wing length, fixed wings were measured from the edge of the distal tip to the edge of the alula using a Leica microscope M165FC, mounted with a DFC420 camera.

Fly uric acid and TAG measurements
10-day old mated females (8 days of treatment) were transferred to fresh vials containing holidic media. After 16 hours, flies and medium were removed from the vial, and the uric acid content of the empty vials dissolved in 2 ml of 0.1 M sodium glycinate buffer (pH 9.2). Uric acid was quantified spectrophotometrically using the Amplex® Red Uric Acid kit (Life Technologies). For TAG determinations, 25 10-day old females per diet were anesthetized using CO2, divided into 5 groups of 5 and homogenized in 1 ml 0.05% Tween 20 and 50ml used for analysis with the Triglyceride Infinity reagent (ThermoScientific). Levels were normalized to total protein.

Mouse physiological measurements
Body fat content was determined by *in vivo* magnetic resonance tomography imaging (time domain (TD) NMR) at weeks 3, 13 and 23 of age (0, 10, and 20 weeks of treatment respectively). The fat content measured by TD-NMR included all types of fat in the body and the lean content included all kind of muscle tissue and free fluid. Typically, the fat, lean and free fluid content amounted to ~92-96% of the total body mass.
Indirect calorimetry and movement were monitored for singly housed mice in purpose built cages (Phenomaster, TSE systems) maintained at 22-23°C. By constantly monitoring the levels of oxygen and carbon dioxide in the incoming and outgoing air, the RER and heat production can be calculated. Laser beam obstruction was used to count incidences of horizontal and vertical movements. Before starting the measurements, mice were housed in training cages in order to acclimatize to single housing, hanging water bottles, special drinking nipples, and hanging food baskets. Mice were intensively checked for their drinking and eating behavior to ensure they accepted the new drinking and feeding system. After the time in the training cages, the mice were acclimatized for 24 hours in the metabolic cages. Data was collected for 48 hours.
Urea in urine was measured in metabolic cages (Tecniplast) that are designed to measure all consumed food and water as well as all excreta. Animals were acclimatised to the cages for 24 hours and measurements made during an additional 24 hours. Urine urea was measured using the Urea Assay Kit (Sigma) and used to calculate the total nitrogen lost in urine. This was expressed as a proportion of dietary nitrogen consumed.
Glucose tolerance tests (GTT) were performed at 23 weeks of age. After an approximately 16 hour fasting period with free access to drinking water, mice were intraperitoneally injected with 2 g of glucose per kg body weight. Blood glucose was measured before, 15, 30, 60 and 120 minutes after glucose injection.
The insulin tolerance test (ITT) was performed at 24 weeks of age. Mice were intraperitoneally injected with 0.75 units of insulin (in 0.9% NaCl) per kg body weight. Blood glucose was measured before, 15, 30 and 60 minutes after insulin injection. ITT mice were fed a small meal of 1 gram per mouse approximately 2 hours prior to being tested.

Portal vein plasma metabolite analysis
Portal vein blood samples were harvested from pair-fed female C3B6F1/J mice at 23 weeks of age following a 1 g meal and 2 hour food deprivation. Mice were euthanized using $CO2$, dissected, and portal vein blood collected in EDTA tubes. These were spun for 15 minutes at 1,200 rpm (4^oC). Samples were then flash-frozen in liquid nitrogen and thawed once for an LC-MS metabolomics analysis performed by the Finnish Institute for Molecular Medicine (FIMM).

Mouse tissue and bone measurements
At 23 weeks of age, mice were euthanized using CO2 and organs were immediately harvested and weighed using a Sartorius ED423S-CS microscale. For bone density measurements, right femur bones from 23 week old mice were collected, placed in 10% PBS saline solution, and stored at -80°C. Before scanning, samples were thawed and hydrated overnight in a saline solution at 5°C. Femurs were scanned with a high resolution µCT scanner (SkyScan 1176, Bruker, Belgium) with an isotropic voxel size of 8.8 μm^3. The x-ray settings for each scan were 50 kV and 500 µA using a 0.5 mm aluminum filter. All scans were performed over 360 degrees with a rotation step of 0.3 degrees and a frame averaging of 1. Images were reconstructed and analyzed using NRecon and CTAn software, respectively (Bruker, Belgium). Trabecular and cortical bone regions of distal femurs were selected with reference to the growth plate (0.44-2.2 and 2.2-2.64 mm from growth plate for group 1, respectively. Size and position of bone regions were adjusted for the other groups depending on femur length). Bone mineral density was determined based on calibration with two phantoms of known density (Bruker, Belgium), which were scanned under the same conditions as the bone samples.

Supplemental File

File S1. Templates for fly holidic diets and calculator for identifying limiting AA. (See Experimental Procedures).
Tab 1: Using the consumer's average AA proportion and the AA proportions in the diet, this calculator identifies the essential AA predicted to be most limiting in the diet. Text in blue is to be modified by the user, all other cells are calculated automatically. The AA in 1-fold excess in column T is the theoretically most limiting dietary AA.

Tab 2: AA proportions used to make the fly diets described in this study
Tab 3: Description of the stock solutions used for each AA ratio described in this study.
Tab 4: Recipes used to make the fly holidic media used in this study. Separate diets are itemised for each of the AA ratios and deliver 21.4g total mass of AA per litre. For details of remaining stock solutions and a more extensive description of the method to make the diet, See (Piper et al, 2014)

Supplemental References

Cooper, D.M. (1960). Food preferences of larval and adult Drosophila. Evolution *14*, 41–55.
Cox, J., and Mann, M. (2008). MaxQuant enables high peptide identification rates, individualized p.p.b.-range mass accuracies and proteome-wide protein quantification. Nat. Biotechnol. *26*, 1367–1372.
Cox, J., Neuhauser, N., Michalski, A., Scheltema, R.A., Olsen, J.V., and Mann, M. (2011). Andromeda: a peptide search engine integrated into the MaxQuant environment. J. Proteome Res. *10*, 1794–1805.
Itskov, P., Moreira, J.-M., Vinnik, E., Lopes, G., Safarik, S., Dickinson, M., and Ribeiro, C. (2014). Automated monitoring and quantitative analysis of feeding behaviour in Drosophila. Nature Communications *5*, 4560.
Piper, M., Blanc, E., Leitão-Gonçalves, R., Yang, M., He, X., Linford, N., Hoddinott, M., Hopfen, C., Soultoukis, G., Niemeyer, C., et al. (2014). A holidic medium for Drosophila melanogaster. Nature Methods *11*, 100–105.
Ribeiro, C., and Dickson, B. (2010). Sex Peptide Receptor and Neuronal TOR/S6K Signaling Modulate Nutrient Balancing in Drosophila. Current Biology *20*, 1000–1005.
Sury, M.D., Chen, J.-X.X., and Selbach, M. (2010). The SILAC fly allows for accurate protein quantification in vivo. Mol. Cell Proteomics *9*, 2173–2183.
Wiśniewski, J.R., and Rakus, D. (2014). Multi-enzyme digestion FASP and the “Total Protein Approach”-based absolute quantification of the Escherichia coli proteome. J Proteomics *109*, 322–331.
Wiśniewski, J.R., Ostasiewicz, P., Duś, K., Zielińska, D.F., Gnad, F., and Mann, M. (2012). Extensive quantitative remodeling of the proteome between normal colon tissue and adenocarcinoma. Mol. Syst. Biol. *8*, 611.
