# Supplementary material for: Matching Dietary Amino Acid Balance to the In Silico-Translated Exome Optimizes Growth and Reproduction without Cost to Lifespan
Source: Cell Metab. 2017 Mar 7;25(3):610–21. doi: 10.1016/j.cmet.2017.02.005 (PMC5355364; doi:10.1016/j.cmet.2017.02.005)
Supplement: Document S2. Article plus Supplemental Information [file mmc3.pdf]

Article

Cell Metabolism

Matching Dietary Amino Acid Balance to the In Silico-Translated Exome Optimizes Growth and Reproduction without Cost to Lifespan

Graphical Abstract

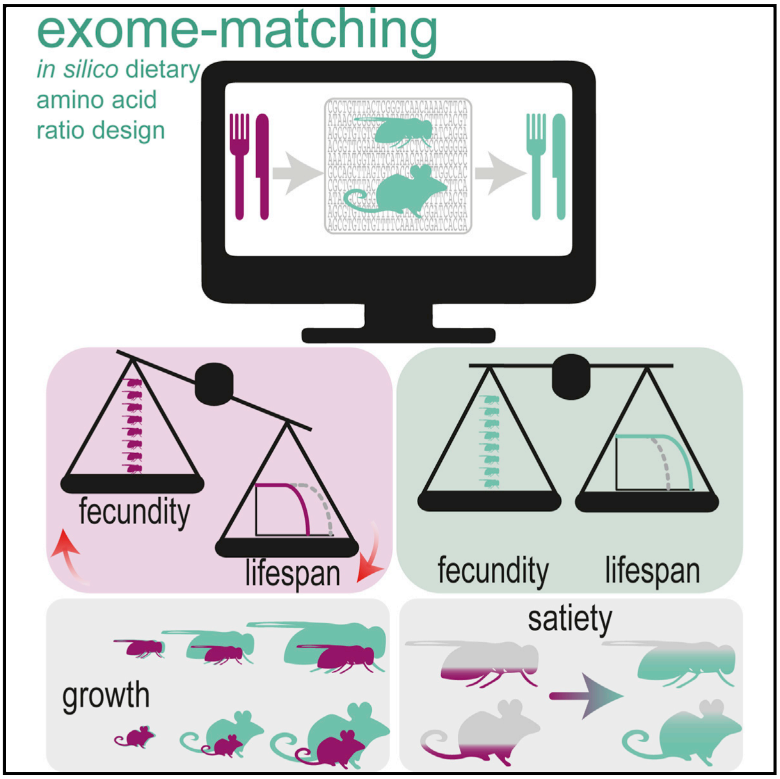

Highlights

- In silico genome translation defines amino acid ratios for exome-matched diets
- Exome-matched diets reduce ad libitum food intake in flies and mice
- Exome-matched diets enhance early life fitness in flies without lifespan cost
- Improved mouse growth on an exome-matched diet demonstrates its utility for mammals

Authors

Matthew D.W. Piper,
George A. Soultoukis, Eric Blanc, ...,
Stephen J. Simpson, Carlos Ribeiro,
Linda Partridge

Correspondence

matthew.piper@monash.edu (M.D.W.P.),
partridge@age.mpg.de (L.P.)

In Brief

Dietary protein is a critical determinant of health, but the empirical determination of optimal amino acid ratios is challenging. Piper et al. show that a consumer's genome provides a template for optimal dietary amino acid proportions. Low amounts of optimized protein are simultaneously beneficial for appetite, growth, reproduction, and lifespan.

Piper et al., 2017, Cell Metabolism *25*, 610–621
March 7, 2017 © 2017 The Author(s). Published by Elsevier Inc.
http://dx.doi.org/10.1016/j.cmet.2017.02.005

CellPress

Cell Metabolism
Article

Matching Dietary Amino Acid Balance to the In Silico-Translated Exome Optimizes Growth and Reproduction without Cost to Lifespan

Matthew D.W. Piper,[1,8,9,*] George A. Soultoukis,[2,8] Eric Blanc,[3] Andrea Mesaros,[2] Samantha L. Herbert,[4] Paula Juricic,[2] Xiaoli He,[5] Ilian Atanassov,[2] Hanna Salmonowicz,[2] Mingyao Yang,[6] Stephen J. Simpson,[7] Carlos Ribeiro,[4] and Linda Partridge[1,2,10,*]
[1]Institute of Healthy Ageing and Department of Genetics, Evolution, and Environment, University College London, London WC1E 6BT, UK
[2]Max Planck Institute for Biology of Ageing, Köln 50931, Germany
[3]Berlin Institute of Health, Berlin 10117, Germany
[4]Behavior and Metabolism Laboratory, Champalimaud Centre for the Unknown, Lisbon 1400-038, Portugal
[5]UCL Ear Institute, University College London, London WC1X 8EE, UK
[6]Institute of Animal Genetics and Breeding, Sichuan Agricultural University, Chengdu 611130, China
[7]Charles Perkins Centre, School of Life and Environmental Sciences, University of Sydney, Sydney 2050, Australia
[8]Co-first author
[9]Present address: School of Biological Sciences, Monash University, Clayton 3800, Australia
[10]Lead Contact
*Correspondence: matthew.piper@monash.edu (M.D.W.P.), partridge@age.mpg.de (L.P.)
http://dx.doi.org/10.1016/j.cmet.2017.02.005

SUMMARY

Balancing the quantity and quality of dietary protein relative to other nutrients is a key determinant of evolutionary fitness. A theoretical framework for defining a balanced diet would both reduce the enormous workload to optimize diets empirically and represent a breakthrough toward tailoring diets to the needs of consumers. Here, we report a simple and powerful in silico technique that uses the genome information of an organism to define its dietary amino acid requirements. We show for the fruit fly *Drosophila melanogaster* that such "exome-matched" diets are more satiating, enhance growth, and increase reproduction relative to non-matched diets. Thus, early life fitness traits can be enhanced at low levels of dietary amino acids that do not impose a cost to lifespan. Exome matching also enhanced mouse growth, indicating that it can be applied to other organisms whose genome sequence is known.

INTRODUCTION

Diets should ideally match the nutritional needs of their consumers for important life history traits such as growth, reproduction, and lifespan. However, quantifying a balanced diet is challenging given the large numbers of nutrients involved. Among the major macronutrients, the proportion of protein is especially important, since relatively high levels that are important to sustain early life fitness can also incur a heavy cost to lifespan (Le Couteur et al., 2016; Soultoukis and Partridge, 2016). Thus, establishing protein balance is critical for understanding how diets can be used to enhance lifelong health.

Many organisms possess mechanisms to prioritize and maintain protein intake to a narrow range of values that are higher than those optimal for longer-term health (Simpson and Raubenheimer, 2012). For example, when protein is relatively low in the diet, total food intake is elevated to maintain protein intake, causing overconsumption of other nutrients (Simpson and Raubenheimer, 2005), a situation thought to contribute to obesity. By contrast, when dietary protein content is high, total food consumption is curbed such that energy may be underconsumed—a formulation effectively exploited for weight loss, but also associated with shortened lifespan in insects, mice (Le Couteur et al., 2016; Lee et al., 2008; Solon-Biet et al., 2014), and humans (Levine et al., 2014). Thus, our evolutionary histories drive consumption of imbalanced foods in a manner that is associated with poor long-term health outcomes.

Investigations into why these trade-offs exist, and how they might be reduced, form an intense area of research into how dietary restriction (DR) extends healthy lifespan (Piper and Bartke, 2008). A long-held idea, derived from life history theory, is that DR improves lifespan by redirecting limiting resources away from reproduction toward somatic maintenance (Kirkwood, 1977; Williams, 1966). However, supplementing the DR diet with methionine (M) in flies can improve reproduction without any cost to lifespan (Dick et al., 2011; Grandison et al., 2009). Thus, enhancing dietary protein quality can increase early life fitness without compromising lifespan. However, understanding how to optimize dietary amino acid (AA) content is not trivial as it represents a 20-dimensional balancing problem. Whereas a theoretical framework is now established (Simpson and Raubenheimer, 2012), a quantitative, evidence-based approach to optimal dietary balance design that does not rely on empirical data has so far proved elusive. The discovery of such a definition for major dietary components would be transformative: diets could be designed to match the requirements of the consumer without the need for lengthy trials. Here we report such a theory for dietary AA balance.

 © 2017 The Author(s). Published by Elsevier Inc.
This is an open access article under the CC BY license (http://creativecommons.org/licenses/by/4.0/).

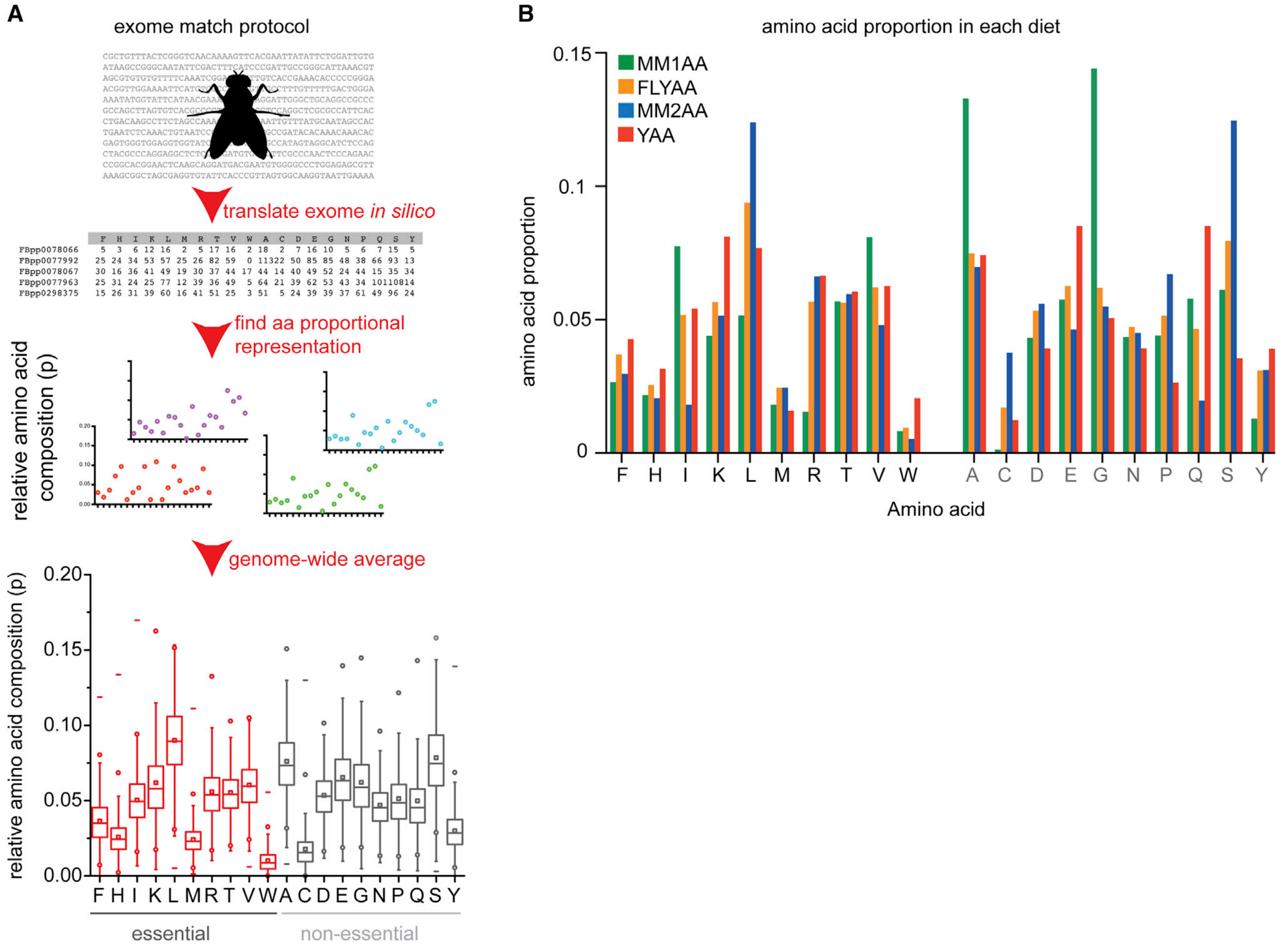

Figure 1. "Exome Matching" to Design Dietary AA Ratios

(A) Computationally, we assembled the proportional representation of each amino acid (AA) in each of *D. melanogaster*'s 19,736 genes. From these, the mean proportional representation of each AA for all genes was determined. This "exome-matched" proportion is that in FLYAA.

(B) Comparison of the relative abundance of each AA profile in this study: MM1AA (mismatch1 AA), FLYAA, MM2AA (mismatch2 AA), and YAA (yeast AA). The ten EAAs are listed first, followed by the non-essentials (gray text).

RESULTS AND DISCUSSION

In Silico Translation of the *Drosophila* Exome to Define Dietary AA Proportions

We hypothesized that the requirement of an animal for each AA is encoded by its genome. Using the *Drosophila melanogaster* genome, we translated in silico its 19,736 predicted protein-coding genes and derived the proportional representation of the 20 AAs (Figure 1A). This fly "exome-matched" AA ratio (FLYAA) was substantially different from an AA proportion previously referred to as HUNTAA during the development of a holidic diet (Hunt, 1970; Piper et al., 2014), referred to here as MM1AA (mismatched1 AA) (Figure 1B).

Exome-Matched Diets Alter Feeding Behavior

We tested if the matched diet (FLYAA) was perceived by flies as preferable to MM1AA. In a two-diet choice assay, in which the foods on offer were identical except that the ratio, but not the total mass, of AAs differed, female flies pretreated on AA-deficient food spent more time on FLYAA than MM1AA (Figure 2A). To assess if this preference was specific to the MM1AA versus FLYAA choice, we designed another ratio (MM2AA) that was equally mismatched to the fly exome translation as MM1AA, but with a different proportion of all 20 AAs (Figure 1B). Again, flies preferred FLYAA over MM2AA (Figure 2A). Interestingly, the flies exhibited no preference for MM1AA or MM2AA. These data indicate that in recovering from AA deficiency, flies that selected a food were not exhibiting indiscriminate food neophilia (Rozin, 1967), but instead specifically detected and chose the matched AA ratio over MM.

To assess the appetitive values of each ratio, we pre-fed all flies on a yeast-based diet and then measured their food intake when restricted to holidic media containing each of the three AA ratios. By pre-feeding a nutritious diet different from any of the test diets, we ensured that subsequent feeding decisions were neither biased by previous experience of the holidic medium, nor redressing a gross nutritional deficit. Using the automated fly feeding monitor flyPAD (Itskov et al., 2014) to track

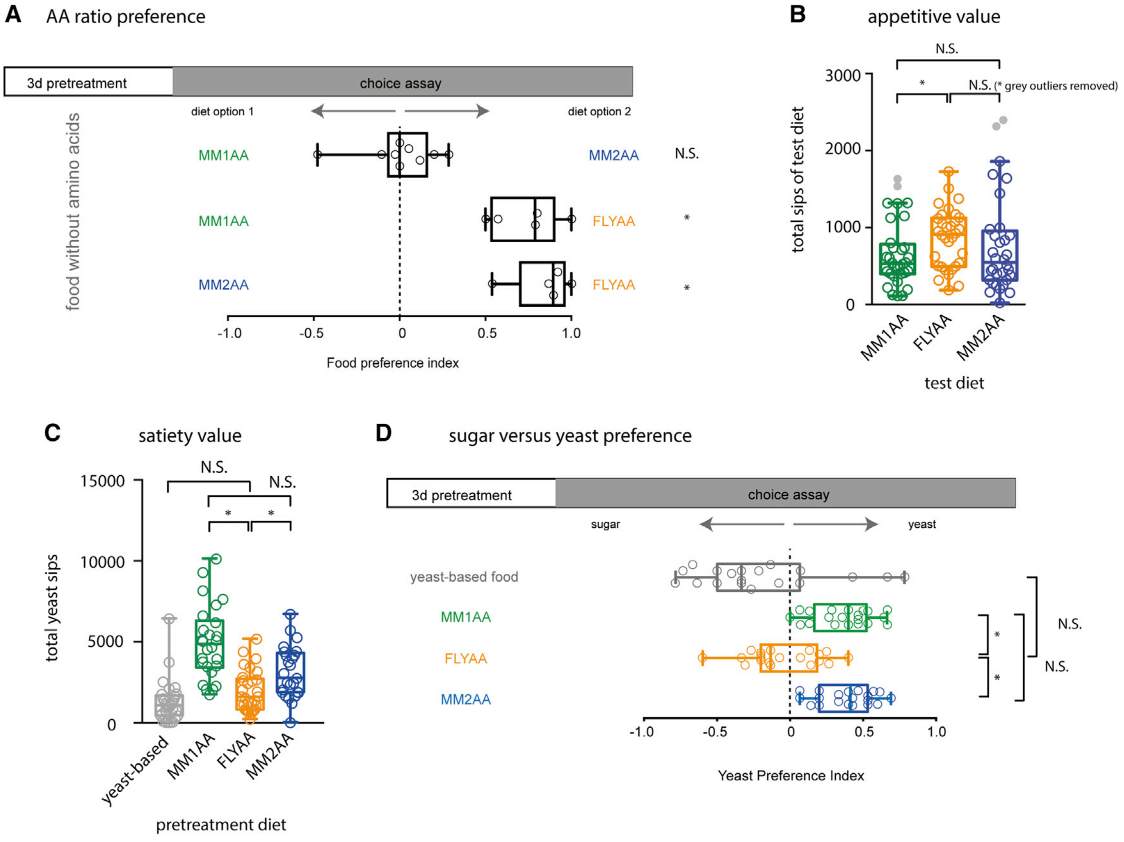

Figure 2. Effect of AA Ratio on *Drosophila* Feeding

(A) Flies pre-starved for AAs were offered two diets differing only in the AA ratio (total mass fixed at 21.4 g/L). Flies preferred the matched ratio (FLYAA) over either MM1AA or MM2AA ($p < 0.03$ in all trials). No preference was observed when the choice was MM1AA versus MM2AA ($p > 0.29$ in 8 trials, $p = 0.02$ in 1 trial) (nine independent trials for MM1AA versus MM2AA, five independent trials each for FLYAA versus MM1AA and FLYAA versus MM2AA; chi-square test; 40 flies per assay).

(B) After pre-feeding on a yeast-based diet, food intake of holidic medium was assessed using flyPAD. Flies on FLYAA ate significantly more ($p = 0.03$) than those on MM1AA, but not MM2AA ($p = 0.06$). After outlier (gray data points) removal, comparison of FLYAA versus MM2AA became significant ($p = 0.02$), while confidence in the difference between FLYAA and MM2AA increased ($p = 0.01$), indicating FLYAA has greater appetitive value than the MM diets (32 individually housed flies monitored per treatment; Wilcoxon rank-sum test and Tukey's test for outlier detection).

(C) Yeast intake was assessed using flyPAD after flies were pretreated on the indicated diets. Flies pretreated with either MM diet ate more than those pretreated with FLYAA or a yeast-based diet ($p < 0.001$ for all comparisons), indicating that FLYAA is more satiating than either MM diet (two independent trials with between 22 and 30 individually monitored flies per food type; linear model with trial and dietary pretreatment as fixed effects).

(D) The preference of flies for yeast (higher yeast preference index, YPI) or sugar (lower YPI) was scored after pretreatment on each of the four diets indicated. FLYAA reduced YPI as effectively as yeast, and more so than either MM diet (20 independent trials for all conditions except 19 for yeast pretreatment; Dunn's test for pairwise comparisons; $*p < 0.05$; N.S., not significant). See also Figure S1.

consumption for 1 hr, flies on FLYAA ate significantly more than those on MM1AA, and also weakly exceeded consumption on MM2AA ($p = 0.06$ down to 0.02 after outlier removal; Figure 2B). MM1AA consumption did not differ from MM2AA. FLYAA thus had a higher appetitive value than either MM ratio. The rapidity with which this behavior appeared shows that decisions about food consumption precede any possible changes in egg laying (see below). Thus, consistent with Corrales-Carvajal et al. (2016) and Walker et al. (2015), the internal nutritional state of the fly sets egg laying and feeding decisions in parallel, rather than in series.

Flies are thought to derive their protein from microbes, in particular yeasts, on decaying fruit (Markow, 2015). We assessed the potency of each of our AA ratios to affect yeast consumption using two different assays that have been used to infer satiety (Itskov et al., 2014; Ribeiro and Dickson, 2010). For both, flies were pre-fed one of the three AA ratios, and their yeast feeding was then assessed in either a no choice (using flyPAD; Figure 2C) or sugar/yeast (SY) choice situation (Ribeiro and Dickson, 2010; Figure 2D). In both assays, flies pre-fed the yeast-based diet or FLYAA ate less yeast than those pre-fed MM1AA or MM2AA (Figures 2C and 2D). Because we measured consumption of a common protein source (yeast) in the assays, these results indicate that yeast consumption specifically responds to AA status. Furthermore, the exome-matched diet was apparently an effective substitute for the natural proportion of AAs found in their ecologically relevant context (i.e., yeast) because pretreatment with FLYAA or a yeast-based diet suppressed yeast intake to the same extent (Figures 2C and 2D). Finally, the two MM diets were similarly ineffective at

suppressing yeast appetite (Figures 2C and 2D), indicating that their degree of mismatch, and not the relative abundance of any one AA, was critical for informing the flies' perception of protein quality.

Finally, we assayed food consumption when flies were maintained on the same diet as their pretreatment. During a 1 hr period in flyPAD, flies maintained on FLYAA ate significantly less (~20%) than those maintained on MM1AA (Figure S1). Thus, the net effect of the enhanced phagostimulatory properties and enhanced satiety value of FLYAA was a small reduction in steady-state feeding relative to flies maintained on MM1AA.

Egg Production Is Quantitatively Predictable Using Exome Matching

Next, we assessed the physiological value of each of the AA ratios for egg production. In previous work, we found that the egg laying of flies feeding on a yeast-based diet is limited by the essential AA (EAA) M (Grandison et al., 2009). If this reflects a stoichiometric limitation, then it should be predictable by the most underrepresented AA in the food when compared with the in silico-translated exome. We focused on the EAAs and conditionally EAAs (see Experimental Procedures) because all others can be acquired by de novo synthesis. More formally, we propose *Drosophila* egg production should be limited by the EAA with value r, where

$$r = \min_i \; d_i / p_i; \qquad \text{(Equation 1)}$$

for EAA i, $\min_i$ is the minimum, and d_i and p_i are the relative concentration in the diet and translated exome, respectively.

In line with the experimental data, taking the AA content of yeast (Grandison et al., 2009), we thus identified M as the limiting EAA (Figure 3A). Using the same technique for MM1AA, we identified arginine (R), which is essential for flies (Hinton, 1956), as limiting (Figure 3A).

We confirmed this prediction of R limitation for egg laying experimentally. Increasing or reducing R concentration alone in the defined medium caused egg production to increase or decrease to the same extent as when all AAs were altered by the same amount (Figures 3B and S2A) and no other EAA produced these responses. Interestingly, increasing R by half yielded a quantitatively matched proportional increase in egg laying, but with higher levels of supplementation, egg laying only increased by ~1.7× (Figure 3C). This attenuated response could be explained by exome matching, which quantitatively predicted this limitation by the next most limiting EAA in the diet (M). Furthermore, the same calculations and tests for MM2AA correctly identified isoleucine (I) as limiting and predicted the proportional change in egg laying from I addition until the limit imposed by the next most restrictive AA (tryptophan, W) at 1.61× I addition (Figure 3D). Together, these data indicate that *Drosophila* egg laying is quantitatively governed by a limiting EAA in the diet, which can be identified by exome matching.

Since egg production is constrained by the most limiting EAA in the diet, all others are consumed in excess and must be excreted, in flies largely as uric acid. Thus, the degree of AA mismatch in a diet should be reflected in uric acid production. We measured uric acid produced by flies after exposure to the three AA ratios and found that those consuming MM diets had equal amounts of uric acid, both significantly higher than flies that consumed FLYAA (Figure S2B).

In Equation 1 above, because r will predict the limiting EAA for egg laying on the current diet (diet 1), the proportional change in egg laying when flies feed on a diet with a different AA ratio (diet 2) should also be predictable according to r_1/r_2. We found that exome matching predicted egg-laying outcomes for flies on a range of concentrations of five different AA ratios (Figures 3E and 3F). In each case, both the identity of the most limiting AA and the concentration of all predicted non-limiting AAs were varied (Figure 3E), indicating that the most limiting EAA, and no other AA in the mixtures, determined egg-laying output. At intermediate and higher egg-laying rates, the data plateaued at a level that was less than predicted. This level of egg laying at the plateau corresponds to the maximum obtainable on our optimal SY diet (2SY), indicating that at this rate of egg laying, some other factor unrelated to nutritional ratios limits egg output (Figures 3F and 3G). Up to this threshold, exome matching predicts the identity of the most limiting EAA and the extent to which it modifies egg laying.

We found it remarkable that exome information that was unweighted by transcriptome or proteome information could accurately predict egg laying. We therefore assessed the effect of a diet with AA proportions according to a published proteomics-derived measurement of *Drosophila* body composition (PROTEOMEAA; Figure S2C; Sury et al., 2010). Over a range of AA concentrations, the two diets had statistically indistinguishable effects, indicating that within the sensitivity of our assay, accounting for gene expression differences did not improve the accuracy of exome matching for egg laying (Figure S2D).

The failure of proteomics-based prediction to improve on that from exome matching could have several explanations. On the technical side, experimental determination of the proteome is semi-quantitative and can be biased, while on the biological side, measurements based on the whole fly proteome may not detect the specific requirements for egg laying. For instance, exome matching could be accurate because the genes transcribed in the ovary use AAs in a ratio that is peculiarly representative of the whole genome. To assess this, we ranked all in silico-translated genes on the basis of how similar their predicted AA usage is to the average for the whole translated exome (Figure 4A). This revealed that the average ranking of the ovarian-expressed transcripts (Chintapalli et al., 2007) was smaller than expected by chance when compared with randomly selected gene lists of the same size (p = 0.09, Catmap; Breslin et al., 2004; Figure 4B). All other tissue gene sets were less similar to the whole genome. Thus, the ovarian transcriptome may have been constrained toward encoding AAs in the proportions required to maximize future biomass production. This could be particularly important within the confines of an egg, where nutrient and waste exchange with the environment is not possible and thus a balanced AA ratio facilitates development while minimizing the danger of toxicity from accumulated nitrogen catabolites.

An Exome-Matched Diet Enhances Fly Growth

To test if exome matching is also relevant for growth, we reared *Drosophila* larvae on a variety of AA ratios and measured egg to adult development time. Development on the holidic medium is

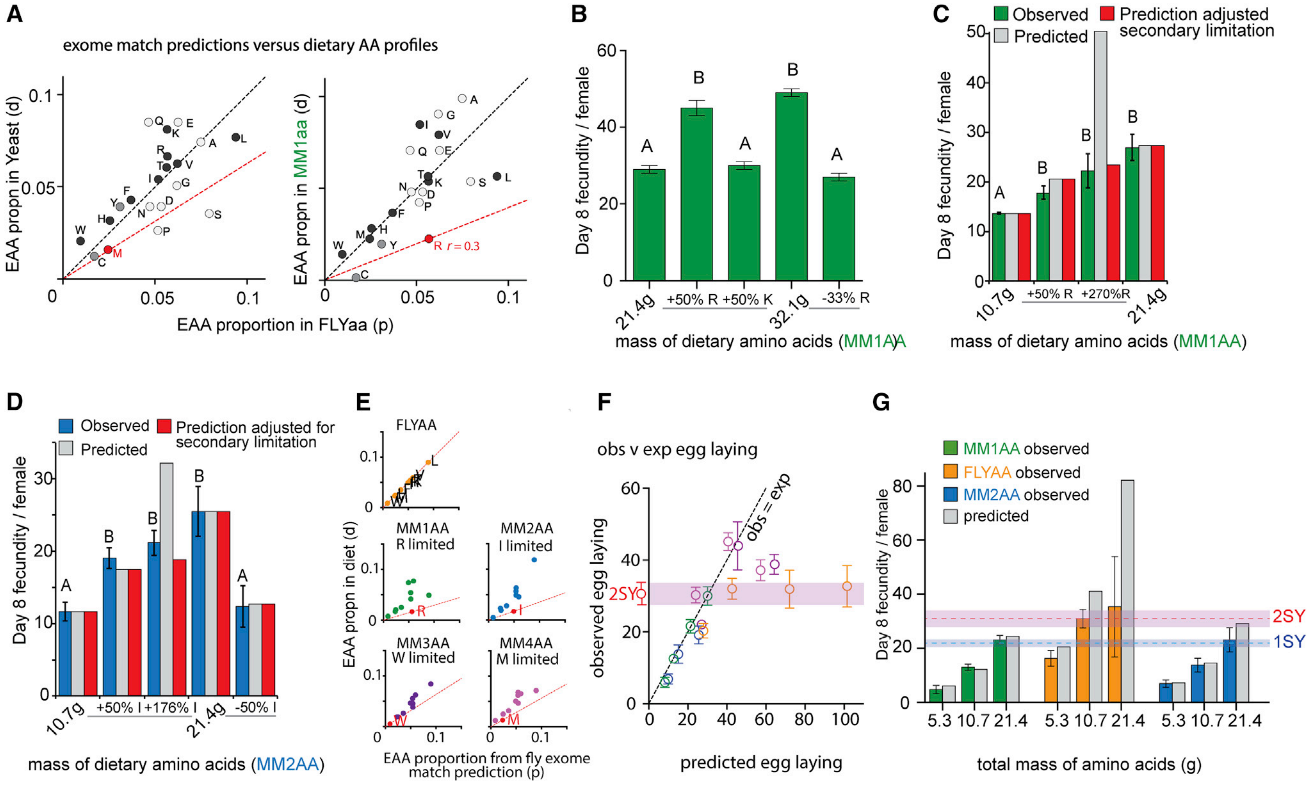

Figure 3. Exome Matching Provides a Quantitative Assessment of Dietary AA Limitations

(A) Comparing the relative proportion of dietary essential AAs (EAAs; y axes) to that from in silico exome translation (x axis) reveals the most underrepresented, and thus restricting (*r*), EAA in the diet (red point; M in graph left of panel and R to the right). If diet and translated exome were perfectly matched, all points would lie along the black line with slope = 1. Calculations are based on EAAs (dark gray points) and conditionally EAAs (mid-gray points) because undersupply of C or Y reduces M or F, respectively. For MM1AA, reducing M to supplement C did not surpass the limitation by R. Non-essentials (light gray points) can be generated de novo.

(B) Increasing or decreasing R concentration in MM1AA produced a proportionally matched change in egg laying. This was not the case for another EAA, lysine (K) (three trials; different letters represent significant differences, $p < 0.05$, Wilcoxon rank-sum test; five replicate vials per treatment per trial).

(C) Egg laying (green bars) increased in proportion to R addition up to ~1.7×, but not higher. Gray bars show egg-laying prediction if only constrained by R. Red bars show the prediction from exome matching that M becomes a limiting AA at 1.71× R addition (representative of two trials; ten replicate vials per treatment per trial).

(D) Adding isoleucine (I) to MM2AA increased egg laying (blue bars) in agreement with exome-matching prediction. Exome matching (red bars) predicts that W becomes limiting at 1.61× I addition (representative of two trials; ten replicate vials per treatment).

(E) Five different dietary AA ratios and their relative AA proportions plotted against the proportions from exome matching. Predicted restricting EAA (*r*) highlighted in red. The slope of the red line through *r* can be used to calculate the egg-laying differences between AA ratios (only EAA shown for clarity).

(F) Observed versus exome-matched predictions for egg laying on several concentrations of AA ratios shown in (E) (symbol colors matched between panels). Black diagonal line shows observations = prediction. Egg laying plateaued at intermediate and high levels corresponding to the maximum egg laying attained on concentrated yeast-based food (2SY; average shown by red data point adjacent to y axis ± SE; red shaded area).

(G) Observed and expected egg laying for three concentrations of MM1AA, MM2AA, and FLYAA. Egg laying increased similarly with each total AA mass increment, but output was higher on FLYAA than either MM ratio for any given mass of AA (effect of AA ratio on egg laying, $p < 0.0001$; effect of AA mass, $p < 0.0001$; $p = 0.63$ for interaction; generalized linear model). Egg laying plateaued at the level of rich, yeast-based food (2SY; 10.7 g, FLYAA versus 2SY, $p = 0.5$; 21.4 g, FLYAA versus 2SY, $p = 1$; Wilcoxon rank-sum test) (2–16 trials per condition; 10 replicate vials per condition per trial). See also Figure S2. All observed egg laying data reported as mean ± SE.

delayed when compared with SY food due to limitation for some unknown factor that is not related to the AA ratio (Piper et al., 2014). It is possible, however, to establish conditions in which growth is AA limited. Within this range, we again found that the exome-matched FLYAA ratio was superior to MM1AA, MM2AA, and a ratio based on the flies' natural food source, yeast (YAA; Figure 1B) and was again no different from PROTEOMEAA (Figure 5A).

We found that the major delay to development between ratios at 10.7 g total AA mass was caused by lengthening of the third instar phase (Figure 5B). Nutrient-mediated delay in pupariation can be attributed to a combination of reduced systemic insulin/IGF-like signaling and lowered TOR signaling in the fat body and prothoracic gland (Layalle et al., 2008). These combine to reduce growth rate and delay the ecdysone peak that triggers pupariation, thus extending the duration of the third instar stage to allow for some degree of compensatory growth. Indeed, we found that the 4e-BP protein, whose transcription is elevated with decreased IIS (Jünger et al., 2003) and TOR (Bülow et al., 2010), was lower in FLYAA-reared larvae than those on MMAA diets (Figures 5C

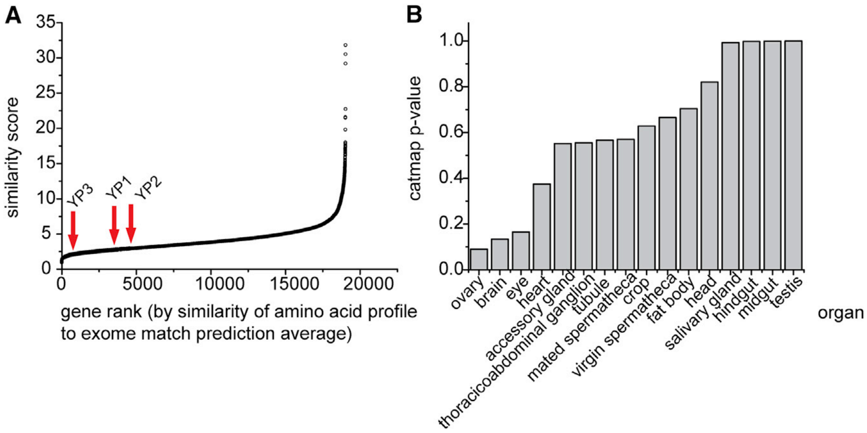

Figure 4. Ovarian-Expressed Genes Use AA in a Pattern Similar to that of the Whole Exome Average

(A) In silico-translated genes were ranked (x axis) from least to most similar based on how their AA usage represented that of the whole exome average (y axis).

(B) The average rank of ovarian-expressed genes was lower than any other tissue, and was somewhat smaller than expected by chance (p = 0.09, Catmap), indicating the ovary uniquely uses AAs in a manner representative of the exome average.

and S3A), but the proportion of phosphorylated protein was not. We also found no differences in the amount or proportion phosphorylated for S6k (data not shown). The effect on total 4e-BP levels rather than phosphorylation status of either protein indicates a longer-term response to the higher nutritional value of FLYAA. We also found that at low AA concentrations, FLYAA significantly increased the proportion of larvae surviving to adulthood over that in MM1AA, MM2AA, and YAA, as well as the body mass and adult wing size of both adult males and females (Figures S3B–S3E). Thus, FLYAA improved several aspects of development by providing a more nutritious substrate, even improving on a ratio modeled on the flies' natural food source.

Exome Matching Improves Dietary AA Ratios for Mouse Growth

In the food industry, there are economic and environmental benefits from improving the efficiency of biomass production (Millward et al., 2008). To assess the potential utility of exome matching for mammals, we searched the literature for examples of rodent diets that had been designed to be AA limiting and for which the identity of the limiting EAA had been verified experimentally. In each instance, exome matching successfully identified the growth-limiting AA: R for rats fed an R-limiting diet (Rogers and Harper, 1965), threonine (T) for rats fed the T-basal diet in Koehnle et al. (2003), L for rats fed the L-basal diet in Hao et al. (2005), and M for mice fed the 0.15% M diet in Miller et al. (2005) (Figure S4).

To test the ability of exome matching to enhance mouse growth under protein-limiting conditions, we designed iso-energetic diets containing a constant mass of purified AAs whose ratio was varied (Figure S5A). To ensure all treatment groups had equal AA intake, meal sizes were standardized across treatments to a mass that was entirely consumed before the next meal. Under these conditions, purified AAs supplied in the proportion found in casein (CASEINAA), the normal source of protein in mouse chow, supported growth just as well as whole protein (Figure S5B). During the initial linear growth phase (weeks 3–6.5), increasing CASEINAA concentration from 6% to 8% (by 33%) caused an ~40% increase in growth rate (Figure S5C).

When we modified the AA proportion from CASEINAA to that of the translated mouse exome (MOUSEAA), but maintained total protein equivalents at 6%, initial growth rate improved by ~31% in one trial and ~33% in a second (Figure 5D; Table S1) and resulted in body mass differences that persisted into adulthood (Figure S6A). At 24 weeks of age, we also found that differences in the free AA profile of the hepatic portal vein for MOUSEAA- versus CASEINAA-fed mice were positively correlated with differences in dietary content (Figure S6B). Although the initial growth rate improvement was somewhat lower than the prediction of 48%, these data indicate that, similar to flies, exome matching enhanced nitrogen source quality for growth. For these same mice, those fed CASEINAA voluntarily consumed ~35% more water than those fed MOUSEAA (Figure 6A), consistent with a greater proportion of the AAs in CASEINAA being inaccessible for growth and so increasing the water demand for urinary excretion. Indeed, urinary nitrogen excretion of mice that had developed on the diets for 20 weeks (23 weeks of age) was greater for those fed CASEINAA than MOUSEAA (Figure 6B).

The greater body mass of mice developing on MOUSEAA than on CASEINAA was in part attributable to greater lean mass, with both the rate of accumulation and absolute level of lean mass attained being higher (Figure 6C). Fat mass accumulation was similarly increased (Figure 6C). Organs removed from 24-week-old mice revealed a significant increase in mass for white adipose tissue, kidney, liver, and skeletal length, but not for tissues whose size has previously been observed to be refractory to dietary change (Shingleton, 2010), i.e., heart, thymus, quadricep muscle, or brain (Figure S6C). Rearing on CASEINAA and MOUSEAA yielded no differences at 24 weeks in fecal energy content, patterns of movement, or the respiratory exchange ratio (RER), but mice fed CASEINAA showed greater thermogenesis (determined via indirect calorimetry) than did mice fed MOUSEAA (Figures S6D and S6E). Thus, the enhanced energy storage in MOUSEAA animals appeared to be a consequence of reduced energy use for heat production.

Finally, because of their importance for health, we measured several parameters of bone structure in femurs of 23-week-old mice and found that cortical structure thickness, trabecular bone mineral density, and trabecular volume of MOUSEAA-fed animals were significantly greater than those of mice fed CASEINAA (Figure 6E). Thus, MOUSEAA is a higher-quality source of AAs than CASEINAA for both growth and bone structure.

Interestingly, the enhanced quality of the MOUSEAA diet was apparently perceived by the mice since when with ad libitum access to food, young mice consumed ~15% less food per gram

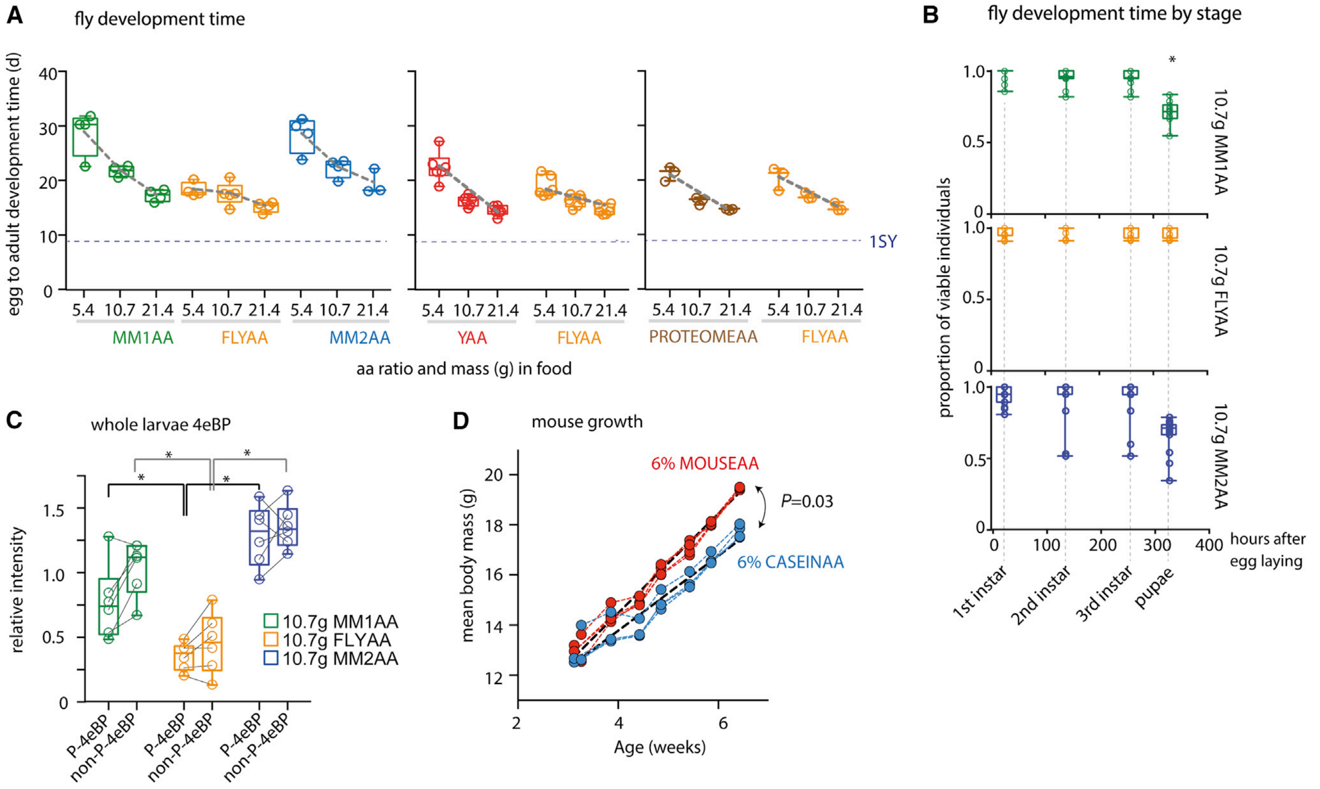

Figure 5. Effect of AA Ratio and Concentration on Development

(A) Dilutions of the total mass of AAs for each ratio lengthened fly development time. FLYAA supported quicker development than MM1AA, MM2AA, and YAA in a manner that was less affected by AA dilution (for each comparison in both assays, effect of AA mass, $p < 0.001$; effect of AA ratio, $p < 0.003$; mass*AA ratio, $p < 0.03$; linear model). Using the measured proteome for dietary AA ratio design showed no differences from exome matching ($p > 0.4$ for effect of AA ratio and interaction with AA mass; linear model; dashed gray lines represent model estimates). Each panel represents a different trial group in which conditions were run concurrently. Each panel shows data from three or more independent trials.

(B) Numbers for each developmental stage were scored at the indicated time points and expressed as a proportion of viable individuals in the assay. The proportion at each stage changed over time and with AA ratio ($p < 0.001$ for effect of time, AA ratio, and their interaction), with an apparent extension of the third instar stage for MM diets (*$p < 0.05$; data are from three trials with five replicate vials per treatment per trial; each vial contained between 24 and 29 viable individuals; for linear model with mixed effects, vial, nested within trial, was assigned as a random effect). See also Figure S3.

(C) Third instar larvae were assessed for phosphorylated and total 4e-BP using western blots. Both forms had significantly lower levels for larvae from FLYAA than from the MM diets (*$p < 0.05$; for linear model with mixed effects, AA ratio as a fixed effect and replicate blot as random effect; two rounds of gels and blotting of each diet in triplicate were run; 4e-BP bands were normalized to total protein). Corresponding blotted image shown in Figure S3A.

(D) Mouse growth rate was significantly enhanced ($p < 0.001$) by changing a constant mass of AAs from the ratio found in casein (CASEINAA) to that of the translated mouse exome (MOUSEAA). Data collected using five mice in each of four cages per food treatment. Points connected by colored dotted lines represent mass averages per cage. One of two independent trials is shown. Linear mixed effects model: AA ratio, time, and their interaction were treated as fixed effects, while the data from individual mice (nested within cages) and the slope of their mass accumulation were random effects. Heavy black dashed lines show the data fit from the statistical model.

body mass of the 6% MOUSEAA diet than of 6% CASEINAA, and this effect persisted through to adulthood (Figure S7A). Thus, similar to flies, the exome-matched diet was both more efficiently used and resulted in lower steady-state feeding.

To further test whether exome matching predicted AA quality for mouse growth, we designed another MMAA ratio (mmMOUSEAA; mismatch MOUSEAA) that differed from both CASEINAA and MOUSEAA in the proportion of AAs, but was predicted to impose a similar growth limitation to CASEINAA. There was little difference in growth rate between mice maintained on CASEINAA or mmMOUSEAA (Figure 7A). Furthermore, exome matching predicted T to be growth limiting in mmMOUSEAA (Figure S7B), and when T was reduced by 30%, growth was reduced (Figure 7B). In contrast, reducing the concentration of M by 30% did not alter growth (Figure 7C), consistent with the exome-matching prediction that it was in 1.4-fold excess (Figure S7B).

Finally, we compared growth of mice fed the exome-matched diet to a cohort fed the recommendations for dietary AAs from the National Research Council (NRC) (National Research Council, 1995) or one matched to a profile based on AA analysis of whole mice (BODYCOMPAA) (Kremen et al., 2013). Both are based on empirical data and are used to inform optimal dietary AA composition. We found no difference in initial growth rate of mice fed MOUSEAA versus those fed NRCAA (Figure 7D), while those assigned the diet based on body composition

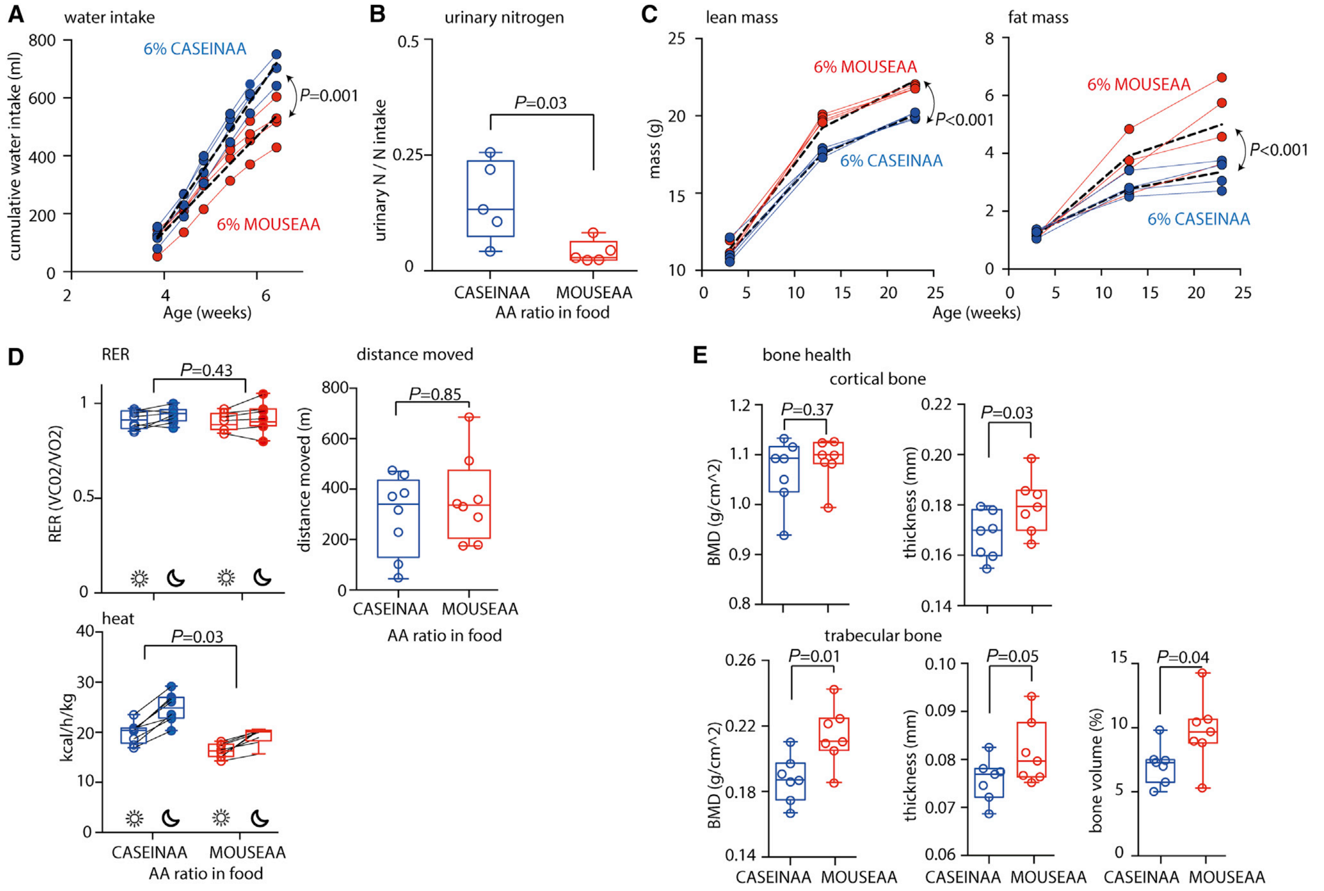

Figure 6. Exome Matching Broadly Alters Mouse Physiology

(A) Mice on CASEINAA voluntarily consumed 35% more water than those on MOUSEAA (p = 0.001). Cumulative water consumption per cage shown with heavy black dashed line showing statistical model fit. One of two independent trials is shown. Liner mixed effects model: AA ratio, time, and their interaction as main effects, and cage and its interaction with time were random effects.

(B) After 20 weeks of development on the media, MOUSEAA mice excreted a smaller proportion of their ingested nitrogen in urine than those fed CASEINAA (p = 0.03, Wilcoxon rank-sum test). Data show urinary nitrogen excretion from five individual mice. Collected in a single trial.

(C) Feeding on MOUSEAA caused a significant increase in the rate of accumulation of both fat (p < 0.001) and lean (p < 0.001) mass during 20 weeks of exposure. Measurements are from each of five mice in four cages (cage averages, colored lines) for both diets. Data gathered from a single trial. Linear model with mixed effects: AA ratio, ln(time), and their interaction were fixed effects, while the data from individual mice (nested within cages) and the slope of their mass accumulation were random effects.

(D) At night, RER of all mice was significantly increased (p = 0.01), but there was no effect of diet (p = 0.43, multivariate ANOVA). Total distance moved by mice was not different between diets (p = 0.85, t test). However, there was an increase in thermogenesis of mice on CASEINAA over those on MOUISEAA (p < 0.001), and the increase was more pronounced at night (p = 0.025, diet*time of day interaction, multivariate ANOVA). Data shown are normalized to lean mass; analysis using non-normalized data yielded the same outcomes qualitatively. Data are from eight individuals at 23–24 weeks of age from a single cohort.

(E) Mice that developed on MOUSEAA had significantly enhanced femur cortical thickness (p = 0.03), trabecular bone mineral density (BMD) (p = 0.01), and trabecular volume (p = 0.44) when compared with those reared on CASEINAA. t test. Data collected from one trial. Seven animals per condition.

See also Figure S6.

showed significantly slower growth (Figure 7E). Although not different in its effect during early linear growth, the NRCAA diet did, however, support an ~8% greater adult body mass (Figure S7C) that whole-body MRI revealed was due to the NRCAA-fed mice gaining fat, but not lean mass, at a faster rate than MOUSEAA-fed mice (Figure S7D). In further tests, we found no differences in movement, fecal energy content, RER, heat production, glucose tolerance, insulin tolerance, or any of the measured bone quality metrics at 23–24 weeks of age (data not shown). Thus, the enriched fat mass of the NRCAA mice may be due to differences in fat biosynthesis.

In summary, exome matching provides an easily implemented framework for establishing a high-quality nitrogen source for mouse growth. We note that its quantitative accuracy in mouse and fly growth was not as precise as for fly egg laying, perhaps due to nutritional buffering by resident microbiota similar to that observed in Schwarzer et al. (2016) and Wong et al. (2014). Nonetheless, our data provide evidence that our completely in silico method can be used for dietary AA design, and that it performs equally as well as, if not better than, natural diets or others that have been developed empirically over decades.

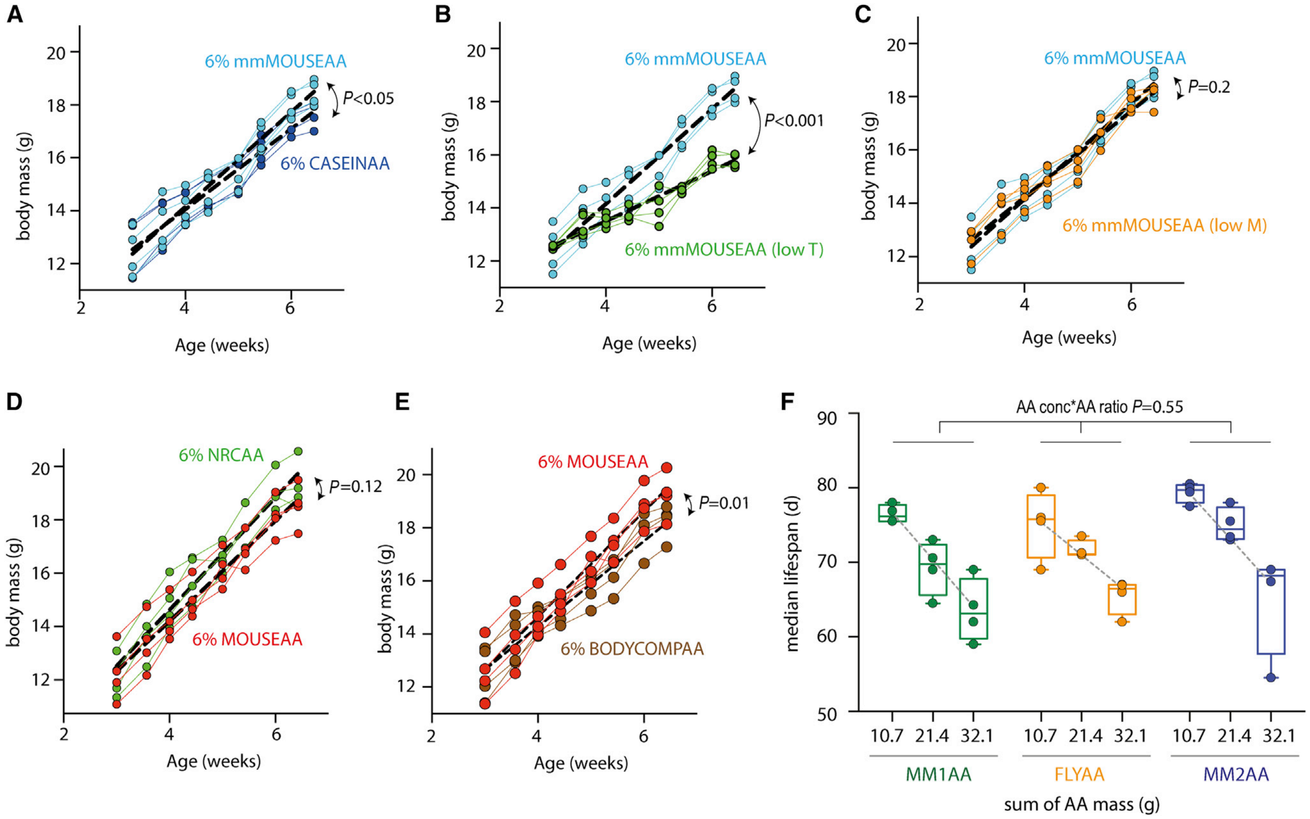

Figure 7. Exome Matching Alters Mouse Development, but Not Fly Lifespan

(A) CASEINAA and another MMAA profile (mmMOUSEAA) had similar, but significantly different ($p < 0.05$), growth rates.

(B and C) Reducing the AA (B) predicted to be limiting in mmMOUSEAA (T) reduced growth rate ($p < 0.001$), but reducing M (C), which was predicted by exome matching to be in excess, did not ($p = 0.22$).

(D) No growth rate difference was detectable for mice feeding on MOUSEAA versus those maintained on NRCAA ($p = 0.12$). See also Figure S7.

(E) Mice fed a diet with AA proportions according to whole-body AA analysis (BODYCOMPAA) had significantly slower growth rate than those on MOUSEAA ($p = 0.01$). Five mice in each of four cages per nutritional condition. Linear model with mixed effects: AA ratio, time, and their interaction were treated as fixed effects; individual mice nested within cages and the slope of their mass accumulation were random effects.

(F) For flies, the relative concentrations of dietary AAs altered median lifespan ($p < 0.001$), but with no effect of AA ratio, either alone ($p = 0.2$) or to modify the response to AA concentration ($p = 0.55$). Linear model with mixed effects: AA concentration and ratio as fixed effects and trial as a random effect. Medians from four trials. A total of 100 flies per condition were used for all trials, except one in which 200 were used.

Low Concentrations of Exome-Matched AAs Avoid the Lifespan/Reproduction Trade-Off

The relative concentration of protein in the diet has been shown to modulate both early life fitness and lifespan, and can account for the benefits of DR in flies and mice (Lee et al., 2008; Mair et al., 2005; Skorupa et al., 2008; Solon-Biet et al., 2014). We therefore assessed the response of *Drosophila* lifespan to varying concentrations of MM1AA, MM2AA, and FLYAA. Consistent with our previous observation (Piper et al., 2014), we found a marked increase in lifespan with decreasing relative concentration of total dietary AAs, an effect that was not modified by AA ratio (Figure 7F), unlike early life where the AA ratio strongly affected growth (Figure 5A) and egg laying (Figures 3E and 3F).

Interestingly, considering the egg laying and lifespan phenotypes together, their response differences caused flies on FLYAA to exhibit a single dietary optimum for growth, reproduction, and lifespan at a relatively low AA concentration (10.7 g/L). In contrast, flies on the MM diets displayed separate optima because egg laying and development required higher levels of AAs (at least 21.4 g/L) than the optimum for lifespan (10.7 g/L). Thus, as we have observed previously (Grandison et al., 2009), changing the dietary AA balance can establish a diet in which the apparent trade-off between reproduction and lifespan is avoided. Here we show that this balance can be established by matching the dietary AA ratio to the in silico-translated exome. In looking for correlated effects on nutrient signaling, we found that total 4e-BP levels, but not the proportion phosphorylated, were reduced in the ovaries of adult flies exposed to 10.7 g/L FLYAA when compared to those on the same concentration of AA in the MM diets (data not shown). In contrast, 4e-BP levels and phosphorylation were unaffected across the same treatments when measured in adult flies from which the ovaries had been removed. Thus, across all conditions we measured, we found that 4e-BP levels changed with growth and reproduction when measured in dividing tissue, and held steady with unchanging lifespan when measured in non-dividing tissue. These data are compatible with an explanation that tissues vary in their sensitivity to AA ratios for modulating IIS and

TOR, and that these inter-tissue differences may be key to separating the regulation of lifespan and reproduction.

Conclusion

Consuming a diet with a relatively low proportion of protein is critical for lifelong health (Le Couteur et al., 2016). However, the costs to early life vigor, reproduction, and low satiety value (Gosby et al., 2011; Solon-Biet et al., 2015; Sørensen et al., 2008) are major detractions. We show that an exome-matched AA composition can reduce voluntary food consumption, and that its enhanced value for growth and reproduction means it can be supplied at low enough levels so as to avoid any cost to lifespan. Given that these are all beneficial outcomes, it will be interesting to determine if there is a physiological cost that we have not yet measured.

It should be noted that the proportions of protein in our experimental diets are particularly low when viewed in the light of an average American diet containing ~15% protein (Simpson and Raubenheimer, 2005). While exome matching may be useful for screening supplements used to treat protein-energy malnutrition for AA imbalance, it is unlikely to enhance human growth or reproduction in developed countries where protein is generally non-limiting. However, because we have found that exome-matched diets represent high-quality protein that suppresses steady-state feeding at low concentrations, it is possible to envisage that diets to reduce total food intake to enhance long-term health, or even specifically to reduce the proportion of protein in the diet, e.g., for the management of kidney disease (Santesso et al., 2012), could be better achieved using exome matching.

Thus, the principle of exome matching provides a theoretical template for dietary AA design that can enhance the biological efficiency of food for the lifelong health of the consumer.

EXPERIMENTAL PROCEDURES

Additional details in the Supplemental Experimental Procedures.

General Fly Handling and Media

Except where indicated, all experiments were conducted with our female *Drosophila melanogaster* (Dahomey). Stocks are maintained outbred and experiments were conducted in controlled conditions: 25°C, 65% humidity, and 12 hr:12 hr light:dark. Except where indicated, flies were reared on 1× SY food at standard density (Bass et al., 2007). Holidic media were made according to Piper et al. (2014) with appropriate substitutions for each of the different AA ratios (Table S2; Data S1).

Mouse Strain, Housing, and Diets

For each mouse experiment, 20 C3B6F1/J females per treatment were used, housed in four groups of five under specific-pathogen-free (SPF) conditions. Our parental mice were two inbred strains: C57BL/6J and C3H from the Jackson Laboratory.

Except for ad libitum-fed animals, mice were pair fed. Water intake was measured for each cage separately twice per week. Diets were manufactured by Ssniff.

Mouse experiments were performed in accordance with the recommendations and guidelines of the Federation of the European Laboratory Animal Science Association (FELASA), with all protocols approved by the Landesamt für Natur, Umwelt und Verbraucherschutz Nordrhein-Westfalen, Germany.

Exome Matching

A total of 21,070 proteins were retrieved from FlyBase (FB2008_05, released May 30, 2008). From this, 821 proteins with length less than 100 AAs and 513 with length greater than 2,000 AAs were removed to generate a set of 19,736 non-extreme proteins. This trimming procedure was followed for all animal genomes used. For mouse, Ensemble v 54 (May 2009, downloaded July 2, 2009) was used and for rat, Ensemble v 56 (September 2009, downloaded January 19, 2009). The sum of each AA used in each protein was used to generate its proportional AA usage, and this was combined for all proteins to find the average AA usage for the predicted proteome.

To predict the most limiting EAA in a diet, the proportion of AAs in the food was divided by the proportional representation of AAs in the translated exome of the consumer. The EAA with the lowest value after this transformation was considered limiting. If the requirement for either of the conditionally EAAs Y or C exceeded the available supply, their requirement was met by subtracting a mole of F or M, respectively, for each mole of AA required.

To design MM2AA, we determined the Euclidian distance from MM1AA to FLYAA in 20-dimensional space (1 dimension per AA). We then found another point, MM2AA, that was equidistant from FLYAA as MM1AA, but as far away as possible from MM1AA. This procedure was also performed to generate mmMOUSEAA, using CASEINAA and MOUSEAA as starting points.

Fly Diet Preference and Feeding Assays

For each trial of each feeding assay, a fresh generation of flies was reared to avoid the possible confounding effects of memory-based decision making.

Holidic Diet Choice Assay

A four-arm choice apparatus modified from Cooper (1960) was used. Mated females were AA deprived for 72 hr and acclimatized in the chamber, and their subsequent location was counted hourly for ~8 hr. The food preference index (FPI) was calculated as (n flies on surface of food A − n flies on surface of food B)/(n flies on surface of food A + n flies on surface of food B). Total AA mass in food was fixed at 21.4 g/L.

SY Choice Assay

Groups of 3- to 5-day-old flies (15 females and 5 males) were maintained on yeast-based food or holidic medium. After 72 hr, the flies were tested for nutrient choice as described in Ribeiro and Dickson (2010).

flyPAD Monitoring of Feeding Behavior

w Dahomey flies were reared in the same medium as for the SY choice assay. Mated adult flies were then maintained on either holidic or yeast-based medium for the pretreatment period (3 days) and assayed using flyPAD, as described in Itskov et al. (2014).

Measuring Fly Development

First instar larvae were picked onto test media and each developmental stage scored at 25, 138, 258, and 330 hr after egg laying. Adult eclosion was scored daily at 24 hr intervals. Body mass was measured for pairs of newly emerged flies. Wings were measured from the edge of the distal tip to the edge of the alula.

Fly Uric Acid and TAG Measurements

After 16 hr on holidic medium, flies and medium were removed from the vial, which was washed with 2 mL of 0.1 M sodium glycinate buffer (pH 9.2). Uric acid was quantified spectrophotometrically using the Amplex Red Uric Acid kit (Life Technologies). For TAG determinations, we used the Triglyceride Infinity reagent (Thermo Scientific) and normalized levels to total protein.

Fly Westerns

The following antibodies were used: 4EBP1 (CST #4923), phospho-4EBP1 (CST #4923), S6K (custom-made, courtesy IHA, UCL), and phospho-S6K (CST #9209).

Fly Lifespans

Lifespan assays were performed as described in Piper and Partridge (2016). Replicates 1–3 were performed preparing the holidic medium as described in Piper et al. (2014), but for the fourth replicate, the three AAs (I, L, and Y) were added after autoclaving.

Mouse Physiological Measurements

Body fat content was determined by in vivo magnetic resonance tomography imaging (time domain [TD] NMR).

Indirect calorimetry and movement were monitored over 48 hr for singly housed mice in purpose-built cages (Phenomaster, TSE systems) maintained at 22°C –23°C.

Urea in urine from a 24 hr period was measured using the Urea Assay kit (Sigma) from mice housed in metabolic cages (Tecniplast).

Portal Vein Plasma Metabolite Analysis

Portal vein blood samples were harvested from pair-fed female C3B6F1/J mice at 23 weeks of age following a 1 g meal and 2 hr food deprivation. Mice were euthanized and dissected, and portal vein blood was collected in EDTA tubes. Analysis was performed by the Finnish Institute for Molecular Medicine (FIMM).

Mouse Tissue and Bone Measurements

At 23 weeks of age, mice were euthanized using CO_2 and organs were immediately harvested and weighed. For bone density measurements, right femur bones from 23-week-old mice were collected and scanned with a high-resolution μCT scanner (SkyScan 1176, Bruker). Trabecular and cortical bone regions of distal femurs were selected with reference to the growth plate. Bone mineral density was determined based on calibration with two phantoms of known density (Bruker), which were scanned under the same conditions as the bone samples.

Statistical Analyses

R (v3.2.0) and JMP (V11) were used for all statistical analyses. Generally, error bars represent SEM.

SUPPLEMENTAL INFORMATION

Supplemental Information includes Supplemental Experimental Procedures, seven figures, two tables, and one data file and can be found with this article online at http://dx.doi.org/10.1016/j.cmet.2017.02.005.

AUTHOR CONTRIBUTIONS

Conceptualization, M.D.W.P. and L.P.; Methodology, M.D.W.P., G.A.S., E.B., I.A., S.J.S., C.R., and L.P.; Formal Analysis, M.D.W.P., G.A.S., E.B., S.L.H., I.A., C.R., and L.P.; Investigation, M.D.W.P., G.A.S., A.M., S.L.H., P.J., X.H., H.S., and M.Y.; Writing – Original Draft, M.D.W.P. and L.P.; Writing – Review & Editing, M.D.W.P., G.A.S., S.J.S., C.R., and L.P.; Funding Acquisition, M.D.W.P., C.R., and L.P.

ACKNOWLEDGMENTS

Funding was provided by the Australian Research Council (FT150100237), the Royal Society (UF100158 and RG110303), and the Biotechnology and Biological Sciences Research Council, UK (BB/I011544/1) to M.D.W.P.; National Natural Science Foundation of China (31471998) to M.Y.; the Champalimaud Foundation, the BIAL Foundation (project #283/14), and the Human Frontier Science Program (project grant RGP0022/2012) to C.R.; Portuguese Foundation for Science and Technology (FCT) PhD Fellowship SRFH/BD/76066/2011 to S.L.H.; the Australian National Health and Medical Research Council (project grant 1084267) to S.J.S.; and the Wellcome Trust UK (098565/Z/12/Z), Max Planck Society and the European Research Council under the European Union's Seventh Framework Programme (FP7/2007-2013), and European Research Council grant agreement 268739 to L.P. We also thank Christoph Dieterich (Bioinformatics Core Facility, Max Planck Institute for Biology of Ageing) for discussions and coordinating the fly SILAC data with Sury et al.; Wilfried Haider (Max Planck Institute for Metabolic Research) for constructing the four-arm food choice chambers for *Drosophila*; and Sahar Emran, Xueping Mao, André Pahl, Martin Purrio, and Nina Grisard for assistance with some experiments. We thank the Comparative Biology and Phenotyping Core Facility at the Max Planck Institute for Biology of Ageing for help with mouse husbandry and phenotyping. Fecal energy analysis was carried out at the Mouse Metabolic Facility (MEF), University of Lausanne.

Received: July 26, 2016
Revised: December 22, 2016
Accepted: February 9, 2017
Published: March 7, 2017

REFERENCES

Bass, T.M., Grandison, R.C., Wong, R., Martinez, P., Partridge, L., and Piper, M.D. (2007). Optimization of dietary restriction protocols in Drosophila. J. Gerontol. A Biol. Sci. Med. Sci. *62*, 1071–1081.

Breslin, T., Edén, P., and Krogh, M. (2004). Comparing functional annotation analyses with Catmap. BMC Bioinformatics *5*, 193.

Bülow, M.H., Aebersold, R., Pankratz, M.J., and Jünger, M.A. (2010). The Drosophila FoxA ortholog Fork head regulates growth and gene expression downstream of Target of rapamycin. PLoS ONE *5*, e15171.

Chintapalli, V.R., Wang, J., and Dow, J.A. (2007). Using FlyAtlas to identify better Drosophila melanogaster models of human disease. Nat. Genet. *39*, 715–720.

Cooper, D.M. (1960). Food preferences of larval and adult Drosophila. Evolution *14*, 41–55.

Corrales-Carvajal, V.M.M., Faisal, A.A., and Ribeiro, C. (2016). Internal states drive nutrient homeostasis by modulating exploration-exploitation trade-off. eLife *5*, http://dx.doi.org/10.7554/eLife.19920.

Dick, K.B., Ross, C.R., and Yampolsky, L.Y. (2011). Genetic variation of dietary restriction and the effects of nutrient-free water and amino acid supplements on lifespan and fecundity of Drosophila. Genet. Res. *93*, 265–273.

Gosby, A.K., Conigrave, A.D., Lau, N.S., Iglesias, M.A., Hall, R.M., Jebb, S.A., Brand-Miller, J., Caterson, I.D., Raubenheimer, D., and Simpson, S.J. (2011). Testing protein leverage in lean humans: a randomised controlled experimental study. PLoS ONE *6*, e25929.

Grandison, R.C., Piper, M.D., and Partridge, L. (2009). Amino-acid imbalance explains extension of lifespan by dietary restriction in Drosophila. Nature *462*, 1061–1064.

Hao, S., Sharp, J.W., Ross-Inta, C.M., McDaniel, B.J., Anthony, T.G., Wek, R.C., Cavener, D.R., McGrath, B.C., Rudell, J.B., Koehnle, T.J., and Gietzen, D.W. (2005). Uncharged tRNA and sensing of amino acid deficiency in mammalian piriform cortex. Science *307*, 1776–1778.

Hinton, T. (1956). The effects of arginine, ornithine and citrulline on the growth of Drosophila. Arch. Biochem. Biophys. *62*, 78–85.

Hunt, V. (1970). A qualitatively minimal amino acid diet for D. melanogaster. Drosoph. Inf. Serv. *45*, 179.

Itskov, P.M., Moreira, J.-M.M., Vinnik, E., Lopes, G., Safarik, S., Dickinson, M.H., and Ribeiro, C. (2014). Automated monitoring and quantitative analysis of feeding behaviour in Drosophila. Nat. Commun. *5*, 4560.

Jünger, M.A., Rintelen, F., Stocker, H., Wasserman, J.D., Végh, M., Radimerski, T., Greenberg, M.E., and Hafen, E. (2003). The Drosophila forkhead transcription factor FOXO mediates the reduction in cell number associated with reduced insulin signaling. J. Biol. *2*, 20.

Kirkwood, T.B. (1977). Evolution of ageing. Nature *270*, 301–304.

Koehnle, T.J., Russell, M.C., and Gietzen, D.W. (2003). Rats rapidly reject diets deficient in essential amino acids. J. Nutr. *133*, 2331–2335.

Kremen, N.A., Calvert, C.C., Larsen, J.A., Baldwin, R.A., Hahn, T.P., and Fascetti, A.J. (2013). Body composition and amino acid concentrations of select birds and mammals consumed by cats in northern and central California. J. Anim. Sci. *91*, 1270–1276.

Layalle, S., Arquier, N., and Léopold, P. (2008). The TOR pathway couples nutrition and developmental timing in Drosophila. Dev. Cell *15*, 568–577.

Le Couteur, D.G., Solon-Biet, S., Cogger, V.C., Mitchell, S.J., Senior, A., de Cabo, R., Raubenheimer, D., and Simpson, S.J. (2016). The impact of low-protein high-carbohydrate diets on aging and lifespan. Cell. Mol. Life Sci. *73*, 1237–1252.

Lee, K.P., Simpson, S.J., Clissold, F.J., Brooks, R., Ballard, J.W., Taylor, P.W., Soran, N., and Raubenheimer, D. (2008). Lifespan and reproduction in Drosophila: New insights from nutritional geometry. Proc. Natl. Acad. Sci. USA *105*, 2498–2503.

Levine, M.E., Suarez, J.A., Brandhorst, S., Balasubramanian, P., Cheng, C.-W., Madia, F., Fontana, L., Mirisola, M.G., Guevara-Aguirre, J., Wan, J., et al. (2014). Low protein intake is associated with a major reduction in

IGF-1, cancer, and overall mortality in the 65 and younger but not older population. Cell Metab. *19*, 407–417.

Mair, W., Piper, M.D., and Partridge, L. (2005). Calories do not explain extension of life span by dietary restriction in Drosophila. PLoS Biol. *3*, e223.

Miller, R.A., Buehner, G., Chang, Y., Harper, J.M., Sigler, R., and Smith-Wheelock, M. (2005). Methionine-deficient diet extends mouse lifespan, slows immune and lens aging, alters glucose, T4, IGF-I and insulin levels, and increases hepatocyte MIF levels and stress resistance. Aging Cell *4*, 119–125.

Millward, D.J., Layman, D.K., Tomé, D., and Schaafsma, G. (2008). Protein quality assessment: impact of expanding understanding of protein and amino acid needs for optimal health. Am. J. Clin. Nutr. *87*, 1576S–1581S.

National Research Council (1995). Nutrient Requirements of Laboratory Animals, Fourth, Revised Edition (National Academies Press).

Markow, T.A. (2015). The secret lives of Drosophila flies. eLife *4*, e06793.

Piper, M.D., and Bartke, A. (2008). Diet and aging. Cell Metab. *8*, 99–104.

Piper, M.D., and Partridge, L. (2016). Protocols to study aging in Drosophila. Methods Mol. Biol. *1478*, 291–302.

Piper, M.D., Blanc, E., Leitão-Gonçalves, R., Yang, M., He, X., Linford, N.J., Hoddinott, M.P., Hopfen, C., Soultoukis, G.A., Niemeyer, C., et al. (2014). A holidic medium for Drosophila melanogaster. Nat. Methods *11*, 100–105.

Ribeiro, C., and Dickson, B.J. (2010). Sex peptide receptor and neuronal TOR/S6K signaling modulate nutrient balancing in Drosophila. Curr. Biol. *20*, 1000–1005.

Rogers, Q.R., and Harper, A.E. (1965). Amino acid diets and maximal growth in the rat. J. Nutr. *87*, 267–273.

Rozin, P. (1967). Specific aversions as a component of specific hungers. J. Comp. Physiol. Psychol. *64*, 237–242.

Santesso, N., Akl, E.A., Bianchi, M., Mente, A., Mustafa, R., Heels-Ansdell, D., and Schünemann, H.J. (2012). Effects of higher- versus lower-protein diets on health outcomes: a systematic review and meta-analysis. Eur. J. Clin. Nutr. *66*, 780–788.

Schwarzer, M., Makki, K., Storelli, G., Machuca-Gayet, I., Srutkova, D., Hermanova, P., Martino, M.E., Balmand, S., Hudcovic, T., Heddi, A., et al. (2016). Lactobacillus plantarum strain maintains growth of infant mice during chronic undernutrition. Science *351*, 854–857.

Shingleton, A.W. (2010). The regulation of organ size in Drosophila: physiology, plasticity, patterning and physical force. Organogenesis *6*, 76–87.

Simpson, S.J., and Raubenheimer, D. (2005). Obesity: the protein leverage hypothesis. Obes. Rev. *6*, 133–142.

Simpson, S.J., and Raubenheimer, D. (2012). The Nature of Nutrition: A Unifying Framework from Animal Adaptation to Human Obesity (Princeton University Press).

Skorupa, D.A., Dervisefendic, A., Zwiener, J., and Pletcher, S.D. (2008). Dietary composition specifies consumption, obesity, and lifespan in Drosophila melanogaster. Aging Cell *7*, 478–490.

Solon-Biet, S.M., McMahon, A.C., Ballard, J.W., Ruohonen, K., Wu, L.E., Cogger, V.C., Warren, A., Huang, X., Pichaud, N., Melvin, R.G., et al. (2014). The ratio of macronutrients, not caloric intake, dictates cardiometabolic health, aging, and longevity in ad libitum-fed mice. Cell Metab. *19*, 418–430.

Solon-Biet, S.M., Mitchell, S.J., Coogan, S.C., Cogger, V.C., Gokarn, R., McMahon, A.C., Raubenheimer, D., de Cabo, R., Simpson, S.J., and Le Couteur, D.G. (2015). Dietary protein to carbohydrate ratio and caloric restriction: comparing metabolic outcomes in mice. Cell Rep. *11*, 1529–1534.

Sørensen, A., Mayntz, D., Raubenheimer, D., and Simpson, S.J. (2008). Protein-leverage in mice: the geometry of macronutrient balancing and consequences for fat deposition. Obesity (Silver Spring) *16*, 566–571.

Soultoukis, G.A., and Partridge, L. (2016). Dietary protein, metabolism, and aging. Annu. Rev. Biochem. *85*, 5–34.

Sury, M.D., Chen, J.-X., and Selbach, M. (2010). The SILAC fly allows for accurate protein quantification in vivo. Mol. Cell. Proteomics *9*, 2173–2183.

Walker, S.J., Corrales-Carvajal, V.M.M., and Ribeiro, C. (2015). Postmating circuitry modulates salt taste processing to increase reproductive output in Drosophila. Curr. Biol. *25*, 2621–2630.

Williams, G.C. (1966). Natural selection, the costs of reproduction, and a refinement of Lack's principle. Am. Nat. *100*, 687–690.

Wong, A.C., Dobson, A.J., and Douglas, A.E. (2014). Gut microbiota dictates the metabolic response of Drosophila to diet. J. Exp. Biol. *217*, 1894–1901.

Cell Metabolism, Volume *25*

Supplemental Information

Matching Dietary Amino Acid Balance to the In Silico-Translated Exome Optimizes Growth and Reproduction without Cost to Lifespan

Matthew D.W. Piper, George A. Soultoukis, Eric Blanc, Andrea Mesaros, Samantha L. Herbert, Paula Juricic, Xiaoli He, Ilian Atanassov, Hanna Salmonowicz, Mingyao Yang, Stephen J. Simpson, Carlos Ribeiro, and Linda Partridge

Supplemental Figures and Legends

Figure S1

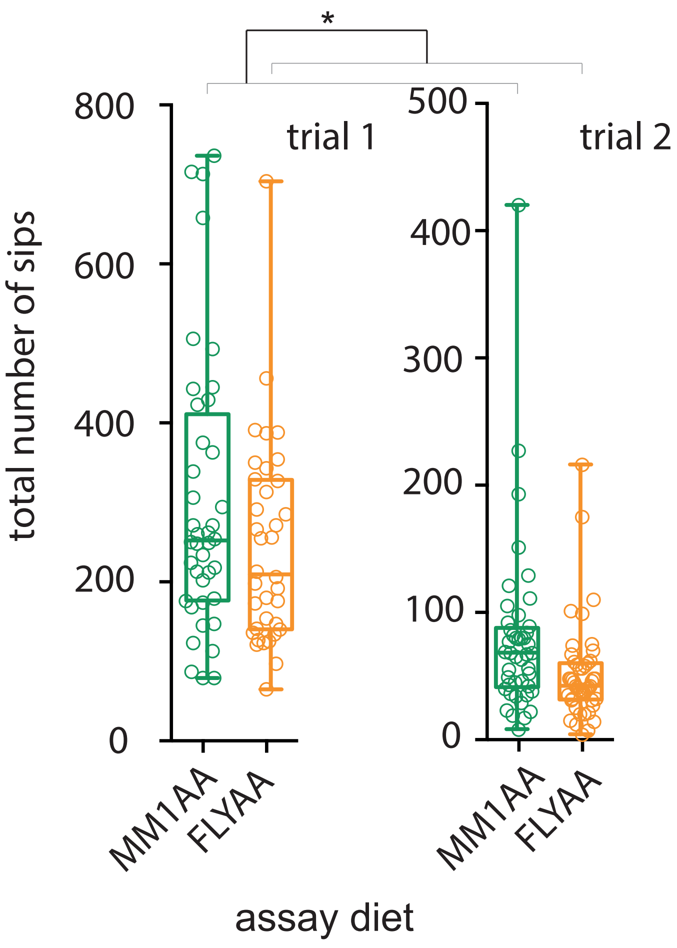

Figure S1. Flies feed slightly less on FLYAA than MM1AA. (See also Figure 2)
Female flies maintained on the indicated food type for 3 days were measured for feeding behaviour using flyPAD (Itskov et al., 2014). Across two experimental blocks, flies were found to take fewer sips when maintained on FLYAA than those maintained on MM1AA (*P*=0.002). (Linear mixed effects model, AA ratio as fixed effect and experimental block as random effect. In block one, 40 biological replicates for FLYAA, 36 for MM1AA and block 2 there were 45 biological replicates and 48 biological replicates per treatment respectively).

Figure S2

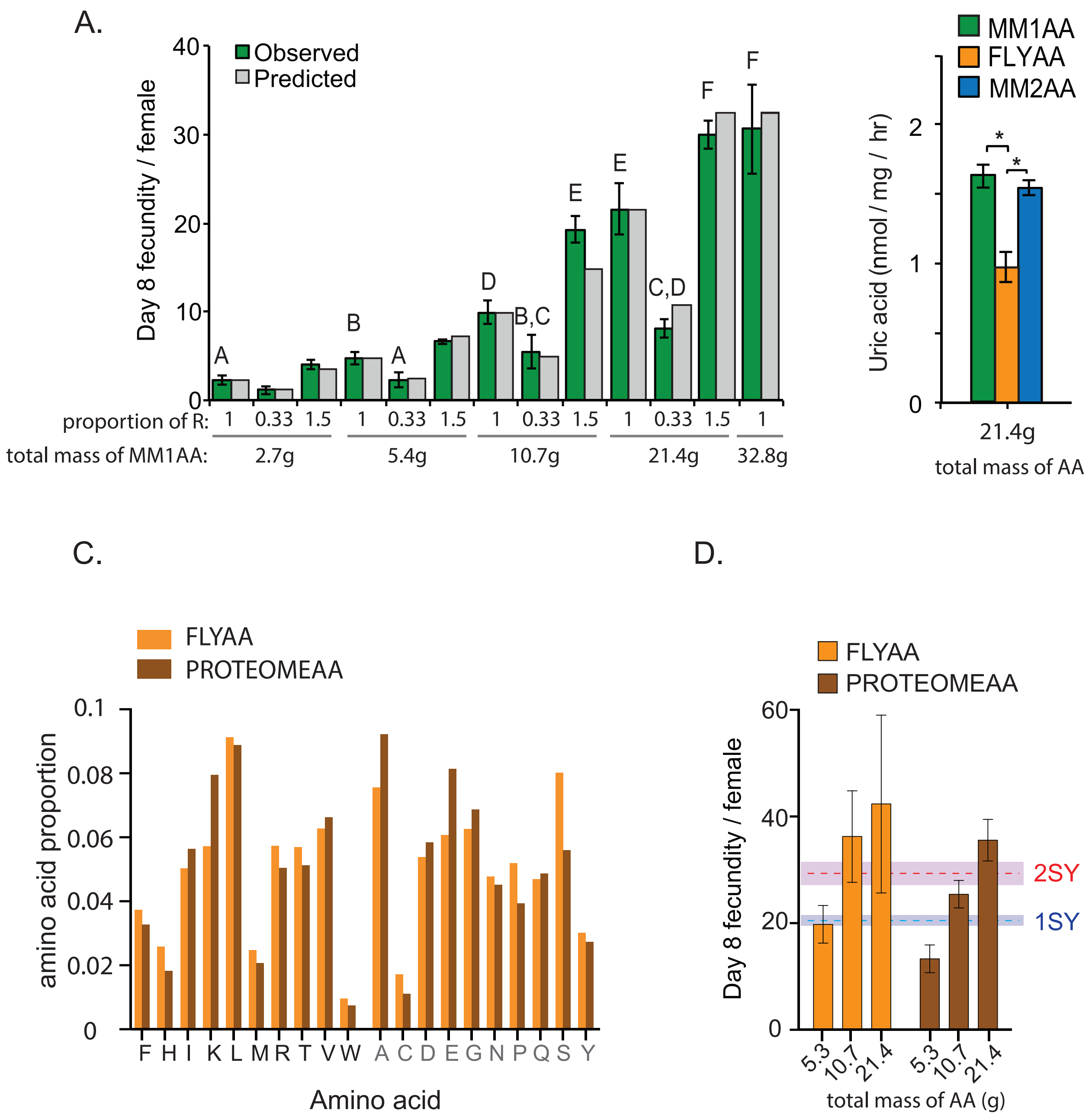

Figure S2. Exome matching predicts both the identity and the degree to which essential amino acids in a complex mixture limit egg laying. (See also Figure 3).

(a) Over a wide range of total amino acid mass additions, altering the concentration of arginine (R) in MM1AA produced a proportionally matched change in egg laying.

(b) Uric acid excretion was measured for flies feeding on media containing each of the three amino acid ratios. During 16 hours, flies on FLYAA produced ~60% of the amount of uric acid as flies feeding on either MM1AA or MM2AA ($P<0.05$, T-test). Data from 4 biological repeats with 10 flies per treatment in each trial.

(d) Egg laying for females maintained on varying concentrations of FLYAA or an amino acid ratio derived from published measured body composition data using whole fly proteomics data (Sury et al., 2010). Comparing egg laying on FLYAA v PROTEOMEAA revealed that only the effect of amino acid mass ($P<0.001$), and not the ratio, or the interaction between the two ($P>0.58$ for both comparisons), had a significant effect on egg laying. (Linear model with mixed effects: Ln (aa mass) and amino acid ratio as fixed effects, modelling the response of egg laying (sqrt transformed). Trial date was set as random effect). N=3. Each replicate employed 6 vials containing 10 flies each. In panels a, b & d, data are presented as mean ± s.e.

Figure S3

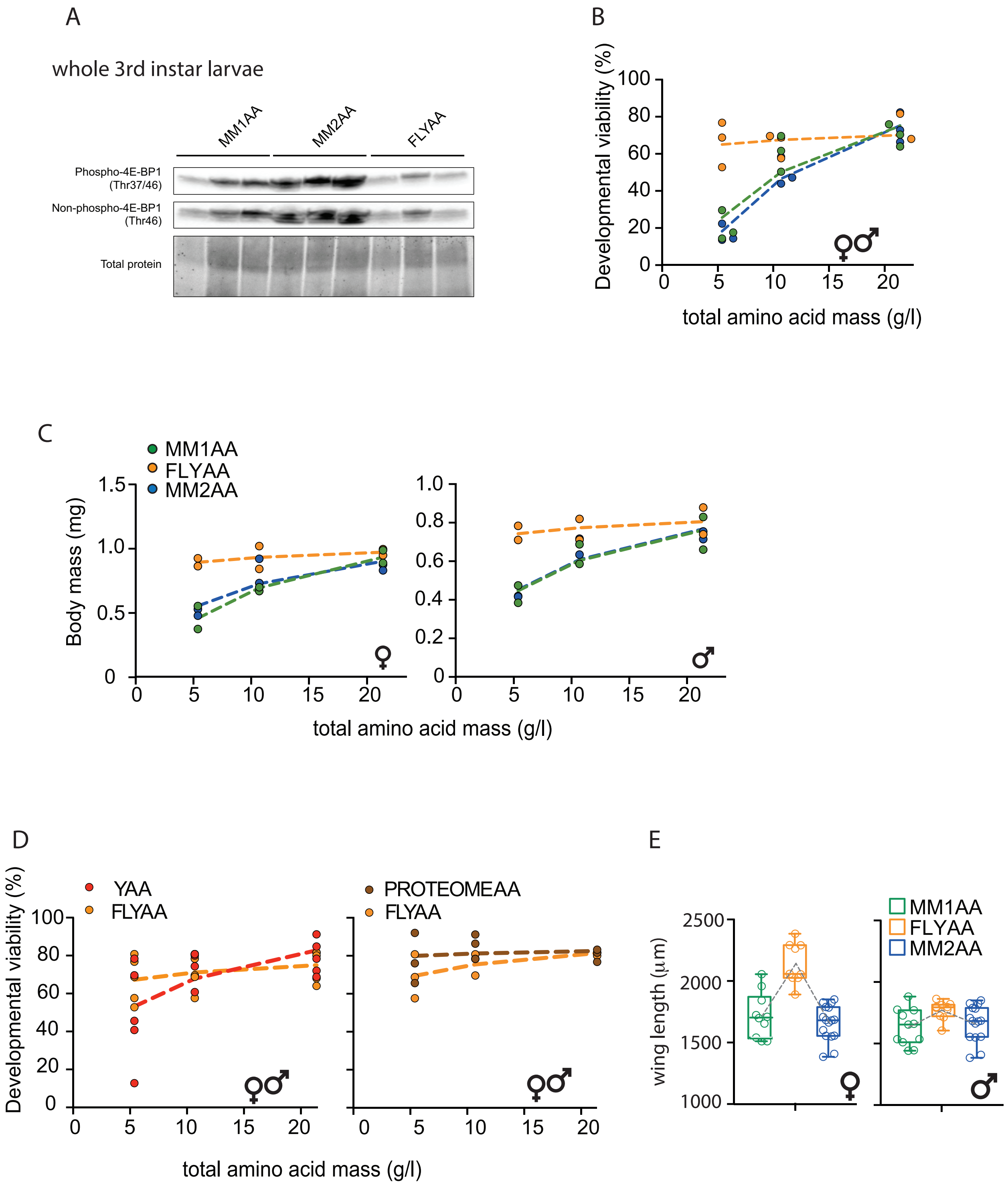

Figure S3. Effect of altering amino acid ratio and mass on developmental timing, viability and adult body mass. (See also Figure 5).
(a) image of Western blot corresponding to quantification in Figure 5c.
(b) Amino acid mass, amino acid ratio and their interaction showed significant effects on viability due to the enhanced viability at the lower concentrations of FLYAA v MM1AA, MM2AA. (*P*<0.0001 for fixed effect; linear

model using log transformed amino acid masses; dashed lines show model estimates). 3 trials were run with 5 vials per food type and 25 eggs per vial.
(c) Body masses of pairs of newly emerged flies were determined for duplicate trials, containing between 2-44 measurements per food, depending on availability of emerged flies. There was a significant effect of trial (P=0.019 for females, P=0.003 for males), amino acid ratio (P<0.001 for females and males) and amino acid mass (P<0.0001 for females and males), however trial date did not modify the other main effects. Amino acid ratio altered the manner in which amino acid mass altered body mass (P=0.006 for females, P=0.005 for males) in such a way that lower amounts of FLYAA supported higher body masses of females and males when compared with the two mismatched ratios. (Linear model using log transformed amino acid masses; dashed lines show model estimates).
(d) There were significant effects of amino acid ratio (P=0.04), amount (P=0.002) and their interaction (P=0.048) for larval viability on YAA v FLYAA. N=5. For PROTEOMEAA v FLYAA, no significant effects were observed. N = 3. In both panels, amino acid ratio and amount (log transformed) and their interaction were fixed effects with trial date assigned as a random effect (Linear mixed effects model).
(e) Wings from 10 day old females and males that had developed on each of the indicated AA ratios (total mass 10.7g/l) were removed and mounted on microscope slides. Length measurements were taken from the distal tip of the wing to the edge of the alula (closest point to the fly).

Figure S4

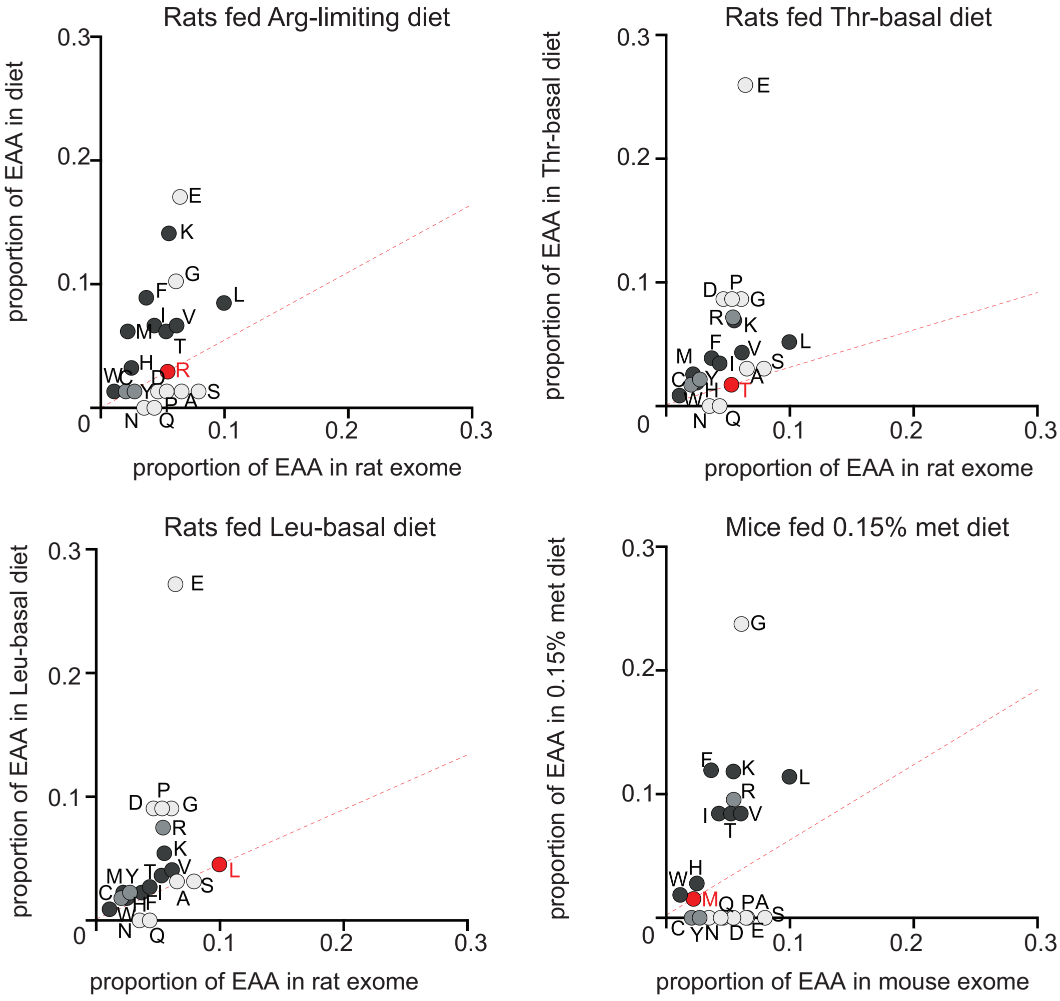

Figure S4. Exome matching correctly predicts the identity of the limiting amino acid (see also Figure 5). In four different studies in which the diet of rodents were fed amino acid imbalanced diets and the most limiting AA confirmed experimentally, exome matching identified the most limiting amino acid (red point). EAAs in dark grey, conditionally essential AAs in mid grey and non-essentials in light grey. Note that for each study, some non-essential AAs were not supplied at all in the diet (points that fall along the x-axis).

Figure S5

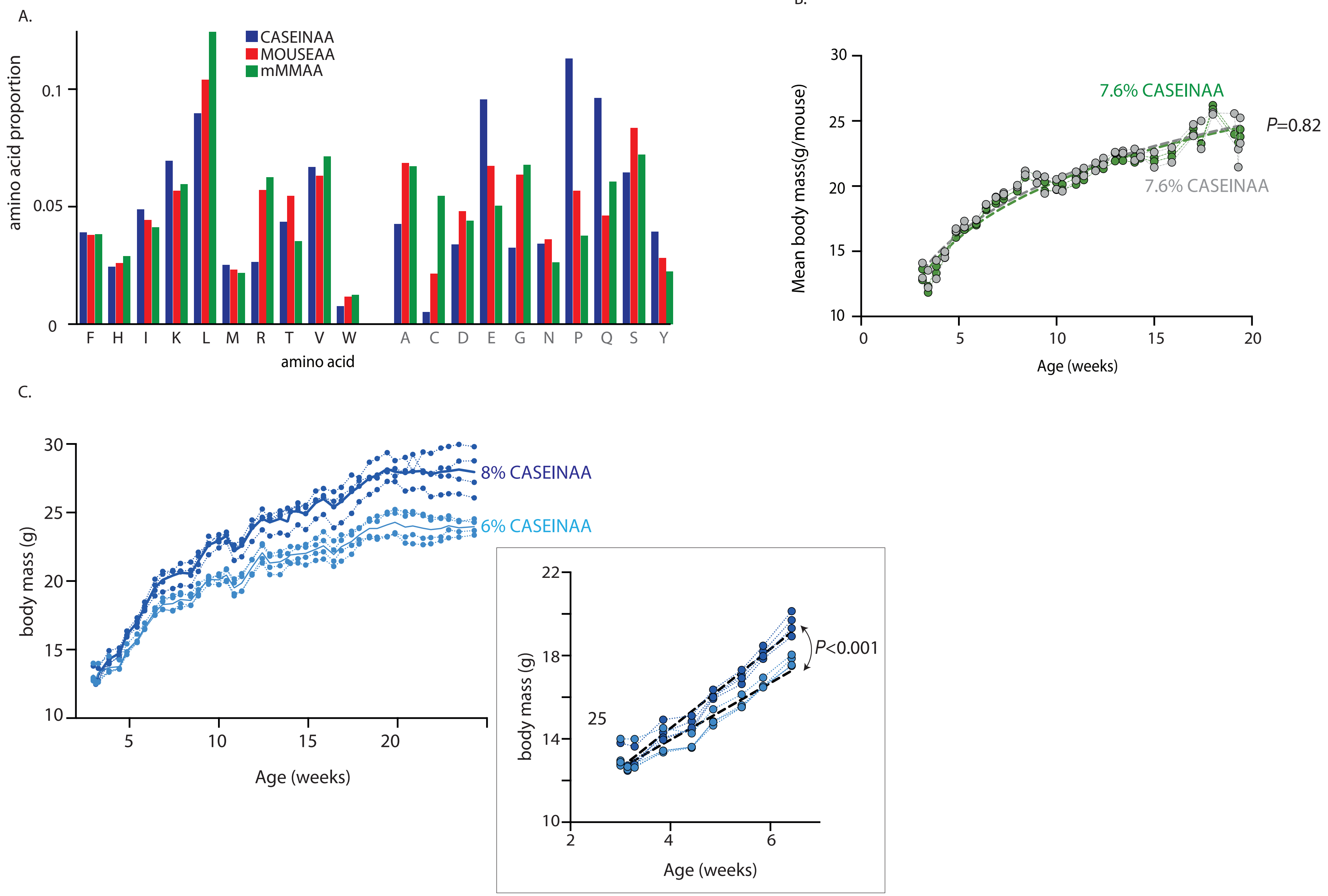
A.
CASEINAA
MOUSEAA
mMMAA
amino acid proportion
0.1
0.05
0
F H I K L M R T V W A C D E G N P Q S Y
amino acid
B.
Mean body mass(g/mouse)
30
25
20
15
10
0 5 10 15 20
Age (weeks)
7.6% CASEINAA
7.6% CASEINAA
P=0.82
C.
body mass (g)
30
25
20
15
10
5 10 15 20
Age (weeks)
8% CASEINAA
6% CASEINAA
body mass (g)
22
18
14
10
2 4 6
Age (weeks)
25
P<0.001

Figure S5. Exome matching and protein restricted mouse growth (see also Figure 5, 6 & 7)
(a) Proportion of amino acids in CASEINAA, MOUSEAA and mouseMMAA
(b) Under protein-limiting conditions (7.5% CP equivalents) diets consisting of purified amino acids or whole protein, as casein, supported growth equally well (P=0.82 for comparing the effect of foods over time; linear model with mixed effects, specifying log time and food type as fixed effects and mice, nested within cages, and their change over time, as random effects). Each of 2 cages (connected circles) per treatment contained 5 mice. Estimates from statistical model plotted as heavy dashed line. Data collected from a single trial.
(c) Casein is the standard protein source for mice in chow. We provided amino acids in growth limiting amounts, such that a 33% increase in total mass (from 6% to 8%) was reflected by a similar, but not proportionally matched (~40%), increase in growth rate. Average mass of mice in each cage plotted as individual points, line average for all mice in each treatment. Inset plot shows only initial linear growth phase (week 3- week 6.5). Individual points connected by dotted lines show cage averages, heavy dashed lines show line fit using parameters from statistical model. (P<0.0001 for slope change, Linear model mixed effects). 5 mice were housed in each of 4 cages per treatment. Data collected from a single trial.

Figure S6

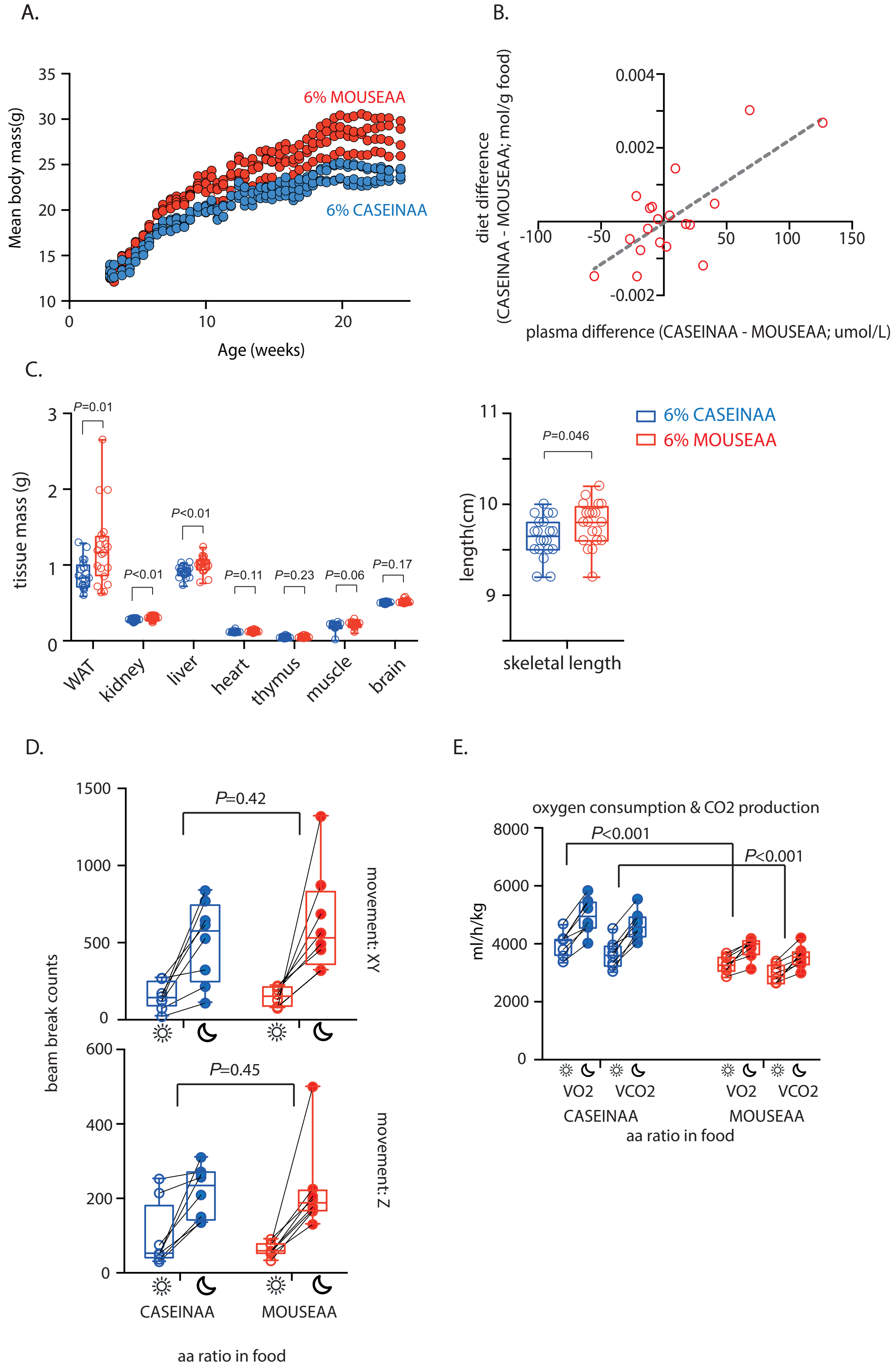
A.
B.
35
30
25
20
15
10
0
10
20
Mean body mass(g)
Age (weeks)
6% MOUSEAA
6% CASEINAA
0.004
0.002
-0.002
-100
-50
50
100
150
diet difference
(CASEINAA - MOUSEAA; mol/g food)
plasma difference (CASEINAA - MOUSEAA; umol/L)
C.
tissue mass (g)
P=0.01
P<0.01
P<0.01
P=0.11
P=0.23
P=0.06
P=0.17
WAT
kidney
liver
heart
thymus
muscle
brain
11
10
9
length(cm)
P=0.046
skeletal length
6% CASEINAA
6% MOUSEAA
D.
1500
1000
500
0
P=0.42
movement: XY
beam break counts
600
400
200
P=0.45
movement: Z
CASEINAA
MOUSEAA
aa ratio in food
E.
oxygen consumption & CO2 production
8000
6000
4000
2000
ml/h/kg
P<0.001
P<0.001
VO2
VCO2
VO2
VCO2
CASEINAA
MOUSEAA
aa ratio in food

Figure S6. Growth and metabolic characterisation of mice developing on CASEINAA versus MOUSEAA (see also Figure 6).

(a) Growth data for mice kept on MOUSEAA and CASEINAA from week 3-24 (continuation of Figure 5d).
(b) Measured differences in the concentration of free amino acids in blood from the hepatic portal vein positively correlated with differences in food (R^2=0.54; *P*<0.001 non-zero slope). Data collected from 23w old mice that had been maintained on CASEINAA (4 mice) or MOUSEAA (6 mice). Each point in the correlation represents a different amino acid.
(c) Mice were euthanized at 24 weeks and organs removed for weighing. White adipose tissue (WAT), kidney and liver were all significantly heavier in mice from MOUSEAA diet, while no change was detected for the heart, thymus, muscle (right quadriceps) and brain. (Tissues from 20 different individuals for each diet were removed and weighed. T-tests were used to compare tissues between diets where the data was normally distributed, while Wilcoxon rank sum test was used for the remainder).
(d) Movement, measured by counts of light beam breaks in the X, Y and Z planes, revealed no differences in patterns of activity at day or night between CASEINAA and MOUSEAA-fed mice (*P*>0.42 for effect of food type on activity or food type*time of day interaction, MANOVA).
(e) Rates of O_2 consumption and CO_2 production were significantly higher in CASEINAA mice than MOUSEAA mice (*P*<0.00). What's more, CASEINAA mice had increased gas exchange elevation at night compared with MOUSEAA mice (*P*<0.03 when comparing either VO2 or VCO2 diet*time of day interaction, MANOVA). Data in (d & e) are from 8 individuals at 23-24 weeks of age (20-21 weeks of treatment) from a single cohort of mice, each maintained in the Phenomaster for 48h.

Figure S7

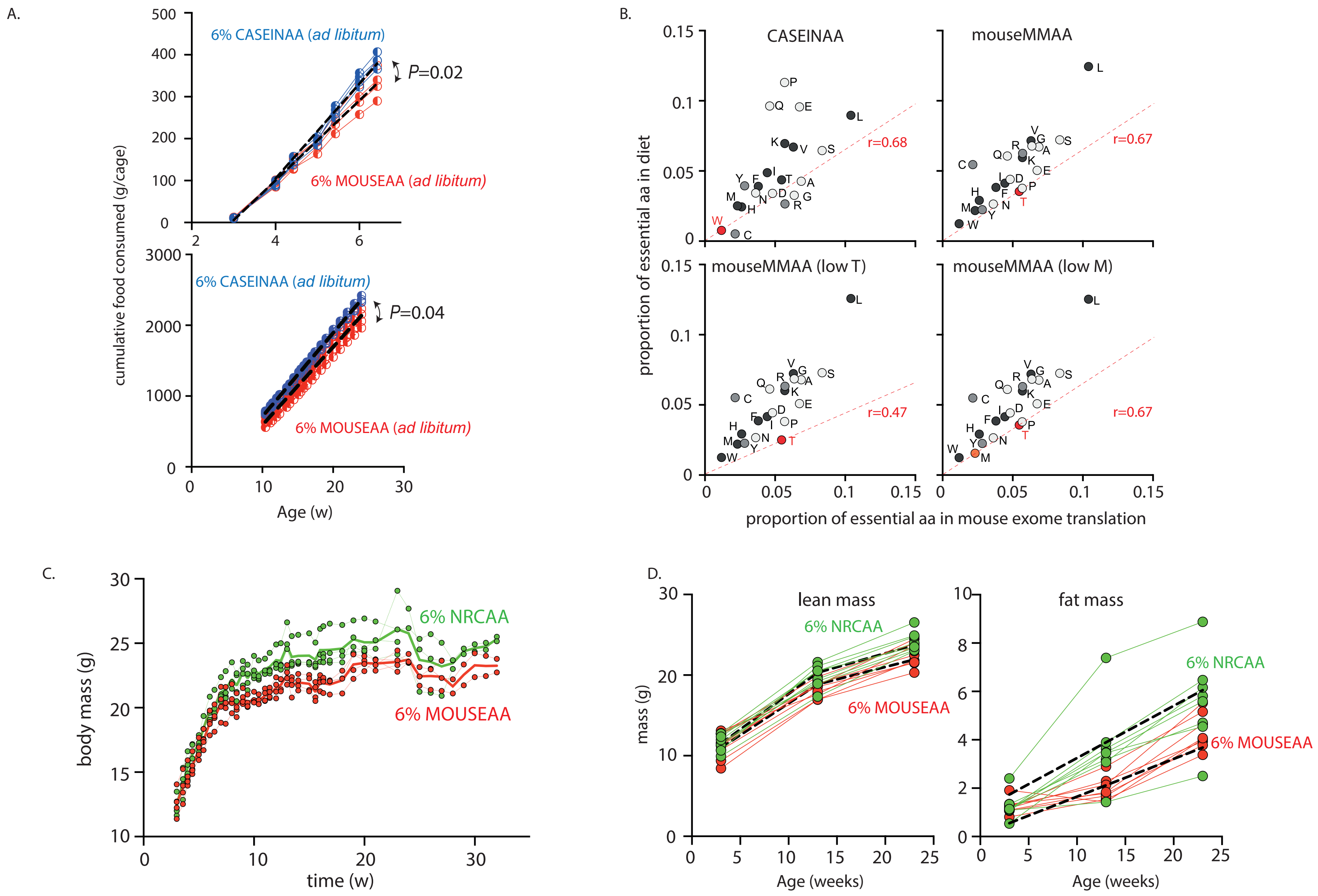

A.
6% CASEINAA (ad libitum)
P=0.02
6% MOUSEAA (ad libitum)
cumulative food consumed (g/cage)
6% CASEINAA (ad libitum)
P=0.04
6% MOUSEAA (ad libitum)
Age (w)
B.
CASEINAA
mouseMMAA
mouseMMAA (low T)
mouseMMAA (low M)
r=0.68
r=0.67
r=0.47
r=0.67
proportion of essential aa in diet
proportion of essential aa in mouse exome translation
C.
6% NRCAA
6% MOUSEAA
body mass (g)
time (w)
D.
lean mass
fat mass
6% NRCAA
6% MOUSEAA
6% NRCAA
6% MOUSEAA
mass (g)
Age (weeks)
Age (weeks)

Figure S7 Mouse growth (see also Figure 7)

(a) Mice with *ad libitum* access to MOUSEAA consumed ~15% less food than those given free access to CASEINAA (P=0.02, Linear model mixed effects), an effect that persisted during adulthood (P=0.04, Linear model mixed effects). Coloured points represent cumulative food consumption per cage, heavy dashed black lines show regression from statistical model. Data from a single trial. AA ratio, time and their interaction were main effects, while the effect of cage and its interaction with time assigned as random effects.
(b)Matching the mouse translated exome to CASEINAA or mmMOUSEAA ratio predicts that tryptophan or threonine (red point) is the limiting amino acid, respectively. Reducing T by 30% in mmMOUSEAA diet exaggerates the T limitation and is thus predicted to further reduce growth rate. However, reducing M by 30% is predicted to produce no growth difference from that of mMMAA since its excess should not be completely depleted (M reduction highlighted by orange point). Thus T should remain the limiting amino acid and growth should be unaffected. In each case the predicted limiting amino acid is highlighted in red and r indictaes the relative severity of the under-representation. Differences in r between diets are predicted to reveal relative growth rate differences for mice on different diets. Calculations are based on EAAs (dark grey points). Conditionally essential AAs are shown as mid-grey points and non-essentials as light grey points.
(c) Although initial linear growth of mice on NRCAA was indistinguishable from those fed MOUSEAA, the mice maintained on NRCAA accreted and maintained a greater body mass as growth rate slowed.
(d) Whole body MRI revealed the mass differences in panel (b) were largely due to an increased gain of fat mass gain (P=0.03), but not lean mass (P=0.06 for the interaction of how diet affected mass gain over time; linear mixed effects model). Measurements made at week 3, 13 and 23, corresponding to 0, 10 and 20 weeks of treatment. Coloured points represent cumulative tissue mass, heavy dashed black lines show regression from statistical model. Data from a single trial. AA ratio, time (log transformed for lean mass) and their interaction were main effects, while the effect of mouse and its interaction with time assigned as random effects.

Supplemental Tables

Table S1 Reference table for mouse experiments and reported phenotypes. See also Figures 5, 6 & 7.

	Dietary conditions	Growth rates	Water intake	Urinary urea production	Body composition	Energy expenditure and body temperature	Glucose tolerance and insulin tolerance	Bone parameters	Hepatic portal vein blood amino acid profile	Food intake
Expt. A	7.6% CASEINAA (protein; pair-fed)<br><br>7.6% CASEINAA (purified AAs; pair-fed)	Figure S5	Data not shown	Not done	Not done	Not done	Not done	Not done	Not done	Data not shown
Expt. B	6% CASEINAA (*ad libitum*)<br>6% MOUSEAA (*ad libitum*)<br>6% CASEINAA (pair-fed)<br>6% MOUSEAA (pair-fed)<br>8% CASEINAA (pair-fed)	Figure 5b Defined as the linear portion of growth immediately after weaning from week 3 to week 6.4. MOUSEAA improved growth rate by 31% over CASEINAA (pair fed)<br><br>Figure S5d, growth curve continued to end of	Figure 6a Mice fed CASEINAA consumed ~35% more water during the initial growth phase than those on MOUSEAA. Assessing water intake from both experiments together or from this trial alone support this	Not done	Figure 6c The profile of lean and fat mass accumulation was increased for mice fed MOUSEAA over those fed CASEINAA. *In vivo* magnetic resonance tomography imaging was performed on each mouse at weeks 3, 13 and 23	Not done	Not done	Not done	Figure S6b	Data not shown

		measurements at wk 24.	conclusion.		(corresponding to 0, 10 and 20 weeks exposure to diets)					
Expt. C	6% CASEINAA (ad libitum)<br>6% CASEINAA (pair-fed)<br>6% MOUSEAA (pair-fed)<br>6% mmMOUSEAA (pair-fed)<br>6% mmMOUSEAA (low T; pair-fed)<br>6% mmMOUSEAA (low M; pair-fed)<br>6% NRCAA (pair-fed)<br>6% PROTEOMEAA (pair fed)	Data not shown. MOUSEAA improved growth rate by 33% over CASEINAA (pair fed).<br><br>Figure 7 Growth rate effects of mice on mmMOUSEaa.	Data not shown.	Figure 6b Mice fed CASEINAA excreted a greater proportion of their dietary nitrogen intake as urinary urea than those on MOUSEAA. These data collected from mice singly housed in special metabolic cages at the end of the growth experiment (week 23, after mice were exposed to diets for 20 weeks)		Figure 6d Indirect calorimetry and movement were monitored. The only parameter to differ between food types was that CASEINAA mice were observed to generate more heat.<br><br>Data not shown There was no difference in any measured metabolic parameter between NRCAA and MOUSEAA fed animals.	Data not shown<br><br>No differences were detected between MOUSEAA or NRCAA-fed mice in glucose clearance for a glucose or insulin challenge	Figure 6e Bone parameters from right femurs of 23 week old mice.<br><br>Data not shown No differences in any of the bone parameters was detected when comparing mice fed NRCAA versus those on MOUSEAA.	Not done	Figure S6e With free access to food, mice on MOUSEAA voluntarily ate less than those on CASEINAA

Table S2. Amino acid ratios for each of the fly holidic diets. (See Experimental Procedures)

		MM1AA[1]			FLYAA			MM2AA			MM3AA			MM4AA		
		per litre (g)[2]	mM	ratio[3]	per litre (g)	mM	ratio	per litre (g)	mM	ratio	per litre (g)	mM	ratio	per litre (g)	mM	ratio
Essential amino acids																
phenylalanine	F	0.79	4.76	0.027	0.79	4.79	0.028	0.82	4.97	0.030	0.79	4.79	0.037	0.92	5.55	0.043
histidine	H	0.61	3.90	0.022	0.55	3.52	0.020	0.54	3.45	0.021	0.55	3.52	0.026	0.68	4.37	0.032
isoleucine	I	1.82	13.83	0.077	1.11	8.45	0.049	0.40	3.04	0.018	1.11	8.45	0.052	1.16	8.81	0.054
lysine	K	1.15	7.86	0.044	1.21	8.28	0.048	1.26	8.61	0.051	1.21	8.28	0.057	1.74	11.88	0.081
leucine	L	1.21	9.22	0.052	2.01	15.31	0.088	2.72	20.76	0.124	2.01	15.31	0.094	1.64	12.53	0.077
methionine	M	0.48	3.24	0.018	0.52	3.51	0.020	0.61	4.12	0.025	0.52	3.51	0.025	0.34	2.28	0.016
arginine	R	0.48	2.78	0.016	1.21	6.97	0.040	1.93	11.08	0.066	1.21	6.97	0.057	1.42	8.17	0.067
threonine	T	1.21	10.16	0.057	1.21	10.12	0.058	1.19	9.97	0.060	1.21	10.12	0.056	1.29	10.87	0.061
valine	V	1.69	14.47	0.081	1.33	11.35	0.065	0.94	8.03	0.048	1.33	11.35	0.062	1.34	11.43	0.063
tryptophan	W	0.30	1.48	0.008	0.20	1.00	0.006	0.18	0.90	0.005	0.20	1.00	0.010	0.44	2.16	0.021
Non-essential amino acids																
alanine	A	2.12	23.77	0.133	1.60	17.99	0.104	1.04	11.66	0.070	1.60	17.99	0.075	1.59	17.83	0.074
cysteine	C	0.03	0.25	0.001	0.37	3.01	0.017	0.76	6.30	0.038	0.37	3.01	0.017	0.26	2.18	0.012
aspartate	D	1.03	7.73	0.043	1.14	8.58	0.049	1.25	9.38	0.056	1.14	8.58	0.053	0.84	6.31	0.039
glutamate	E	1.51	10.29	0.058	1.34	9.10	0.052	1.14	7.75	0.046	1.34	9.10	0.063	1.82	12.38	0.085
glycine	G	1.94	25.78	0.144	1.32	17.64	0.102	0.69	9.19	0.055	1.32	17.64	0.062	1.08	14.41	0.051
asparagine	N	1.03	7.79	0.044	1.01	7.65	0.044	1.00	7.55	0.045	1.01	7.65	0.047	0.84	6.36	0.039
proline	P	0.91	7.89	0.044	1.10	9.57	0.055	1.29	11.23	0.067	1.10	9.57	0.052	0.56	4.90	0.026
glutamine	Q	1.51	10.35	0.058	0.99	6.80	0.039	0.48	3.30	0.020	0.99	6.80	0.046	1.82	12.45	0.085
serine	S	1.15	10.94	0.061	1.70	16.17	0.093	2.19	20.86	0.125	1.70	16.17	0.079	0.76	7.23	0.036
tyrosine	Y	0.42	2.32	0.013	0.66	3.67	0.021	0.95	5.22	0.031	0.66	3.67	0.031	0.84	4.63	0.039
Sum		21.39		1	21.39		1	21.39		1	21.39		1	21.34		1

[1] Amino acid (AA) ratios: MM(1)AA = mismatch (1); FLY = matched to *in silico* generated exome.
[2] AA masses shown total to 21.4g/l. Other masses used in this study were generated by scaling the same ratio of amino acid masses to achieve the target total mass.
[3] Ratio represents the molar proportions in the media

Supplemental Experimental Procedures

Holidic media for Drosophila

Holidic media were made according to (Piper et al., 2014) with appropriate substitutions for each of the different amino acid ratios (Table S2 and File S1). We found that during storage of the stock solutions of the non-essential amino acids in the ratios for MM2AA and FLYAA, cysteine dropped out of solution. For these media, we therefore omitted C from the combined stock and made a different stock solution that was added separately (see File S1). Three of the amino acids (I,L,Y) are added to the medium before autoclaving for reasons of poor solubility. To address the concern that there may be partial degradation of these amino acids, we compared the effect on development timing and lifespan of adding these before and after autoclaving. We found no evidence for an effect and so where required, data were combined.

Mouse strain, housing and diets

We used two inbred strains from the Jackson laboratory (C57BL/6J and C3H) to generate our C3B6F1/J females used in experiments.

Due to regulatory restrictions on the use of mice, our measurements of different physiological parameters were divided between different cohorts of mice (Experiments B and C outlined in Table S1). For the comparison of MOUSEAA v CASEINAA growth curves, there was no discernible effect of trial (P=0.37) on the influence of diet (P=0.84), time (P=0.72) and their interaction (P=0.88) (Generalised Linear Model, mixed effects). Thus, we have strong evidence that these two cohorts are physiologically comparable.

To enforce pair-feeding, the mass of pellets consumed daily by the group consuming the least amount of food was supplied to the rest of the groups at subsequent time point(s), until cumulative food consumption was equalized between all pair-fed groups. For the *ad-libitum*-fed groups, food intake was measured for each cage separately (mass of pellets on the cage's feeders before and after feeding period).

To ensure mouse growth was protein limited, we empirically determined the crude protein (CP) level for use at 6-8% (Figure S5b). In all cases, diets were isocaloric, with constant micro- and macro-nutrient compositions (except for AA ratios) and with a constant total mass of AAs.

Amino acid usage similarity for protein rankings

We computed the distance between each protein and the average AA content for the in silico translated exome (sum of squared differences of AA ratios according to: sqrt(sum over all AAs of ((ratio in protein - mean ratio in translated exome)/sd ratio in translated exome)^2)). We then ranked all proteins by their distance such that lower rankings are most similar to the exome. The probability of a tissue-specific gene set having a rank-sum significantly higher or lower than that yielded by chance when randomly sampling a similar sized gene set from the ranked list was assessed using Catmap (Breslin et al., 2004).

Amino acid composition determination for flies and mice from published data

To generate the amino acid ratios used in the fly PROTEOMEAA profile, the raw data from a published proteomic study (Sury et al., 2010) were reanalyzed. Protein identification was carried out using MaxQuant (Cox and Mann, 2008) version 1.4.1.2 using the integrated Andromeda search engine (Cox et al., 2011). Raw data were searched against the FlyBase gene translations fasta database (release FB2008_05) containing 21,070 entries. For the protein identification from the raw proteomics data, the fasta database was automatically complemented with sequences of contaminating proteins by MaxQuant. For peptide identification, cysteine carbamidomethylation was set to "fixed" and methionine oxidation and protein N-terminal acetylation as "variable" modifications. For *in silico* digestion the enzyme was set to "LysC/P" allowing for cleavage after lysine, also when followed by proline with a maximum of two missed cleavages. The minimum number of peptides and razor peptides for protein identification was 1; the minimum number of unique peptides was 0. Protein and peptide identification was performed using a false discovery rate (FDR) of 0.01. The "second peptide" option was on allowing for the identification of co-fragmented peptides. In order to transfer identifications to non-sequenced or non-identified peptides in the separate raw files, the option "Match between runs" was turned on using a "Match time window" of 1 min and "Alignment time window" of 20 min. Protein copy numbers per 1g of total protein were derived using the label free quantification based total protein approach (TPA) (Wiśniewski and Rakus, 2014; Wiśniewski et al., 2012). Protein copy numbers were calculated from the protein intensity data from MaxQuant's ProteinGroups.txt output file. As MaxQuant reports

identified proteins in groups when the presence of individual protein sequences cannot be unambiguously inferred, only the first protein ID in a protein group was used. Only proteins that had recorded mass spectrometric intensity more than zero were used for analysis. The combined (heavy and the light SILAC) signal from the proteins was used. Protein copy numbers (per 1g of total protein) were calculated by dividing the protein intensity values by the total intensity of all identified proteins. This value, which corresponds to protein concentration measured in mol per gram total protein, was divided by the protein MW arriving at protein copy numbers. Next, the protein copy number was multiplied by the number of each of the 20 amino acids arriving at the total amino acid numbers for each protein. These latter values were used to calculate the total amino acid quantities in the whole fly and the respective weighted amino acid proportions.

Fly diet preference and feeding assays

Holidic diet choice assay: The apparatus used for the dietary preference assays is a modified version of that described in (Cooper, 1960), which enables the experimental flies to choose between 4 vials containing a medium. We used a prototype scaled-down version of the chamber for 40 once-mated female flies, and tested diet preference, population effects and time-monitoring to determine the experimentally optimal conditions. The chamber lighting was diffuse and the apparatus rotated hourly by 90° to avoid spatial or other environmental cues. Each assay allowed flies the choice of two holidic media differing in their amino acid ratio only, with pairs of foods positioned oppositely in the apparatus. The total mass of amino acids was fixed at 21.4g/l.
Prior to the assay, mated females were maintained for three days on holidic medium without amino acids. Flies were acclimatized in the chamber and allowed time to settle on their diet of choice overnight (approximately 14 hours). The location of flies was counted hourly over ~8 hours and the data for 11am is presented. The effect shown was apparent at each time point measured. The Food Preference Index (FPI) was calculated as: (n flies on surface of food A – n flies on surface of food B) / (n flies on surface of food A + n flies on surface of food B). The DPI was calculated as the average of all data collated for the 8h assay.

Sugar / yeast choice assay: Dahomey flies were reared in yeast-based food containing (per liter of water: 80g sugar cane molasses, 22g sugarbeet syrup, 8g agar, 80g corn flour, 10g soya flour, 18g yeast extract, 8ml propionic acid, 12 ml nipagin (15% in EtOH)). Upon emerging as adults, groups of 3-5 days old flies (15 females and 5 males) were transferred to fresh yeast based food or holidic medium. After 72h, the flies were tested for nutrient choice. Two-choice color feeding preference assays were performed as described in (Ribeiro and Dickson, 2010). Flies were given the choice between sucrose mixed with red colourant (20mM sucrose; 7.5mg/ml agarose; 5mg/ml Erytrosin B (Sigma-Aldrich 198269); 10% PBS) or yeast mixed with blue colourant (10% yeast (SAF instant yeast); 7.5mg/ml agarose; 0.25mg/ml Indigo carmine (Sigma-Aldrich 131164); 10% PBS) medium. After visual inspection of the abdomen, each female fly was scored as having eaten sucrose (red abdomen), yeast (blue abdomen), or both (red and blue or purple abdomen) media. The yeast preference index (YPI) for the whole female population in the assay was calculated as follows: (nblue yeast – nred sucrose)/(nred sucrose + nblue yeast + nboth).

Measuring fly development
Eggs were collected overnight from young, age-matched flies and incubated for 24 h at 25°C, at which point first instar larvae were picked onto test media and returned to 25 °C. The number of larvae of each developmental stage were scored at 17, 21, 25, 90, 114, 138, 258 and 330h after egg laying. Adult eclosion was scored daily at 24-hour intervals. Body mass was measured for pairs of newly emerged flies, and the mean for each pair used for analysis. For wing length, fixed wings were measured from the edge of the distal tip to the edge of the alula using a Leica microscope M165FC, mounted with a DFC420 camera.

Fly uric acid and TAG measurements
10-day old mated females (8 days of treatment) were transferred to fresh vials containing holidic media. After 16 hours, flies and medium were removed from the vial, and the uric acid content of the empty vials dissolved in 2 ml of 0.1 M sodium glycinate buffer (pH 9.2). Uric acid was quantified spectrophotometrically using the Amplex® Red Uric Acid kit (Life Technologies). For TAG determinations, 25 10-day old females per diet were anesthetized using CO2, divided into 5 groups of 5 and homogenized in 1 ml 0.05% Tween 20 and 50ml used for analysis with the Triglyceride Infinity reagent (ThermoScientific). Levels were normalized to total protein.

Mouse physiological measurements
Body fat content was determined by *in vivo* magnetic resonance tomography imaging (time domain (TD) NMR) at weeks 3, 13 and 23 of age (0, 10, and 20 weeks of treatment respectively). The fat content measured by TD-NMR included all types of fat in the body and the lean content included all kind of muscle tissue and free fluid. Typically, the fat, lean and free fluid content amounted to ~92-96% of the total body mass.
Indirect calorimetry and movement were monitored for singly housed mice in purpose built cages (Phenomaster, TSE systems) maintained at 22-23°C. By constantly monitoring the levels of oxygen and carbon dioxide in the incoming and outgoing air, the RER and heat production can be calculated. Laser beam obstruction was used to count incidences of horizontal and vertical movements. Before starting the measurements, mice were housed in training cages in order to acclimatize to single housing, hanging water bottles, special drinking nipples, and hanging food baskets. Mice were intensively checked for their drinking and eating behavior to ensure they accepted the new drinking and feeding system. After the time in the training cages, the mice were acclimatized for 24 hours in the metabolic cages. Data was collected for 48 hours.
Urea in urine was measured in metabolic cages (Tecniplast) that are designed to measure all consumed food and water as well as all excreta. Animals were acclimatised to the cages for 24 hours and measurements made during an additional 24 hours. Urine urea was measured using the Urea Assay Kit (Sigma) and used to calculate the total nitrogen lost in urine. This was expressed as a proportion of dietary nitrogen consumed.
Glucose tolerance tests (GTT) were performed at 23 weeks of age. After an approximately 16 hour fasting period with free access to drinking water, mice were intraperitoneally injected with 2 g of glucose per kg body weight. Blood glucose was measured before, 15, 30, 60 and 120 minutes after glucose injection.
The insulin tolerance test (ITT) was performed at 24 weeks of age. Mice were intraperitoneally injected with 0.75 units of insulin (in 0.9% NaCl) per kg body weight. Blood glucose was measured before, 15, 30 and 60 minutes after insulin injection. ITT mice were fed a small meal of 1 gram per mouse approximately 2 hours prior to being tested.

Portal vein plasma metabolite analysis
Portal vein blood samples were harvested from pair-fed female C3B6F1/J mice at 23 weeks of age following a 1 g meal and 2 hour food deprivation. Mice were euthanized using CO2, dissected, and portal vein blood collected in EDTA tubes. These were spun for 15 minutes at 1,200 rpm (4°C). Samples were then flash-frozen in liquid nitrogen and thawed once for an LC-MS metabolomics analysis performed by the Finnish Institute for Molecular Medicine (FIMM).

Mouse tissue and bone measurements
At 23 weeks of age, mice were euthanized using CO2 and organs were immediately harvested and weighed using a Sartorius ED423S-CS microscale. For bone density measurements, right femur bones from 23 week old mice were collected, placed in 10% PBS saline solution, and stored at -80°C. Before scanning, samples were thawed and hydrated overnight in a saline solution at 5°C. Femurs were scanned with a high resolution µCT scanner (SkyScan 1176, Bruker, Belgium) with an isotropic voxel size of 8.8 μm^3. The x-ray settings for each scan were 50 kV and 500 µA using a 0.5 mm aluminum filter. All scans were performed over 360 degrees with a rotation step of 0.3 degrees and a frame averaging of 1. Images were reconstructed and analyzed using NRecon and CTAn software, respectively (Bruker, Belgium). Trabecular and cortical bone regions of distal femurs were selected with reference to the growth plate (0.44-2.2 and 2.2-2.64 mm from growth plate for group 1, respectively. Size and position of bone regions were adjusted for the other groups depending on femur length). Bone mineral density was determined based on calibration with two phantoms of known density (Bruker, Belgium), which were scanned under the same conditions as the bone samples.

Supplemental File

File S1. Templates for fly holidic diets and calculator for identifying limiting AA. (See Experimental Procedures).
Tab 1: Using the consumer's average AA proportion and the AA proportions in the diet, this calculator identifies the essential AA predicted to be most limiting in the diet. Text in blue is to be modified by the user, all other cells are calculated automatically. The AA in 1-fold excess in column T is the theoretically most limiting dietary AA.

Tab 2: AA proportions used to make the fly diets described in this study
Tab 3: Description of the stock solutions used for each AA ratio described in this study.
Tab 4: Recipes used to make the fly holidic media used in this study. Separate diets are itemised for each of the AA ratios and deliver 21.4g total mass of AA per litre. For details of remaining stock solutions and a more extensive description of the method to make the diet, See (Piper et al, 2014)

Supplemental References

Cooper, D.M. (1960). Food preferences of larval and adult Drosophila. Evolution *14*, 41–55.

Cox, J., and Mann, M. (2008). MaxQuant enables high peptide identification rates, individualized p.p.b.-range mass accuracies and proteome-wide protein quantification. Nat. Biotechnol. *26*, 1367–1372.

Cox, J., Neuhauser, N., Michalski, A., Scheltema, R.A., Olsen, J.V., and Mann, M. (2011). Andromeda: a peptide search engine integrated into the MaxQuant environment. J. Proteome Res. *10*, 1794–1805.

Itskov, P., Moreira, J.-M., Vinnik, E., Lopes, G., Safarik, S., Dickinson, M., and Ribeiro, C. (2014). Automated monitoring and quantitative analysis of feeding behaviour in Drosophila. Nature Communications *5*, 4560.

Piper, M., Blanc, E., Leitão-Gonçalves, R., Yang, M., He, X., Linford, N., Hoddinott, M., Hopfen, C., Soultoukis, G., Niemeyer, C., et al. (2014). A holidic medium for Drosophila melanogaster. Nature Methods *11*, 100–105.

Ribeiro, C., and Dickson, B. (2010). Sex Peptide Receptor and Neuronal TOR/S6K Signaling Modulate Nutrient Balancing in Drosophila. Current Biology *20*, 1000–1005.

Sury, M.D., Chen, J.-X.X., and Selbach, M. (2010). The SILAC fly allows for accurate protein quantification in vivo. Mol. Cell Proteomics *9*, 2173–2183.

Wiśniewski, J.R., and Rakus, D. (2014). Multi-enzyme digestion FASP and the “Total Protein Approach”-based absolute quantification of the Escherichia coli proteome. J Proteomics *109*, 322–331.

Wiśniewski, J.R., Ostasiewicz, P., Duś, K., Zielińska, D.F., Gnad, F., and Mann, M. (2012). Extensive quantitative remodeling of the proteome between normal colon tissue and adenocarcinoma. Mol. Syst. Biol. *8*, 611.
